# The amphibians and reptiles of Mindanao Island, southern Philippines, II: the herpetofauna of northeast Mindanao and adjacent islands

**DOI:** 10.3897/zookeys.624.9814

**Published:** 2016-10-17

**Authors:** Marites B. Sanguila, Kerry A. Cobb, Cameron D. Siler, Arvin C. Diesmos, Angel C. Alcala, Rafe M. Brown

**Affiliations:** 1 Biodiversity Informatics and Research Center, Father Saturnino Urios University, San Francisco St., 8600 Butuan City, Philippines; 2 Biodiversity Institute and Department of Ecology and Evolutionary Biology, University of Kansas, Lawrence, KS 66045-7561, USA; 3 Sam Noble Oklahoma Museum of Natural History and Department of Biology, University of Oklahoma, Norman, OK 73072-7029, USA; 4 Herpetology Section, Zoology Division, Philippine National Museum, Rizal Park, Burgos St., Ermita 1000, Manila, Philippines; 5 Angelo King Center for Research and Environmental Management, Silliman University, Dumaguete City 6200, Philippines

**Keywords:** Agusan del Norte, Agusan del Sur, Balatukan, Biodiversity, Camiguin Sur, Conservation, Dinagat, Hilong-hilong, Lumot, Magdiwata, Misamis Oriental, Siargao, Surigao del Norte, Surigao del Sur

## Abstract

We summarize all available amphibian and reptile species distribution data from the northeast Mindanao faunal region, including small islands associated with this subcenter of endemic vertebrate biodiversity. Together with all publicly available historical information from biodiversity repositories, we present new data from several major herpetological surveys, including recently conducted inventories on four major mountains of northeast Mindanao, and adjacent islands of Camiguin Sur, Dinagat, and Siargao. We present species accounts for all taxa, comment on unresolved taxonomic problems, and provide revisions to outdated IUCN conservation status assessments in cases where our new data significantly alter earlier classification status summaries. Together, our comprehensive analysis of this fauna suggests that the greater Mindanao faunal region possesses distinct subcenters of amphibian and reptile species diversity, and that until this area is revisited and its fauna and actually studied, with on-the-ground field work including targeted surveys of species distributions coupled to the study their natural history, our understanding of the diversity and conservation status of southern Philippine herpetological fauna will remain incomplete. Nevertheless, the northeast Mindanao geographical area (Caraga Region) appears to have the highest herpetological species diversity (at least 126 species) of any comparably-sized Philippine faunal subregion.

## Introduction

**Figure 1.**
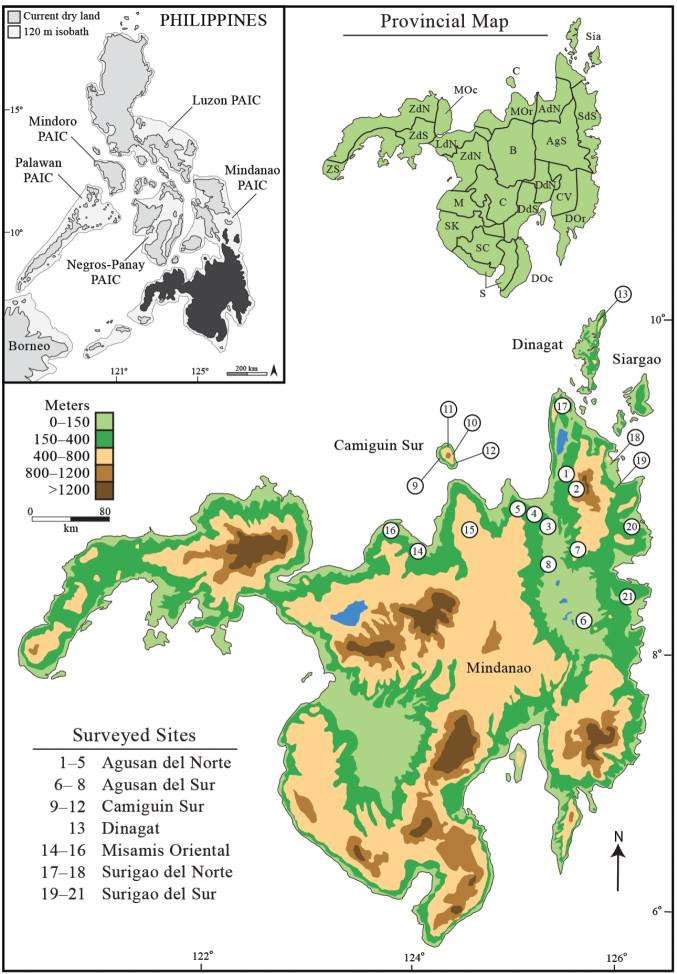
Map of Mindanao Island in relation to the remaining Philippine archipelago (inset). Numbered study sites correspond to those listed in Table 1. Colored contours correspond to elevation (elevation key). Dinagat Island (Site 13) is often presumed to possess the same herpetofauna as Siargao (the small island to its southeast), whereas the fauna of Camiguin Sur Island (Sites 9–12) has been assumed to be somewhat distinct from that of Mindanao. Neither of these generalizations has been examine critically (Brown and Alcala 1967, 1970; Ross and Lazell 1991; Nuñeza and Galorio 2015). Province names are abbreviated with unique letter combinations used in text and tables (provincial map key), and numbered sites are assigned to province (surveyed sites key) for clarity.

Recent efforts to conduct comprehensive herpetological surveys of the various islands of the Philippines have provided near-complete estimation of the amphibian and reptile diversity and endemism of several islands, mountain ranges, or other conspicuous geographical subcenters of diversity in the northern reaches of the archipelago (Brown et al. 2013a). These efforts have focused on the northern Babuyan and Batanes islands (Oliveros et al. 2011), the large northern island of Luzon (McLeod et al. 2011; Siler et al. 2011a; Brown et al. 1996, 2000a, 2012a, 2013a; Devan-Song and Brown 2012), and central island groups (Brown and Alcala 1961, 1964, 1986; Siler et al. 2012a), but little recent activity has documented other parts of the archipelago, especially the western island of Palawan, the Sulu Archipelago, and portions of the large southern island of Mindanao (Leviton 1963; Brown and Alcala 1970a; Peterson et al. 2008; Jones and Kennedy 2008; Siler et al. 2009). Mindanao supports high levels of herpetological diversity (Taylor 1920a,b, 1921, 1922a,b; Brown and Alcala 1970; Brown et al. 2013a; Diesmos et al. 2015) and considerable endemism, despite its close proximity to the larger landmasses of Sundaland (Borneo, Sumatra, and Java) and Wallacea (Sulawesi; Fig. 1). The current lack of information relating to Mindanao’s herpetological diversity, can be attributed to several recent decades of increasing bureaucratic and security-related logistical obstacles to research, combined with earlier, possibly incorrect impression of some biologists that its herpetofauna is reasonably well known (Taylor 1920a, 1921, 1922a; Inger 1954; Brown and Alcala 1970, 1978, 1980; Ross and Lazell 1990).

The perception that southern portions of the Philippine archipelago are sufficiently studied and/or reasonably understood may derive in part from proximity-based expectations of faunal similarity between Mindanao, Sulu, and Palawan versus the islands of Sundaland and Wallacea (Boulenger 1920, Inger 1954, 1999, Leviton 1963a, Brown and Alcala 1970). Alternatively, some of this perception stems from the fact that Taylor worked extensively in the region, lived on Mindanao for over two years, collected more than 2,000 specimens from 1912–1915, and returned to survey sites in southern portions of the Philippines periodically until 1924 (Taylor 1975; Duellman 2015). He published widely on southern Philippine herpetofauna (Taylor 1917a,b, 1918a,b, 1919, 1920a,b, 1921, 1922a,b,c,d, 1923, 1928, 1960, 1965, 1975), with his own collections forming the basis for much of this work (Taylor 1975). With this impressive body of work as an early 20^th^ Century starting point, later workers may have been motivated to focus elsewhere in their efforts to characterize new faunas (i.e., central and northern portions of the archipelago; Inger 1954; Leviton 1963a; Brown and Alcala 1970). As a result, few modern systematic treatments have revisited the southern Philippine endemics with modern collections-based technologies (fresh specimens, integrative techniques, ecological information, life history studies, acoustic data, genetic resources, or molecular data); in fact, many recent attempts at archipelago-wide revisions of Philippine fauna have had no alternative but to base decisions regarding species diversity (e.g., Diesmos et al. 2015) and conservation status (IUCN 2016) on Taylor’s original specimens, collected over a century ago (Leviton 1964a,b,c,d, 1965a,b, 1967, 1968; Brown and Alcala 1978, 1980, 1994, 1997; Brown and Guttman 2002; Siler et al. 2011b).

Our prevailing perspective on patterns of endemism and subdivision of terrestrial biodiversity in the archipelago includes a general acceptance of a model of diversification based on late Pleistocene sea level oscillations and the generation of periodic land connectivity; this has been termed the Pleistocene Aggregate Island Complex (PAIC) paradigm (Inger 1954; Voris 2000; Brown and Diesmos 2009; Brown et al. 2013b). According to this model for understanding patterns of diversity (Brown and Diesmos 2009) and processes of diversification (Siler et al. 2010; Esselstyn and Brown 2009; Oaks et al. 2013; Brown et al. 2013b) the Mindanao PAIC is one large biogeographically significant sub-province within the archipelago, with Mindanao proper having been connected to the neighboring islands (Dinagat, Siargao, Samar, Leyte, Bohol, Biliran, etc.) of today by land bridges during the Pleistocene (Taylor 1928; Inger 1954; Voris 2000; Brown and Diesmos 2009). Because of this perspective, herpetologists have assumed that when sea levels were low during the Pleistocene, the emergence of land bridges allowed faunal exchanges between connected landmasses, producing highly endemic faunas within PAICs (Brown and Diesmos 2001, 2009) and archipelago-wide diversity generated by prevailing species boundaries between PAICs. Augmenting the PAIC paradigm, appreciation for recent geological events, such as accretion of paleoisland precursors along identified suture zones (Yumul et al. 2003, 2009), and repeated bouts of colonization (Siler et al. 2011b; Brown et al. 2013b; Brown and Siler 2013; Justiniano et al. 2015; Brown 2015) are assumed to contribute to the evolutionary process of diversification via within- and among-PAIC isolation. Habitat fragmentation, ecological gradients, and barriers in ecological suitability are assumed to have contributed as well, although these ideas are less studied potentialities for future research programs (Steppan et al. 2003; Esselstyn and Brown 2009; Brown et al. 2010a; Esselstyn et al. 2011; Roberts et al. 2011; Sanguila et al. 2011; Gonzales et al. 2014; Hosner et al. 2014). Together, these geological, geographic and faunal distribution patterns contribute to the Philippine archipelago’s recognition as a “model” archipelago for the study of evolutionary processes of biotic diversification (Brown et al. 2013b). How is it, then, that the herpetofauna from the southern portion of this model island archipelago remains so poorly understood, with the many of Mindanao’s endemic species absent in modern molecular phylogenetic studies, and fundamental phylogeographic studies of even its most common species sorely lacking (but see McGuire and Kiew 2001; Evans et al. 2003; Sanguila et al. 2011; Siler et al. 2011b; Brown and Siler 2013).

Mindanao was formed by the accretion of the island-arc related to the eastern-central block and the western continental Zamboanga peninsula block, separated by the active Sindangan-Cotabato-Daguma lineament (Yumul et al. 2003). The eastern portion of the island was previously divided into the high mountains of the Pacific Cordillera from the Central Cordillera, separated by the Agusan-Davao trough (Yumul 2004, 2009). Many of the large isolated mountains in central Mindanao are separated from each other by substantial stretches of low-lying areas (Hall 1998) that were formed as a result of collision, accretion and subduction events over the past ten million years (Hall 1996, 1997; Yumul et al. 2003, 2009). This highly dynamic geological history suggests the possibility of faunal limits to dispersal within early paleoislands, a mechanism that might possibly have contributed to the diversification of the first amphibian and reptile lineages that colonized Mindanao (Brown and Alcala 1970; Brown and Guttman 2002; Evans et al. 2003; Sanguila et al. 2011; Brown et al. 2013b; Barley et al. 2014).

Previous studies have described the possibility of an “island-hopping” mode of dispersal across paleoislands to explain colonization of the eastern Philippine island arc (Diamond and Gilpin 1983; Brown and Guttman 2002; Jones and Kennedy 2008; Brown et al. 2009; Oliveros and Moyle 2010; Roberts et al. 2011; Sanguila et al. 2011; Brown and Siler 2013; Gonzales et al. 2014); many of these conceived west-to-east dispersal/diffusion route of colonization (Brown and Guttman 2002; Evans et al. 2003; Brown et al. 2009) from the western continental Zamboanga peninsula, as evidenced by the series of island formations distributed across the southern island (Hall 2002; Yumul 2004; Gonzales et al. 2014).

Because of obstacles to biologists’ access to parts of Mindanao, our current understanding of the island’s herpetofauna comes in large part from the historical works of Boulenger (1882, 1884, 1920), van Kampen (1923), Smith (1930, 1935), as well as the synthetic works of Taylor (1920a,b, 1921, 1922a,b), Leviton (1963a), Brown and Alcala (1970, 1978, 19780), and a few, scattered and site-specific studies (e.g., Smith 1993a,b; Ross and Lazell 1990). These have been subsidized by recent inventories from the eastern (Delima et al. 2006, 2007; Ates et al. 2009; Relox et al. 2011), central (Beukema 2011), and western (Nuñeza et al. 2009) portions of the island. Each are welcomed additions but most have been constrained in scope and lacking biogeographical context.

In this paper, we take what we hope will be a first step towards ameliorating Mindanao’s herpetological information shortage, by initiating the second study in a series of attempts towards a comprehensive review of the herpetofauna of the island. In this paper, we focus on the regional diversity and endemism of amphibians and reptiles from one subcenter (northeast Mindanao) of the biogeographically distinct Mindanao PAIC. We present species accounts using data from our own intensive herpetological surveys of northeast Mindanao and its adjacent islands, and provide notes on each species’ microhabitats and natural history. To provide a biogeographical synthesis, we include historical museum records from all accessible biodiversity repositories. The anticipated result will be a new opportunity to review numerous unresolved taxonomic problems, provide a new standardized reference for species distributional data, a much needed biogeographical reconsideration, and a platform from which biodiversity specialists can undertake revisions of the conservation status of the poorly understood herpetofauna of the southern Philippines.

## Materials and methods

We surveyed amphibian and reptile diversity at four major sites in Surigao del Norte, Surigao del Sur, Misamis Oriental, Agusan del Norte and Agusan del Sur provinces (Camiguin Sur, Dinagat and Siargao islands; Table 1) using standardized sampling techniques (Heyer et al. 1994) and specimen collection and preservation methodology (Brown et al. 2000a, 2012a, 2013a; ASIH 2004; Siler et al. 2011a; Simmons 2015). Our most recent surveys (March–April, 2009; June–August, 2012) involved intensive elevational transects on Mts. Balatukan and Lumot, Misamis Oriental Province and Mts. Magdiwata and Hilong-hilong, Agusan del Norte Province (Fig. 1). Surveys were conducted in early mornings, mid-day, afternoons, and evenings by experienced teams of four to eight individuals, sampling a wide variety of habitat types within each general study location. Habitats included dry forest on ridges, moist ravines, forest trails at all elevations, dry intermittent streambeds, small streams, seaps and swampy areas, large rivers, forest gaps and edges, and grassy open areas. Investigators at each sampling location made extensive surveys of each area (on foot) to ascertain habitat types and then visited each at varying times of the day. Nocturnal searches (1800–2400 hr) were conducted at each habitat type, within each sampling site, on dry and rainy nights. By concentrating field survey efforts to span the end of the dry season and the beginning of the rainy season (June–August) we were able to assure that each habitat type at each location was sampled under differing atmospheric conditions.

**Table 1.** Northeast Mindanao faunal region sites included in this study (where herpetological specimens have been collected and/or observations have been recorded). Numbered sites correspond to Figure 1 (map; note that some sites are included under a single number in Figure 1 in cases of close proximity) and coordinates and elevation are included when available. * = sites georeferenced for this study; ** = combined extremely proximate sites into one set of coordinates.

Site	Province	Municipality	Locality	Elevation (masl)	GPS Coordinates
1	Agusan del Norte	Cabadbaran	West of Mt. Hilong-hilong Peak, San Antonio & Balang-balang	91–518	9.09551N, 125.702E
1a	Agusan del Norte	Cabadbaran	Mt. Hilong-hilong W and SW of peak	610–853	9.09758N, 125.676E
1b	Agusan del Norte	Cabadbaran	Mt. Hilong-hilong SW and S side of peak	1067–1417	9.07981N, 125.696E**
1c	Agusan del Norte	Cabadbaran	Mt. Hilong-hilong, Taguibo and Dalaydayan River, S side of peak	1067–1524	8.98638N, 125.620E*
1d	Agusan del Norte	Cabadbaran	Mt. Hilong-hilong, S side of peak	1524–1829	9.07981N, 125.696E**
2	Agusan del Norte	Remedios T. Romuladez	Mt. Magdiwata, Mt Hilong-hilong, Balang-balang	101	9.05576N, 125.628E
2a	Agusan del Norte	Remedios T. Romuladez	Agay River, Barangay San Antonio; Bato-batohon	320	9.07663N, 125.655E
2b	Agusan del Norte	Remedios T. Romuladez	Coconut Plantation, Mt. Hilong-hilong	170	9.06490N, 125.641E
2c	Agusan del Norte	Remedios T. Romuladez	Eye Falls, Intersection of Dayhopan and Agay Rivers, Mt. Hilong-hilong	470	9.07520N, 125.664E
2d	Agusan del Norte	Remedios T. Romuladez	May Impit, Mt. Hilong-hilong	900	9.06250N, 125.672E
2e	Agusan del Norte	Remedios T. Romuladez	May Impit, Mt. Hilong-hilong	1130	9.62220N, 125.677E
2f	Agusan del Norte	Remedios T. Romuladez	May Impit, Mt. Hilong-hilong	1150	9.06595N, 125.681E
3	Agusan del Norte	Butuan City	Butuan City	6	8.94753N, 125.540E
4	Agusan del Norte	Buenavista	Barrio Matabao	4	8.96448N, 125.423E
5	Agusan del Norte	Nasipit	Along Highway between Barangay Libertad and Amontay	12	8.97482N, 125.361E
6	Agusan del Sur	Bunawan	Agusan Valley, Bunawan	68	8.17877N, 125.998E
6a	Agusan del Sur	Bunawan	Barangay San Marcos	23	8.22238N, 125.932E
7	Agusan del Sur	San Francisco	San Francisco	30	8.50897N, 125.969E
7a	Agusan del Sur	San Francisco	Barangay Bayugan II, Mt. Magdiwata	300–600	8.47308N, 125.986E
7b	Agusan del Sur	San Francisco	Barangay Kaimpugan, Agusan Marsh	33	8.40361N, 125.877E
8	Agusan del Sur	Talacogon	Talacogon	24	8.33333N, 125.833E
9	Camiguin	Catarman	Mt. Mambajao, SW side of peak	0–494	9.17120N, 124.724E*
9a	Camiguin	Catarman	Mt. Mambajao, SW side of peak	518–975	9.17120N, 124.724E*
9b	Camiguin	Catarman	Mt. Mambajao, SW side of peak	1036–1372	9.17120N, 124.724E*
9c	Camiguin	Catarman	Tuasan Falls	*NA*	9.15880N, 124.658E*
10	Camiguin	Mahinog	Mahinog Town	0	9.15000N, 124.783E
10a	Camiguin	Mahinog	Barrio Benone, Sitio Malabon	0	9.14666N, 124.793E
11	Camiguin	Mambajao	Mambajao town, along roadside	0–369	9.24753N, 124.716E
11a	Camiguin	Mambajao	0.6 km NE of Katibawasan Falls	375–853	9.21216N, 124.730E
11b	Camiguin	Mambajao	Mt. Hibok-hibok, NW side of Nasawa Crater	518–1113	9.18726N, 124.696E
11c	Camiguin	Mambajao	Balintawak St., Cabua-an Resort	43	9.20960N, 124.767E
11d	Camiguin	Mambajao	Barangay Balbagon	0	9.24387N, 124.737E
11e	Camiguin	Mambajao	Barangay Pandan, Sitio Kampana	1050	9.178016N, 124.71E
11f	Camiguin	Mambajao	Barangay Pandan, Sitio Pamahawan	707	9.192516N, 124.70E
11g	Camiguin	Mambajao	Ardent Hotspring	202	9.22662N, 124.688E
12	Camiguin	Guinsiliban	Barangay Cabuan	0–150	9.12444N, 124.781E
13	Dinagat Islands	Loreto	Loreto, Kawayanan	255	10.3500N, 125.616E
13a	Dinagat Islands	Loreto	Barangay Esperanza	5–116	10.3816N, 125.616E
13b	Dinagat Islands	Loreto	Barangay San Juan	26–72	10.3586N, 125.580E
13c	Dinagat Islands	Loreto	Barangay Santiago, Mt. Cambinlin	*NA*	10.3436N, 125.618E
14	Misamis Oriental	Cagayan de Oro	Cagayan de Oro City	5	8.48300N, 124.650E
15	Misamis Oriental	Gingoog	Barangay Civoleg, Mt. Lumot, Camp 2	1236	8.69590N, 125.025E
15a	Misamis Oriental	Gingoog	Barangay Civoleg, Mt. Lumot, Haribon	1741	8.67870N, 125.028E
15b	Misamis Oriental	Gingoog	Barangay Civoleg, Mt. Lumot, Shrine site	1168	8.70630N, 125.020E
15c	Misamis Oriental	Gingoog	Barangay Lumotan, Sitio San Isidro, Boy Scout Camp, Mt. Balatukan Natural Park	400–2060	8.73255N, 125.003E
15d	Misamis Oriental	Gingoog	Sitio Kibuko-boundary with Barangay Lawaan	420	8.72160N, 125.079E
16	Misamis Oriental	Initao	Initao National Park	8	8.83400N, 123.875E
17	Surigao del Norte	Surigao	Surigao City	22	9.78380N, 125.488E
18	Surigao del Norte	Carrascal	Barangay Adlay	11	9.40868N, 125.896E
19	Surigao del Sur	Hinatuan	Hinatuan	8	8.36666N, 126.333E
20	Surigao del Sur	Lanuza	Barrio Sibahay	152	9.25188N, 126.125E
21	Surigao del Sur	Tandag	Tandag	11	9.10117N, 126.158E

**Sampling Locations.** Data presented here include results of our own surveys (Table 2) and a variety of pre-existing collections, both intensive and incidental, from major Philippine and U.S. Museum collections (see acknowledgements). In addition to extensive collections housed at the California Academy of Sciences (CAS: fieldwork of E. H. Taylor, W. C. Brown, ACA, and colleagues), the University of Kansas (KU) and the National Museum of the Philippines (PNM; field work of RMB, ACD, MBS, KAC, and CDS), we summarize historical records from the Smithsonian (USNM), the Carnegie (CM), and Harvard (MCZ), originating from Misamis Oriental (northern Mindanao Region), Agusan del Norte, Agusan del Sur, Surigao del Norte, Surigao del Sur provinces (Caraga Region), Dinagat, and Siargao records (previously summarized by Ross and Lazell 1990) and Camiguin Province (Camiguin Sur Island). To be as comprehensive as possible in our treatment of northeast Mindanao Island, we include all of these records here, with the caveat that methods of surveying herpetological communities most likely differed among collection efforts and locations.

**Table 2.** The northeast Mindanao herpetological fauna summarized by family, geographical region, current conservation status (IUCN 2010, 2016), and recommended (revised) conservation status (*asterisks indicate conservation status revisions also proposed by Diesmos et al. 2014). Geographical area codes are provided for Agusan del Norte (ADN), Agusan del Sur (ADS), Camiguin Sur (CAM), Dinagat Island (DIN), Siargao Island (records reported by Ross and Lazell, [1990]), Misamis Oriental (MIS), Surigao del Norte (SDN) and Surigao del Sur (SDS) provinces. “H” = historical record only; “N” = new species geographical record; H/N = species known from historical record(s) and additional new locality records, reported here. Additional notes are included (see species accounts for discussion/explanation).

Species			Distribution records by province						Status –> revised	Additional notes
	ADN	ADS	CAM	DIN	SIA	MIS	SDN	SDS		
**AMPHIBIA** (Anurans)										
BUFONIDAE										
*Ansonia muelleri* (Boulenger, 1887)	H/N	N		H		N			VU–>DD*	Priority for taxonomic research; subsequent conservation status assessment needed
*Pelophryne brevipes* (Peters, 1867)		N							LC	
*Rhinella marinus* (Linneaus, 1758)				N	H	N			LC	
Ceratobatrachidae										
Platymantis cf. corrugatus (sp. 34) (Dumeril, 1853)	H/N	N	H/N	H					LC	Priority for taxonomic research
*Platymantis guentheri* (Boulenger, 1884)	H/N	N		H		N				Priority for taxonomic research
Platymantis cf. guntheri sp. 48: Platymantis cf. guentheri (Boulenger, 1884)	N									Priority for taxonomic research
Platymantis cf. guentheri sp. 2						N				Priority for taxonomic research
*Platymantis rabori* Brown, Alcala & Diesmos, 1998	H/N					N			VU	
*Platymantis* sp. 20: “Hilong ground”	N									Priority for taxonomic research
*Platymantis* sp. 21: “Clicker”		N								Priority for taxonomic research
*Platymantis* sp. 38: “Cliff loud”	N	N				N				Priority for taxonomic research
*Platymantis* sp. 39: “Dual”						N				Priority for taxonomic research
Dicroglossidae										
*Fejervarya moodiei* (Taylor, 1920)	H		H				H		DD	Lowland endemic, possibly threatened by invasive species; Conservation status assessment needed
*Fejervarya vittigera* (Wiegmann, 1824)	H	H/N	N	H/N					LC–>DD	Lowland endemic, possibly threatened by invasive species; Conservation status assessment needed
*Limnonectes diuatus* (Brown & Alcala, 1977)	H/N			N		N			VU–>NT	
*Limnonectes leytensis* (Boetger, 1893)	H/N	H/N	H	N		N	H		LC	
Limnonectes cf. magnus (Stejneger, 1910)	H/N	H/N	H/N	H		N		H	NT–>DD	Priority for taxonomic research; subsequent conservation status assessment needed
*Limnonectes parvus* (Taylor, 1920)		N							VU–>NT*	
*Occidozyga laevis* (Günther, 1859)	H/N		H	H		N			LC	
Megophryidae										
*Leptobrachium lumadorum* Brown, Siler, Diesmos & Alcala, 2009	N	N				N			NA–>LC*	New assessment
*Megophrys stejnegeri* (Taylor, 1920)	H/N	H/N		H		N			VU–>NT	
Microhylidae										
*Chaperina fusca* Mocquard, 1892	H	N							LC	
*Kalophrynus sinensis* Peters, 1867	H/N	N	H/N	N	H				LC	
*Kaloula conjuncta meridionalis* Inger, 1954		H/N	N				H		LC–>DD*	Priority for taxonomic research; subsequent conservation status assessment needed
*Kaloula picta* (Duméril & Bibron, 1841)									LC–>DD	Lowland endemic, possibly threatened by invasive species; Conservation status assessment needed
*Kaloula* sp. (undescribed)	N									
Oreophryne cf. nana Brown & Alcala, 1967		N	N/H			N			LC–>DD*	Priority for taxonomic research
Ranidae										
*Pulchrana grandocula* (Taylor, 1920)	H/N	H/N	H/N	H		N		H	LC	
*Sanguirana albotuberculata* (Inger, 1954)	H/N			N					DD–>LC*	
*Staurois natator* (Günther, 1858)	H/N	N		H		N		H	LC	
Rhacophoridae										
Theloderma (Nyctixalus) spinosum (Taylor, 1920)	H/N	N				N			VU–>NT*	
*Philautus acutirostris* (Peters, 1867)	H/N					N			VU–>NT*	
*Philautus poecilius* Brown & Alcala, 1994	H					N			VU	
*Philautus surrufus* Brown & Alcala 1994						N			EN–>VU*	
*Philautus surdus* (Peters, 1863)	H								LC	Priority for taxonomic research (Mindanao populations unstudied)
*Philautus worcesteri* (Stejneger, 1905)	H/N			N					VU–>NT	
*Polypedates leucomystax* (Gravenhorst, 1829)	H/N	H/N	H/N	H		N			LC	Priority for taxonomic research
*Kurixalus appendiculatus* (Günther, 1858)		N	N	H/N		N			LC–>DD*	Priority for taxonomic research; subsequent conservation status assessment needed
*Rhacophorus bimaculatus* (Peters, 1867)	H/N	N		N		N			VU–>NT*	
*Rhacophorus pardalis* (Günther, 1858)		N	H/N		H				LC	
**AMPHIBIA** (Caecilidae)										
Ichthyophiidae										
*Ichthyophis minadanaoensis* (Taylor, 1960)		N							DD	
**REPTILIA** (Lizards)										
Agamidae										
*Bronchocela* sp.	H/N		H/N	H	H	N			NA–>DD	New assessment
*Draco bimaculatus* (Günther, 1864)	H/N	H/N		H	H	N			LC	
*Draco cyanopterus* Peters, 1867	H/N	N	H/N	H/N		N			LC	
*Draco mindanensis* Stejneger, 1908	H	N		H					VU	
*Draco ornatus* (Gray, 1845)	H			H					LC	
Gonocephalus cf. interruptus (Boulenger, 1885)	N	N	H/N	H/N		N			DD	
*Hydrosaurus pustulatus* Escholtz, 1829	N		H/N	H/N	H	N		H	VU	
Dibamidae										
Dibamus cf. leucurus Taylor, 1915			H			N			DD	
Gekkonidae										
*Cyrtodactylus agusanensis* (Taylor, 1915)	H/N	N				N			LC	
*Cyrtodactylus annulatus* (Taylor, 1915)	H/N	H/N	H/N			N	H		LC	
*Cyrtodactylus mamanwa* Welton, Siler, Linkem, Diesmos & Brown, 2010				N					NA–>LC	New assessment
*Gehyra mutilata* (Weigmann, 1834)			H/N			N			LC	
Gekko cf. mindorensis (Taylor, 1919)			N						LC–>DD	Priority for taxonomic research
*Gekko gecko* (Linneus, 1758)			N			N			LC	
*Gekko monarchus* (Shlegel, 1836)			H					H	LC	
*Hemidactylus frenatus* (Duméril & Bibron, 1836)		H	H/N	H/N		N			LC	
*Hemidactylus platyurus* (Schneider, 1792)		H		H				H	LC	
Hemiphyllodactylus cf. typus Bleeker, 1860		N							LC–>DD	Priority for taxonomic research
*Lepidodactylus aureolineatus* Taylor, 1915	H		H						LC	
*Lepidodactylus labialis* (Peters, 1864)	H					N			LC–>DD	
*Pseudogekko pungkaypinit*				H		N			LC	
*Ptychozoon intermedium* Taylor, 1915				H						
Scincidae										
*Brachymeles vulcani* Siler, Jones, Diesmos, Diesmos & Brown, 1912			N						VU	
*Brachymeles tiboliorum* Siler, Jones, Diesmos, Diesmos & Brown, 1912						N			NA–>DD	New assessment
*Brachymeles hilong* Brown & Rabor, 1967	H/N	N	H			N	N	H	NT	
*Brachymeles orientalis* Brown & Rabor, 1967	H/N	H/N	H/N	N		N			LC	
*Emoia atrocostata* (Lesson, 1830)				H						
*Eutropis multicarinata*	H	H/N	H	H	H	N			LC–>DD	Priority for taxonomic research
Eutropis cf. multicarinata		N		N		N			LC–>DD	Priority for taxonomic research
Eutropis cf. indeprensa (Brown & Alcala, 1980)		N	H	N					LC–>DD	Priority for taxonomic research
*Eutropis multifasciata* (Kuhl, 1820)	N	N	H/N	H					LC	
*Lamprolepis smaragdina philippinica* (Merten, 1928)	N	N	H/N	H			H		LC	Priority for taxonomic research
*Lipinia auriculata herrei* (Taylor, 1922)	H								LC	
*Lipinia pulchella pulchella* Gray, 1845	H	N		H					LC	
*Lipinia quadrivittata* (Peters, 1867)		N	H						NA–>DD	New assessment; Priority for taxonomic research
*Otosaurus cumingi* Gray, 1845		N		H					LC	
Parvoscincus cf. kitalangladensis (Brown, 1995)	H/N	N				N		H	LC	
*Parvoscincus steerei* (Stejneger, 1908)	H/N	N	H			N	N		LC	Priority for taxonomic research
*Pinoyscincus abdictus abdictus* (Brown & Alcala, 1980)	H/N	N	H/N	H		N		H	LC	
*Pinoyscincus coxi coxi* (Taylor, 1915)	H/N	H/N	H/N			N		H	LC	
*Pinoyscincus jagori jagori* (Peters, 1864)	N	N		H/N	H				LC	
*Pinoyscincus mindanensis* (Taylor, 1915)	H/N			H			N		NT	Conservation status assessment needed
*Sphenomorphus acutus* (Peters, 1864)	H	N		H					LC	
*Sphenomorphus diwata* Brown & Rabor, 1967	H							H	DD	Conservation status assessment needed
*Sphenomorphus fasciatus* (Gray, 1845)	H/N	H/N	H/N	H		N			LC	
*Sphenomorphus variegatus* (Peters, 1867)	H/N	H/N	H/N	H		N			NA–>LC	New assessment
*Tropidophorus misaminius* Stejneger, 1908	H/N	H/N	H/N	N		N			LC	
*Tropidophorus partelloi* Stejneger, 1910	H/N	N		H					LC	
Varanidae										
*Varanus cumingi* Martin, 1839		N	N	H		N			LC	
**REPTILIA** (Snakes)										
Colubridae										
*Ahaetulla prasina preoccularis* (Taylor, 1912)	H/N			H		N			LC	
*Boiga cynodon* (Boie, 1827)				H					LC	Priority for taxonomic research
*Boiga dendrophila latifasciata* (Boulenger, 1896)				H						
*Calamaria gervaisi* Duméril, Bibron & Duméril, 1854	H/N					N			LC	
*Calamaria lumbricoidea* H. Boie in F. Boie, 1827	H/N	N	H/N	H		N		H	LC	
*Chrysopelea paradisi* Boie, 1827	H			H/N					LC	
*Coelagnathus erythrurus* Duméril, Bibron & Duméril, 1854		N	H/N	H					NA	Priority for taxonomic research
*Cyclocorus nuchalis taylori* Leviton, 1967	H/N		H	N	H				LC	
*Dendrelaphis marenae* Vogel & Van Rooijen, 2008		N			H				LC	
*Dendrelaphis philippinensis* (Günther, 1879)	N		H/N	H	H				NA–>LC	New assessment
*Gonyosoma oxycephalum* (Boie, 1827)		H		H					LC	
*Lycodon capucinus* (Boie, 1827)	N		H/N	N			H		LC	
*Lycodon dumerillii* (Boulenger, 1893)		N		N					LC	
*Oligodon maculatus* (Taylor, 1918)	N							H	LC	
*Stegnotus muelleri* Duméril, Bibron & Duméril, 1854	N	N		N		N			LC	
Natricidae										
*Rhabdophis auriculata auriculata* (Günther, 1858)	H/N	N		H		N			LC	
*Rhabdophis lineatus* (Peters, 1861)	H/N	N		H		N			LC	
*Tropidonophis dendriphiops* (Günther, 1883)		N	H/N	H		N			LC	Priority for taxonomic research
Elapidae										
*Calliophis philippina* (Günther, 1864)	N	N	H/N	H		N			NA–>LC	New assessment
*Calliophis* sp.				N						Priority for taxonomic research
*Naja samarensis* Peters, 1861	H		H/N	H					LC	
*Ophiophagus hannah* (Cantor, 1836)				H						
Homalopsidae										
*Cerberus schneiderii* (Schlegel, 1837)				H						
Hydrophiidae										
*Hydrophis platyurus* Linneaus, 1766							H		LC	
Lamprophiidae										
*Oxyrhabdium modestum* (Dümeril, 1853)	H/N		H/N	H		N			LC	Priority for taxonomic research
*Psammodynastes pulverulentus* (Boie, 1827)	H/N	N	N	H	H	N			NT–>LC	
Pareidae										
*Aplopeltura boa* (Boie, 1827)	N	H/N		N					LC	
PytHonidae										
*Malayopython reticulatus* (Schneider, 1801)	N								NA	Conservation status assessment needed
Typhlopidae										
*Ramphotyphlops braminus* (Daudin, 1803)			H/N						LC	
Ramphotyphlops cf. cumingi (Gray, 1845)	N								DD	Priority for taxonomic research
*Malayotyphlops* sp.	H								LC	Priority for taxonomic research
Viperidae										
Trimeresurus cf. flavomaculatus (Gray, 1842)	N	N		H		N			LC	Priority for taxonomic research
*Tropidolaemus subannulatus* (Gray, 1842)	H/N			N		N			LC	Priority for taxonomic research
*Tropidolaemus philippensis* (Gray, 1842)				N					NA	Priority for taxonomic research
**REPTILIA** (Turtle)										
Bataguridae										
*Coura amboinensis* (Riche in Daudin, 1802)				H					LC	
**REPTILIA** (Crocodile)										
Crocodylidae										
*Crocodylus porosus* Schneider, 1801				H				H	LC	

## Results

We document at least 126 species of amphibians and reptiles from northeast Mindanao and adjacent islands, including 40 species of frogs, one species of caecilian, 49 species of lizards, 35 species of snakes, one species of freshwater turtle, and one species of crocodile (Table 2). This diversity represents approximately 36% percent of the total Philippine herpetofauna (approximately 350 species; Brown 2007; Brown et al. 2008; Diesmos and Brown 2011; Brown and Stuart 2012; Diesmos et al. 2015) and 85–90% of the taxa reported here are endemic to the Philippines.

We provide accounts for each species, provide notes on their habitat and natural history, and draw attention to many unresolved taxonomic problems (involving ~40% of the species included) relevant to particular taxa. We also comment on the conservation status of individual species when our new data suggest that existing conservation status assessments (IUCN 2010, 2016; Diesmos et al. 2014, 2015) are out of date or require revision (Siler et al. 2011a; McLeod et al. 2011; Brown et al. 2012a, 2013a). In cases of taxonomic uncertainty involving frogs of the family Ceratobatrachidae, we refer to undescribed species by informal place-holder nicknames derived for distinctive characteristics (phenotype, habitat, calls; Brown et al. 2012a) and species identification numbers, following Brown et al. (2015)

## Species accounts

### Amphibia

#### Family Bufonidae

##### *Ansonia muelleri* (Boulenger, 1887)

**Figure 2.**
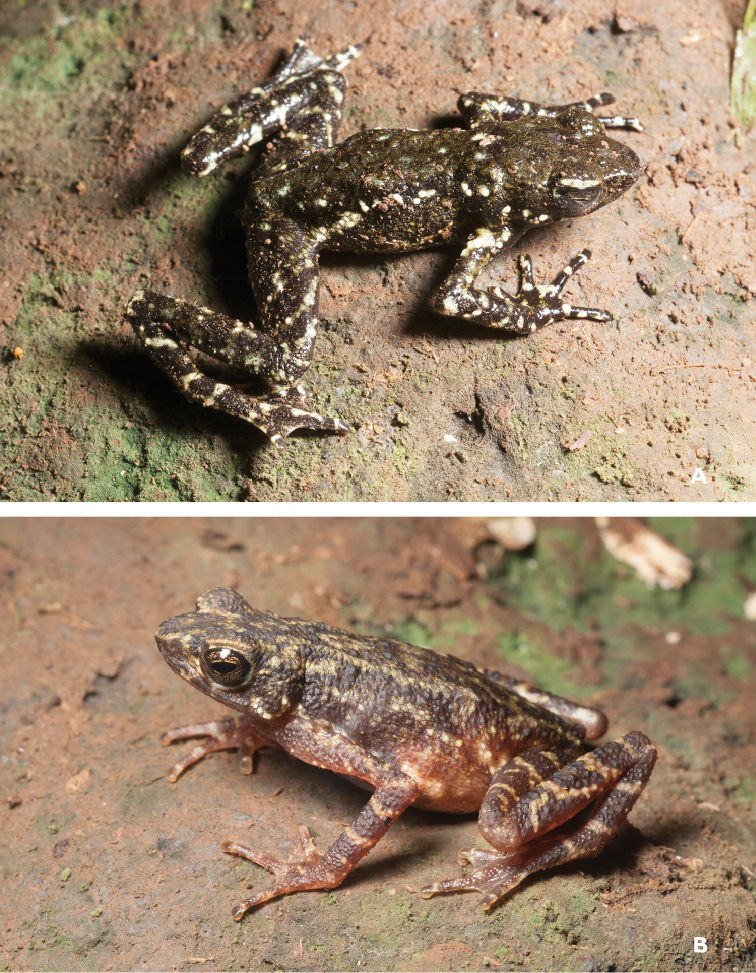
*Ansonia muelleri* male (**A** KU 332971) and female (**B** KU 334106) from Mt. Lumot, Municipality of Gingoog City, Misamis Oriental Province. Photos: RMB.

This species (Fig. 2) is widespread across the island of Mindanao and is the sister species to *Ansonia mcgregori* from the southern tip of the Zamboanga peninsula (Matsui et al. 2010; Sanguila et al. 2011). With its highly specific torrent-dwelling larval habitat requirements (Inger 1960, 1985), this species is limited to montane habitats (and lowlands immediately adjacent to mountains) with high gradient stream flow. As a result, it is widespread but distributed patchily throughout Mindanao in strips of geographic ranges surrounding major mountain ranges (Inger 1954, 1992, 2005; Duellman and Trueb 1994; Matsui et al. 2010). In suitable habitat this species was observed in large numbers and was often the most commonly observed amphibian in an immediate area. Adults were typically collected in splash zones near rapids or waterfalls although they were also found some distance from water, especially after a period of rainfall. A recent phylogeographic study identified five genetically divergent and geographically structured haplotype groups in central and eastern Mindanao. Sanguila et al. (2011) argued that, at a minimum, these lineages should be recognized as evolutionarily significant units (ESUs) for conservation purposes, and that they may represent distinct species. Two of these ESUs are represented in this study and morphological differences were observed between one form found at Mts. Hilong-hilong and Magdiwata and another morphotype present at Mts. Balatukan and Lumot. Little can be made of this species current conservation status (“Vulnerable:” B1ab(iii); IUCN 2016) because no actual studies of its population status have ever confirmed the “populations trend decreasing” assessment and because its “severely fragmented” distribution appears to be the result of its natural, larval habitat requirements. However, if Sanguila et al. (2011) data can be interpreted as evidence for multiple cryptic species contained within *Ansonia muelleri*, then each of these five divergent lineages would have much more limited distributions, perhaps qualifying each for some level of threat category. In the absence of firm conclusions either way, and until taxonomic studies are undertaken to confirm or refute this possibility, *Ansonia muelleri* should be considered “Data Deficient” (DD; IUCN 2010; Diesmos et al. 2014).

Sites and specimens: AN 10: KU 332918. AN 11: KU 332862–901, KU 334101–03; AN 12: KU 332917; AN 14: KU 332902–16; AN 5: USNM 305558–68, USNM 305562–63, 305569–70; AN 9: KU 323441–44; AS 5: KU 319526, KU 319548–55, KU 319710, KU 319527–47, KU 319556, KU 319522–25; D 5: KU 309947–68; MO 2: KU 334802, KU 334105–06, KU 332958–71, KU 332927–57; MO 3: KU 332919–26, KU 334104; MO 5: KU 319711, KU 319713, KU 319714-319715, KU 319718, KU 319723–24, KU 319726, KU 319728–9, KU 319732, KU 319734–35, KU 319740, KU 319747, KU 319749–50, KU 319752, KU 319759–61, KU 319719, KU 319721, KU 319727, KU 319730, KU 319739, KU 319741–43, KU 319746, KU 319748, KU 319751, KU 319753, KU 319754, KU 319756, KU 319716, KU 319725, KU 319755, KU 319757, KU 319712, KU 319744, KU 319717, KU 319720, KU 319733, KU 319736-38, KU 319745, KU 319758, KU 319762, KU 319722, KU 319731; AN 3: CAS 133377–80, CAS 133387–90, CAS 133405–06, CAS 133402–03, CAS 133443–55, CAS 137509–15, CAS 133489–93; AN 4: CAS 133216–29, CAS 133153–61, CAS 133182–98, CAS 133249, CAS 133344, CAS 133283–5; AN 5: CAS 248315–9; AN 6: CAS 185731, CAS 133520, CAS 133528–29, CAS 133545–46, CAS 133535–39.

##### *Pelophryne brevipes* (Peters, 1867)

**Figure 3.**
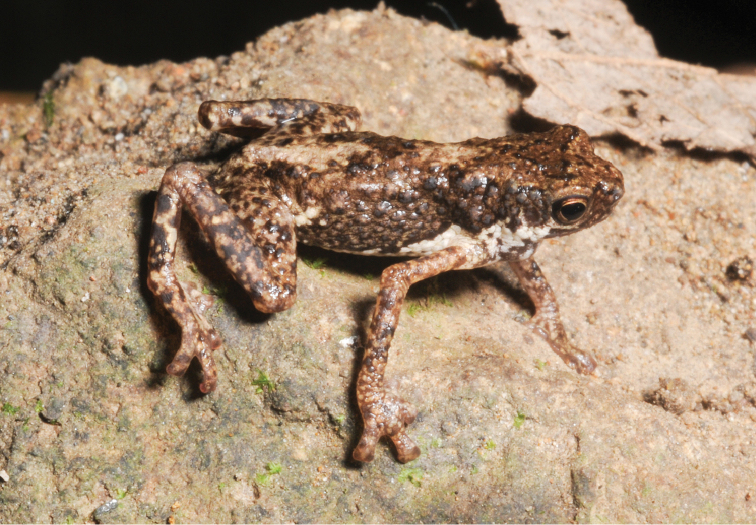
*Pelophryne brevipes* male (KU 334658) from 1200 masl, Shrine Site, Municipality of Gingoog, Mt. Lumot, Misamis Oriental Province. Photo: RMB.

As currently understood, this species (Fig. 3) inhabits an improbably disjunct distribution that includes Mindanao and Basilan Islands (type locality: Zamboanga), and parts of the Malay Peninsula, Sumatra, Mentawi Islands and Natuna Islands (Inger 1954, 1999). Considered “Least Concern” (LC) by IUCN (2016), this uncommon and patchily distributed small species can be found calling (with soft “peeping” vocalization) on the surfaces of shrubs and understory trees in the vicinity of running water. It appears to tolerate moderate levels of forest disturbance and has been found on ornamental plants around buildings on forest edges (RMB *personal observations*). We suspect that this species eventually will be recognized as a Philippines endemic, and that populations outside of the country will be referred to other species. At that time, reconsideration of its conservation status will be required.

Sites and specimens: AS 5: KU 319434–39.

##### *Rhinella marina* (Linnaeus, 1758)

We frequently observe this common introduced species in the vicinity of human habitations and in agricultural areas on Mindanao. It may have originally been introduced to the Philippines during the industrial revolution and the major sugar cane agricultural production boom on the central Philippine island of Negros (Alcala 1957; Brown and Alcala 1970), and has now become widespread throughout the country (Alcala and Brown 1998; Diesmos et al. 2006, 2015). We observed this species around human populations at most sites, but did not encounter it in forested areas. We collected a single specimen at low elevation on Mt. Lumot.

Sites and specimens: MO 6: KU 333803.

#### Family Ceratobatrachidae

##### *Platymantis* sp. 34: Platymantis cf. corrugatus (Duméril, 1853)

**Figure 4.**
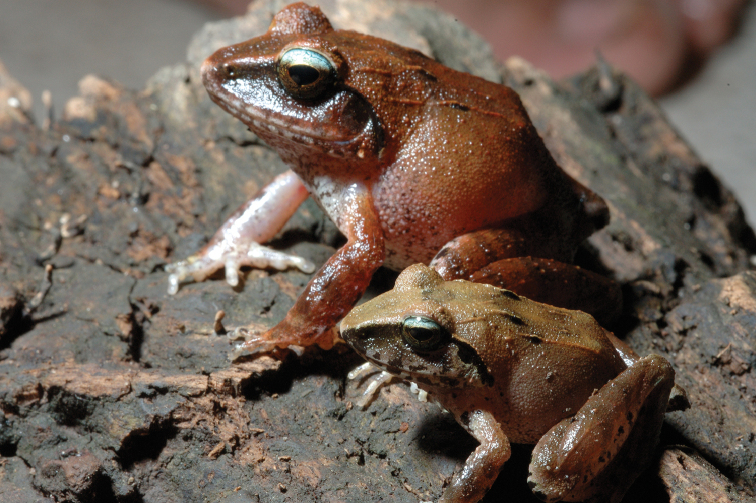
Platymantis cf. corrugatus male (KU 304501) and female (KU 304502) photographed together at Municipality of Mambajao, Mt. Mambajao, Camiguin Sur Island. Photo: CDS.

Platymantis cf. corrugatus (Fig. 4) is a commonly encountered widespread species on the Mindanao PAIC. Populations on these islands are morphologically and acoustically distinguishable from Luzon and Visayan PAIC lineages and molecular studies are underway to determine if these slight differences could constitute the basis for taxonomic recognition. Although the call on all three PAICs is similar, the lineage on the Mindanao PAIC (“sp. 34;” Brown et al. 2015) have a different preferred calling habit (calling from exposed areas, versus under leaf litter on Luzon) and daily pattern of activity (calling all evening versus strictly crepuscular; RMB *personal observations*.). *Platymantis corrugatus* is classified by the IUCN as “Least Concern” (LC; IUCN 2016).

Sites and specimens: AN 1: CAS 133690; AN 11: KU 333301–09; AN 3: CAS 133572, CAS 133622, CAS 133678, CAS 133679–80, CAS 133779, CAS 185732–33; AN 5: USNM 305583–88, KU 319557–73; AS 6: KU 314062–64; C 1: CAS-SUA 24060, CAS-SUA 24106, CAS-SUA 24110; C 10: KU 300351, KU 300355; C 13: KU 309728–67, KU 310353–54; C 14: KU 309768–70; C 2: CAS-SUA 24058, CAS-SUA 2406–12, CAS-SUA 24092, CAS-SUA 24107–08; C 3: CAS-SUA 24063; C 6: CAS-SUA 23024–30, CAS-SUA 23044; C 7: CAS 139038–39, CAS-SUA 23031–42, USNM 305727–28; C 8: CAS-SUA 23043; D 5: KU 310004–06.

##### *Platymantis guentheri* (Boulenger, 1884)

**Figure 5.**
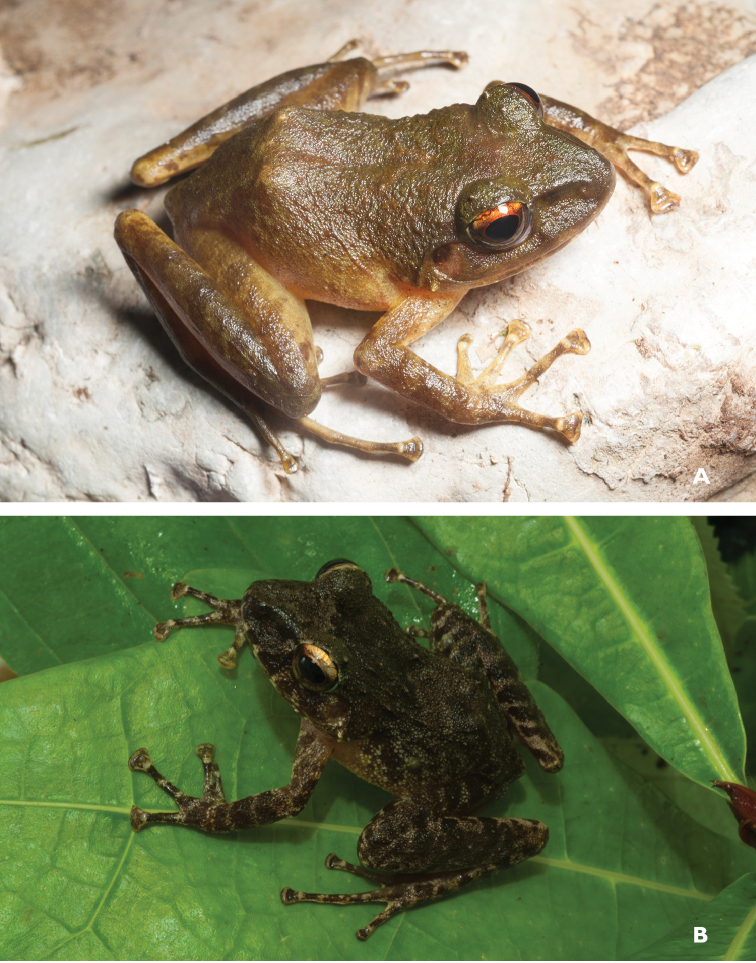
*Platymantis guentheri* male (**A** KU 309208) from Eye Falls, Mt. Hilong-hilong, Municipality of Remedios T. Romualdez, Agusan del Norte Province. Photo: RMB; (**B** KU 333494) from Mt. Mambajao, Municipality of Mambajao, Camiguin Sur Island. Photo: CDS.

As currently understood, this common, widespread species (Fig. 5) is now known from seven Philippine islands throughout the Mindanao PAIC and is known from at least six of Mindanao’s major mountain ranges (AmphibiaWeb 2013); Although *Platymantis guentheri* has been considered “Vulnerable” (VU: B1ab(iii); IUCN 2016) on the basis if an “extent of occurrence less than 20,000 square km’” and distribution “extremely fragmented distribution” with populations trends inferred to be declining on the basis of “continuing decline in the extent and quality of its habitat” (IUCN 2016), continued treatment of this species as under threat of extinction is no longer tractable. Our now extensive surveys throughout Bohol, Samar, Leyte, Dinagat and Mindanao indicate that this very widespread species persists in second growth, degraded, and fragmented habitat patches and is be highly abundant and commonly encountered (and easily identified on the basis of its frequency sweep advertisement call), given sufficient precipitation and any form of vegetation present. We therefore downgrade this species to “Near Threatened” (NT; IUCN 2010; Diesmos et al. 2014) and emphasize that we do not anticipate circumstances that could result in re-elevating of this species to a higher threat category in the foreseeable future.

Sites and specimens: AN 11: KU 333492–526; AN 12: KU 333528–46; AN 5: USNM 305589–93, KU 319609–26; D 4: KU 306320–5, KU 310009; D 5: KU 310007–08, MO 2: KU 333547, MO 5: KU 321843–44, MO 6: KU 333548–50; AN 1: CAS 146460, AN 3: CAS 133411, CAS 133470, CAS 133570–71, CAS 133573, CAS 133644–45, CAS 133651–3, CAS 133780–81, CAS 146469–70, CAS 186122, CAS 186124–26, CAS 196378–79, CAS 200177; AN 4: CAS 133148–49, CAS 133202, CAS 133213, CAS 133257, CAS 133287–88, CAS 133307, CAS 133313, CAS 133332–33, CAS 133357–58; AN 6: CAS 133518–19, CAS 133531, CAS 133547–48, CAS 146468, CAS 186123.

##### *Platymantis* sp. 48: Platymantis cf. guentheri (Boulenger, 1884)

**Figure 6.**
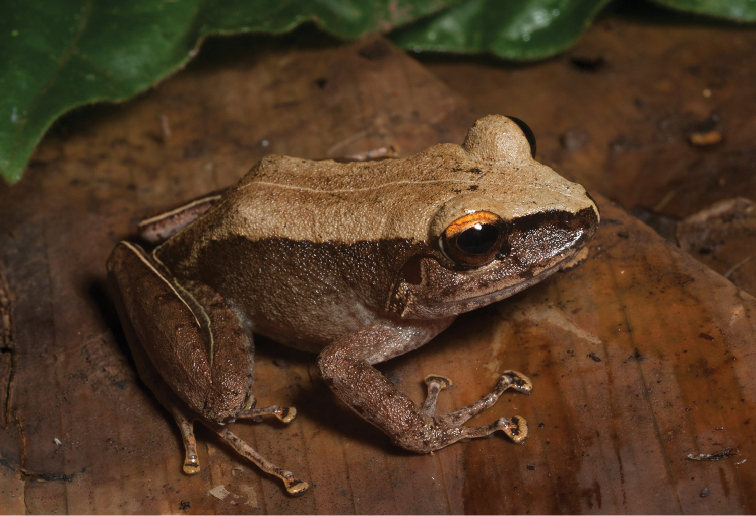
Platymantis cf. guentheri sp. male 1 (KU 333536) from May-Impit, Mt. Hilong-hilong, Municipality of Remedios T. Romualdez, Agusan del Norte Province. Photo: RMB.

We collected four specimens of what appears to be a morphologically distinctive arboreal *Platymantis* (Fig. 6) at our mid- (470 m) and higher (1130 m) elevation sites on Mt. Hilong-hilong. Molecular studies are underway to determine whether these distinctive specimens are a unique species.

Sites and specimens: AN 12: KU 333827; AN 14: KU 333824–26.

##### Platymantis cf. guentheri sp. 2

We collected a single specimen of a morphologically distinctive arboreal *Platymantis* and high elevation on Mt. Lumot. Molecular studies are under way to determine the genetic affinities of the single specimen.

Site and specimens: MO 2: KU 334329.

##### *Platymantis rabori* Brown, Alcala & Diesmos, 1998

**Figure 7.**
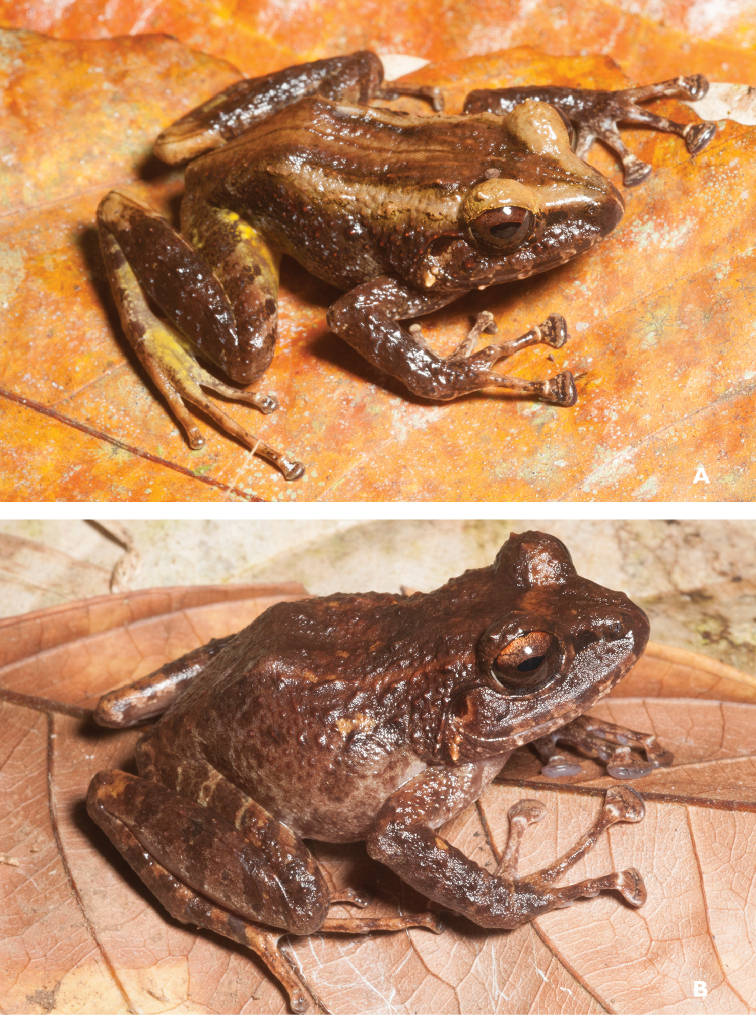
*Platymantis rabori* male (**A** KU 334334) and female (**B** KU 334335) from 900 masl, Mt. Lumot, Gingoog, Misamis Oriental Province. Photos: RMB.

Considered “Vulnerable” (VU; B1ab (iii); IUCN 2016) for the same reasons as *Platymantis guentheri* (above), *Platymantis rabori* (Fig. 7) is relatively widespread on Bohol, Leyte, Samar, and Mindanao (AmphibiaWeb 2013), but is much less commonly encountered and locally considerably less abundant. This species is easily identified by its distinctive morphology (relatively large body size, widely expanded terminal disks of the fingers) and slowly repeated pulsed call, similar to other species of the *Platymantis guentheri* complex (Brown et al. 1997) and it usually calls high in the forest canopy, which may explain why it is seldom collected and rare in collections. This species does appear dependent on forest canopy, suggesting that the original assessment of this species as VU is still appropriate.

Sites and specimens: AN 12: KU 333527, KU 334331–32; AN 13: KU 334333; MO 6: KU 334334–35, AN 5: CAS 197880.

##### *Platymantis* sp. 20: “Hilong ground”

**Figure 8.**
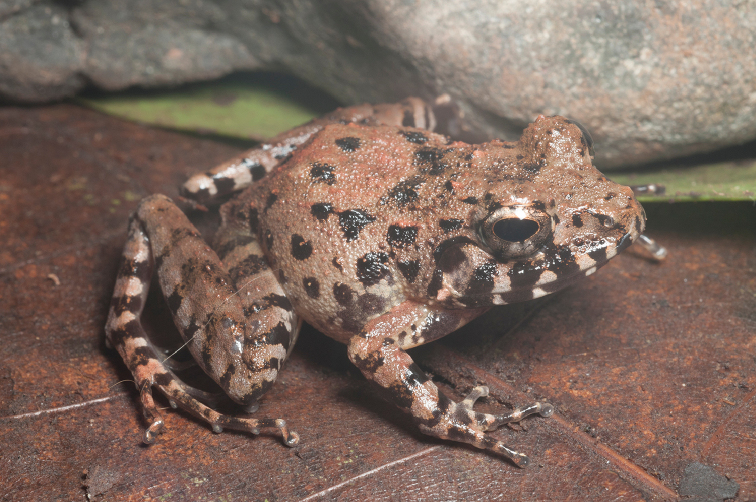
*Platymantis* sp. “Hilong ground” male (KU 334339) from Eye Falls, Mt. Hilong-hilong, Municipality of Remedios T. Romualdez, Agusan del Norte Province. Photo: RMB.

Several specimens of a morphologically distinctive terrestrial (leaf litter) species of *Platymantis* were collected on Mt. Hilong-hilong (Fig. 8). Although we have never heard this species vocalize (and have not yet identified its phylogenetic affinities), it is clearly distinct (intermediate body size and unique coloration) from other species included at this site.

Sites and specimens: AN 11: KU 334336–38; AN 14: KU 334339.

##### *Platymantis* sp. 21: “Clicker”

**Figure 9.**
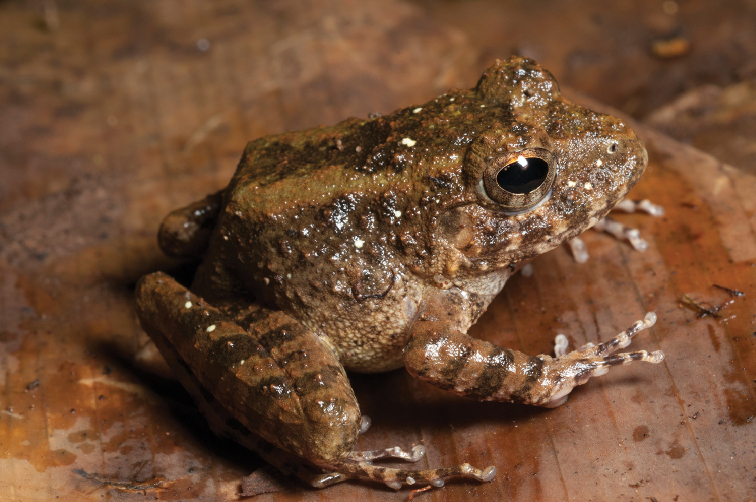
*Platymantis* sp. “Clicker” male (KU 334508) a species recorded but uncollected at 300 masl, Eye Falls, Mt. Hilong-hilong, Municipality of Remedios T. Romualdez, Agusan del Norte Province, Mindanao Island. This photo (by RMB) was taken at Pasonanca Natural Park, Zamboanga City.

This suspected new species of *Platymantis* (Fig. 9) has now been recorded at numerous sites on Mindanao, Bohol, Samar and Leyte islands and is readily identified by its small body size and unique advertisement call, consisting of a long chain of clicking pulses. Preliminary phylogenetic analyses of DNA sequence data suggest that these populations constitute a distinct lineage that is divergent from other Mindanao PAIC species.

Sites and specimens: AS 5: KU 319508–12, KU 319403–17.

##### *Platymantis* sp. 38: “Cliff loud”

**Figure 10.**
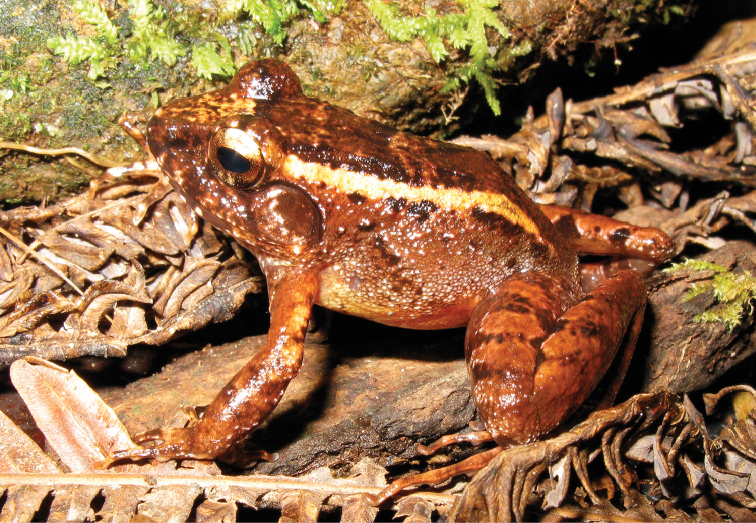
*Platymantis* sp. “Cliff-loud” male (specimen not collected) from Mt. Balatukan, Municipality of Gingoog, Misamis Oriental Province. Photo: ACD.

We collected this morphologically and acoustically distinctive undescribed species of *Platymantis* (Fig. 10) calling from steep hillsides and ravines on Mts. Hilong-hilong, Balatukan, Lumot, and Magdiwata. This new species calls only from the steep slopes and calls with a loud, paired, two-note calls.

Sites and specimens: AN 12: KU 334340–52, KU 334357–62; AN 14: KU 334353–56; AS 5: KU 319470–99, KU 319513–17, KU 319709, KU 319972; MO 6: KU 334363–83.

##### *Platymantis* sp. 39: “Dual”

**Figure 11.**
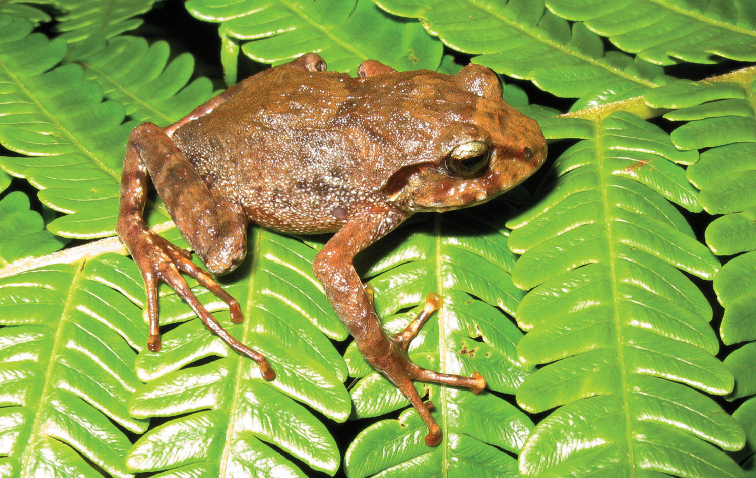
*Platymantis* sp. “Dual” male (specimen deposited in PNM) from Mt. Balatukan, Municipality of Gingoog, Misamis Oriental Province. Photo: ACD.

Two specimens of an acoustically unique *Platymantis* species (Fig. 11) were collected by ACD, MBS and party on Mt. Balatukan. The advertisement call of this population is so distinct that we cannot ally it with any known *Platymantis*.

Sites and specimens: MO 5: KU 319431–32.

#### Family Dicroglossidae

##### *Fejervarya moodiei* (Taylor, 1920)

This endemic species, formerly considered conspecific with *Fejervarya cancrivora* but now afforded the status of an endemic Philippine species, due to its genetic distinctiveness (Kurniawan et al. 2010, 2011), is common in coastal areas of NE Mindanao, brackish water swamps and mangroves, and river mouth estuarine areas. CAS specimens were collected in coastal areas, along riverbanks, four decades ago; an assessment of this species status in these heavily populated areas would be advisable. This species’ conservation status is “Data Deficient” (DD; IUCN 2016). As lowland and coastal habitats throughout the country continue to be invaded by the introduced species *Hoplobatrachus rugulosus* and *Kaloula pulchra* (Diesmos et al. 2006, 2014, 2015), these voracious generalist species replace entire populations of native frogs (RMB, ACD *personal observation*), either via competition for resources or direct predation on native taxa. We suspect this widespread Philippine endemic may soon become threatened with extinction; accordingly we recommend field-based surveys targeting conservation status assessment.

Sites and specimens: AN 8: CAS-SUA 16486–87; C 4: USNM 43210; SN 1: CAS-SUA 16484–85; AN 1: CAS 133726–29, CAS 146463. C 6: CAS-SUA 23092–94.

##### *Fejervarya vittigera* (Wiegmann, 1824)

**Figure 12.**
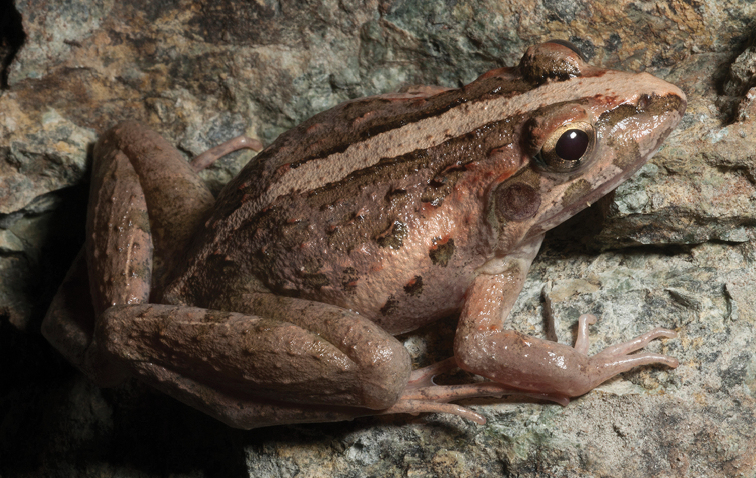
*Fejervarya vittigera* has been observed in coastal and inland low elevation areas throughout northeast Mindanao. Photo (RMB; deposited at KU; specimen not yet cataloged) from the Municipality of Burauen, Leyte Island.

The first endemic Philippine species known to science, *Fejervarya vittigera* (Fig. 12) inhabits low elevation aquatic habitats and is often found in streams, drainage ditches and flooded rice fields. It is easily identified at a distance by its loud “honking” advertisement call and aggregation in large choruses. This species conservation status is currently “Least Concern” (LC; IUCN 2016) but we revise it to “Data Deficient” (DD; IUCN 2010 to reflect the paucity of available data on its actual confirmed (versus presumed; IUCN 2016) distribution and the degree to which it may be threatened by invasive species. Like *Fejervarya moodiei*, this is another species that is quickly replaced when *Hoplobatrachus rugulosus* and *Kaloula pulchra* invade its low elevation riparian habitat (RMB, ACD *personal observation*; Diesmos et al. 2006, 2014, 2015).

Sites and specimens: AN 1: CAS 185728–30; AN 1: CAS 133726–29, CAS 146463; AN 8: CAS-SUA 16486–87; AS 2: USNM 229333–54; AS 4: KU 314051–53; C 4: USNM 43210; C 6: CAS-SUA 23092–94; C 12: KU 302048–50; D 1: USNM 229311–13; D 4: KU 305655–57; SN 1: CAS-SUA 16484–85.

##### *Limnonectes diuatus* (Brown & Alcala, 1977)

**Figure 13.**
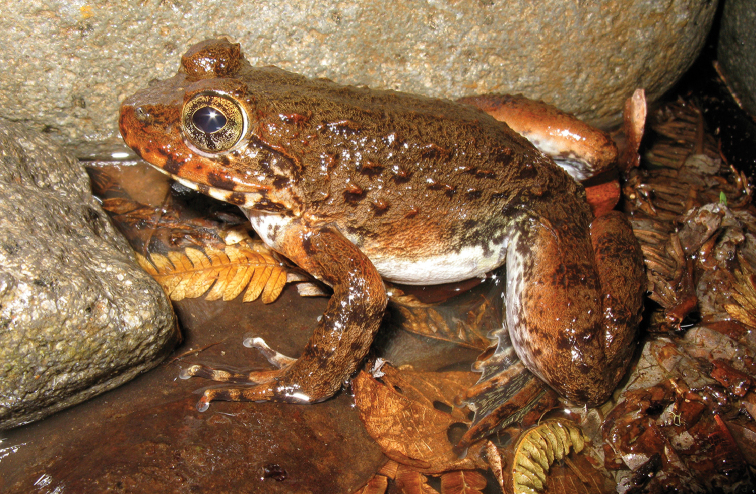
*Limnonectes diautus* male (KU 320112) from 1900 masl, Mt. Balatukan, Municipality of Gingoog, Misamis Oriental Province. Photo: ACD.

Recent collections of this species (Fig. 13) at high elevations on Mts. Balatukan and Lumot and on Dinagat Island make it clear that, as presently understood, this species is considerably more widespread than previously thought. Previously recorded in the Diwata range (Brown and Alcala 1977) and Mt. Kitanglad (Bukidnon Province; based on a single, tentatively identified specimen in FMNH; ACD *personal observation*), this species is most likely widely distributed on montane formations throughout much of northern and central Mindanao. This species’ conservation status has been arbitrarily listed since 2004 as “Vulnerable” (IUCN 2016) based solely on its previously assessed range of less than 20,000 square kilometers and possessing a “severely fragmented” distribution with “continuing decline in the extent and quality of its forest habitat,” (IUCN 2016) none of which has actually been determined with accompanying field based data. Now that is it known to inhabit a much wider geographic range (data presented here), we adopt the suggested revision of Diesmos et al. (2014) who downgraded this species status to “Near Threatened” (NT: IUCN 2010). Given that there have, in fact, been no actual field studies of habitat fragmentation in its actual range and that we know nothing of the extent or quality of its required habitat, we fail to see how this species’ status could be elevated to a higher level, unless drastic land use changes at high elevations were to occur. Many of the known areas of occurrence are now protected areas (Mts. Hilong-hilong, Kitanglad) or proposed protected areas (e.g., Mt. Lumot), so this species may well be reasonably well protected for the foreseeable future.

Sites and specimens: AN 12: KU 33339–43; AN 13: KU 333369–73; AN 14: KU 333375. KU 333381–89; AN 3: CAS 133430–32, CAS 133434, CAS 133500, CAS 139389–93; D 5: KU 309992–310000.

##### *Limnonectes leytensis* (Boetger, 1893)

**Figure 14.**
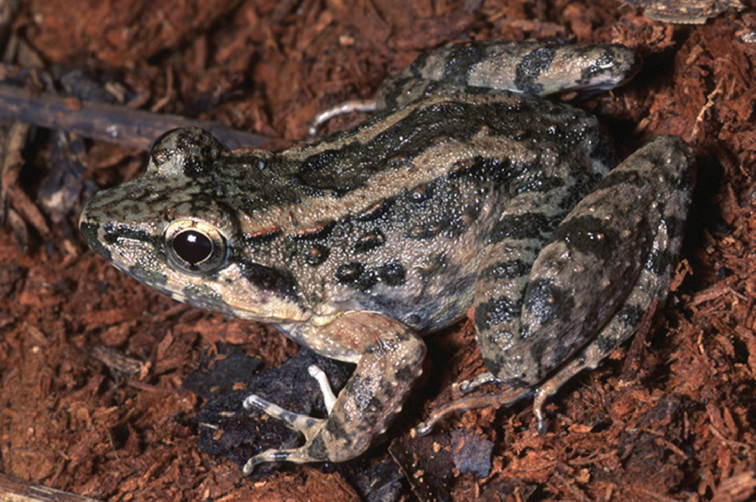
*Limnonectes leytensis* has been collected in Bunawan, Agusan del Sur Province and on Dinagat Island. Photo: RMB (from Bohol; KU 326352).

The Leyte Swamp Frog, *Limnonectes leytensis* (Fig. 14), is widely distributed on the Mindanao, Visayan, and Romblon PAICs and is frequently observed in swamps or marshes, but also along small streams or other bodies of water. We collected specimens on grassy banks of streams on Dinagat Island, and in marshy areas of Bunawan. Its conservation status is “Least Concern” (LC; IUCN 2016).

Sites and specimens: AN 10: KU 333397–98, KU 333807; AS 2: USNM 229332; AS 4: KU 314059–61; D 2: KU 306071, KU 306073; D 4: KU 306064, KU 306066–67, KU 306074, KU 306078–80; MO 5: KU 319892–98; MO 6: KU 333809–14; AN 1: CAS 133738–9; AN 3: CAS 133369, CAS 133383, CAS 133681–82, CAS 145939; C 1: CAS-SUA 24087–88; C 2: CAS-SUA 24090, CAS-SUA 24109; C 3: CAS-SUA 24091; C 6: CAS-SUA 22857–59, CAS-SUA 22863–68, CAS-SUA 23084–91; SN 1: CAS 60580–82.

##### Limnonectes cf. magnus (Stejneger, 1910)

**Figure 15.**
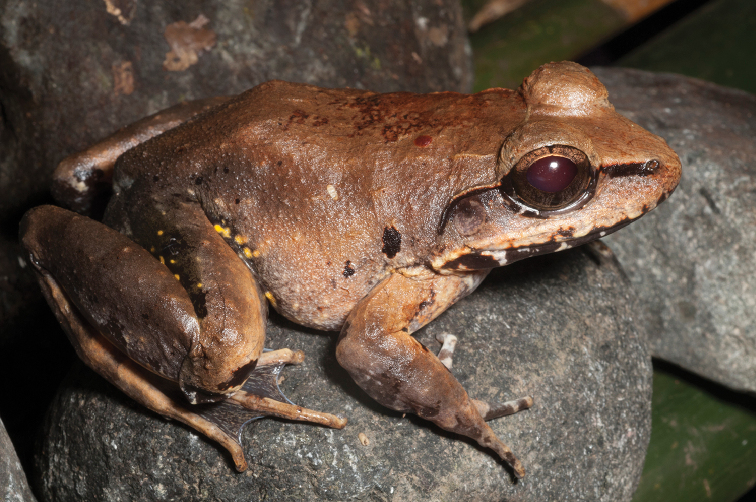
Limnonectes cf. magnus female (KU 333351) from Eye Falls, Mt. Hilong-hilong, Municipality of Remedios T. Romualdez, Agusan del Norte Province. Photo: RMB.

Stejneger (1910) described *Limnonectes magnus* from specimens collected at high elevation on Mt. Apo. Over the century, this name was widely applied to all large bodied fanged frogs of the Mindanao PAIC (Samar, Leyte, Bohol; Brown and Alcala 1970; Alcala and Brown 1998). Evans et al. (2003) demonstrated the genetic distinctiveness of the high elevation Mt. Apo species, which is considerably divergent from the widespread low elevation species. Thus, the taxonomic status of the widespread, low elevation species remains unresolved with respect to real *Limnonectes magnus* at its type locality. *Limnonectes magnus* has been treated as “Near Threatened” because the species is hunted for its meat. However, the degree to which this species actually is “over-harvested” has never been properly ascertained (contrary to IUCN 2016) and there have been no studies whatsoever of its status on Mt. Apo. It remains possible that the widespread low elevation form (Fig. 15) should be downgraded to “Least Concern,” whereas the high elevation Mt. Apo populations may actually be range-restricted and worthy of higher conservation status. Until taxonomic studies have properly been undertaken, surveys for genetic variation are conducted throughout its range, and populations at high elevations have been studied, we argue that this species should be treated as “Data Deficient” (DD; IUCN 2016) and that field-base conservation efforts be focused on the Mt. Apo population.

Sites and specimens: AN 10: KU 333396, KU 333399–415, KU 333808. AN 11: KU 333343–68, KU 334798; AN 12: KU 333390–91, KU 333394–95, KU 334107; AN 14: KU 333374, KU 333376–80, KU 333382, AN 3: CAS 133384–86, CAS 133429, CAS 133433, CAS 133554, CAS 133673–74, CAS 133792, CAS 139396, CAS 186128; AN 4: CAS 133203–06; CAS 133232–3; AN 5: USNM 305598–99; AS 2: USNM 229355–58; AS 5: KU 319383–99; C 12: KU 302139–40; C 13: KU 309685–706; C 14: KU 309707–27; C 6: USNM 305729; C 7: USNM 305730–32; D 2: KU 306003–84; D 4: KU 306062–81; D 5: KU 309974–91; MO 2: KU 333428–77, KU 333478, KU 334796–97, KU 334799; MO 4: KU 333417–27; MO 5: KU 320078–116; MO 6: KU 333479–91; SS 2: CAS-SUA 17462.

##### *Limnonectes parvus* (Taylor, 1920)

**Figure 16.**
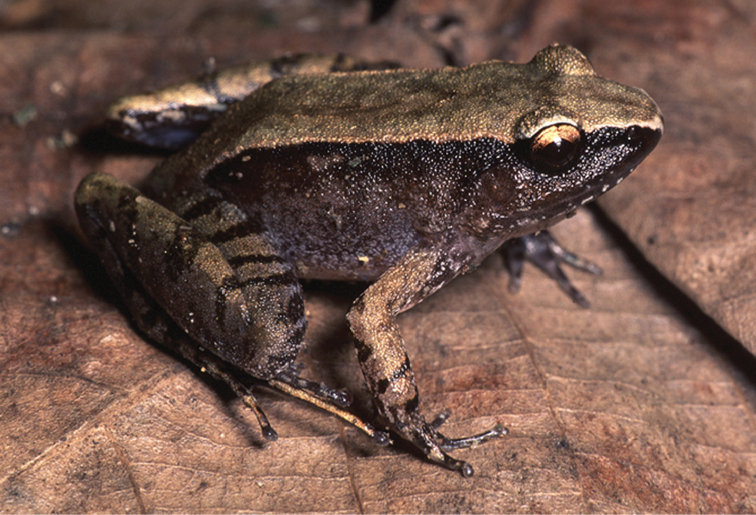
*Limnonectes parvus* has been collected in Municipality of Bunawan, Agusan del Sur Province. (from Davao City Province; KU 326069). Photo: RMB.

Commonly encountered and locally abundant in central, southern, and western Mindanao, this species (Fig. 16) is conspicuously absent at most NE Mindanao localities and has not been recorded on Bohol, Leyte, Dinagat, Siargao, or Samar. Considered “Vulnerable” (VU; IUCN 2016: B1ab (iii) because of is supposedly small (< 20,000 square kilometers), its “severely fragmented distribution” and “continued decline in quality and extent of Mindanao’s forests, this species is now known from Bunawan in NE Mindano (the origin of our specimens), throughout much of south-central Mindanao, and all the way west to Zamboanga and Basilan. Given that no studies of its distribution have ever actually been conducted, it appears widespread, commonly encountered, and locally abundant wherever present, we consider a revision of its conservation status warranted. We take issue with the IUCN (2016) characterization of is range as “severely fragmented” and we follow Diesmos et al. (2014) in their downgrading of this species to “Near Threatened” (NT) using IUCN status assessment criteria (IUCN 2010). Our acceptance of this proposal (a downgrade from VU to NT, and not Least Concern) is based on the observation that the species appears somewhat dependent upon vegetation cover (but second growth and nursery forests appear sufficient to sustain large populations; RMB, ACD, MBS *personal observation*).

Sites and specimens: AS 5: KU 319518–21.

##### *Occidozyga laevis* (Günther, 1859)

Yellow-bellied Puddle Frogs are widespread, aquatic, non-endemic species found in a wide variety of freshwater habitats from streams, rivers, swamps, and flooded rice fields at low elevation, to cascading mountain streams in montane environments. This species’ conservation status is “Least Concern” (LC; IUCN 2016).

Sites and specimens: AN 10: KU 333097–102; AN 5: USNM 305581–82, USNM 305769–70; C 6: CAS-SUA 22854; D 5: KU 310001–03; MO 2: KU 333109–37; MO 3: KU 333103–08; MO 5: KU 319790–98; MO 6: KU 333138–47; AN 3: CAS 133637–42, CAS 133675–77, CAS 137519.

#### Family Megophryidae

##### *Leptobrachium lumadorum* Brown, Siler, Diesmos & Alcala, 2009

**Figure 17.**
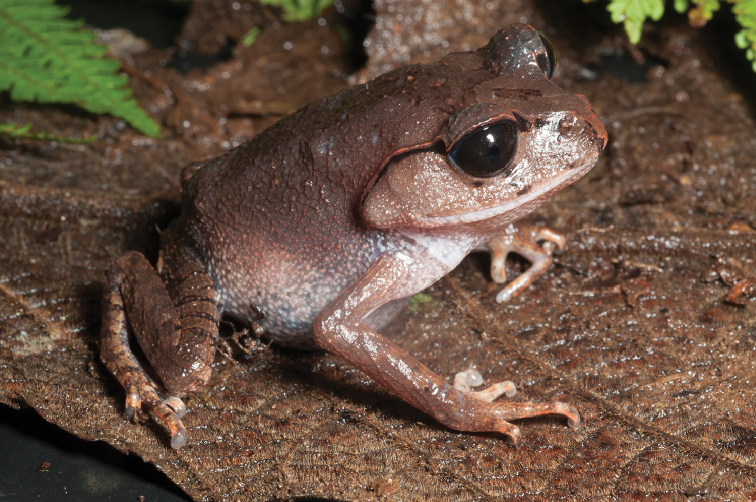
*Leptobrachium lumadorum* male (KU 334801) from 1200 masl, Shrine Site, Mt. Lumot, Municipality of Gingoog, Misamis Oriental Province. Photo: RMB.

Widespread throughout Mindanao and Basilan, but not Dinagat, Siargao, Leyte, Samar or Bohol (Brown et al. 2009), this species (Fig. 17) occurs at naturally low abundances in a wide variety of habitats provided that some vegetation persists. We have encountered this species in agricultural areas adjacent to second growth, tree nurseries, along riparian habitats in lowland forest, and up to 1,500 m on Mts. Magdiwata, Hilong-hilong, Balatukan, and Lumot. Previously unassessed, we categorize this species as “Least Concern” (LC) using IUCN conservation status assessment criteria (IUCN 2010).

Sites and specimens: AN 11: KU 333673–75; AN 12: KU 333676; AS 5: KU 319449–50; MO 2: KU 333677–79, KU 334801; MO 5: KU 319773–76; MO 6: KU 333680–85.

##### *Megophrys stejnegeri* (Taylor, 1920)

**Figure 18.**
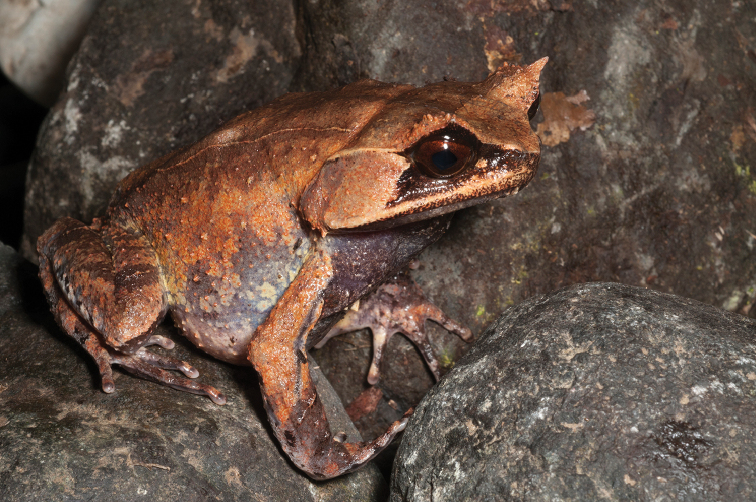
*Megophrys stejnegeri* male (KU 333289) from Mt. Lumot, Municipality of Gingoog, Misamis Oriental Province. Photo: RMB.

This species conservation status has been listed as “Vulnerable” (VU; IUCN 2016). Since 2004, this classification is no longer tenable given new information on its extremely widespread distribution (throughout all islands of the Mindanao PAIC), its wide ecological tolerance of disturbance, and the fact that it is commonly encountered and locally abundant. For the same reasons we have suggested downgrading *Platymantis güntheri*, *Limnonectes parvus* and *Limnonectes diuatus*, we similarly propose a downgrade of *Megophrys stejnegeri* (Fig. 18) to “Near Threatened” (NT; IUCN 2010) and emphasize that we can foresee no circumstances that conceivably would result in a higher level of threat category being assigned to this species in the near future.

Sites and specimens: AN 10: KU 333286; AN 11: KU 333259–72; AN 12: KU 333274–76; AN 13: KU 333277–78, AN 14: KU 333279–85; AN 3: CAS 133266, CAS 133391–92, CAS 133409–10, CAS 133465–68, CAS 133474–76, CAS 133486–88, CAS 133636, CAS 133657, CAS 133782, CAS 200178; AN 4: CAS 133127–32, CAS 133181, CAS 133250, CAS 133302–04, CAS 133310, CAS 133324, CAS 133337, CAS 133345–52; AN 5: USNM 305571–80; AN 6: CAS 133516–17, CAS 133527, CAS 133549–51; AS 2: USNM 229330–1; AS 5: KU 319592–608; D 5: KU 310029–31; MO 2: KU 333290–98, KU 334793, KU 334800; MO 3: KU 333287–9, KU 334792, MO 5: KU 319763–72, KU 321851; MO 6: KU 333299–300, KU 334794.

#### Family Microhylidae

##### *Chaperina fusca* Mocquard, 1892

**Figure 19.**
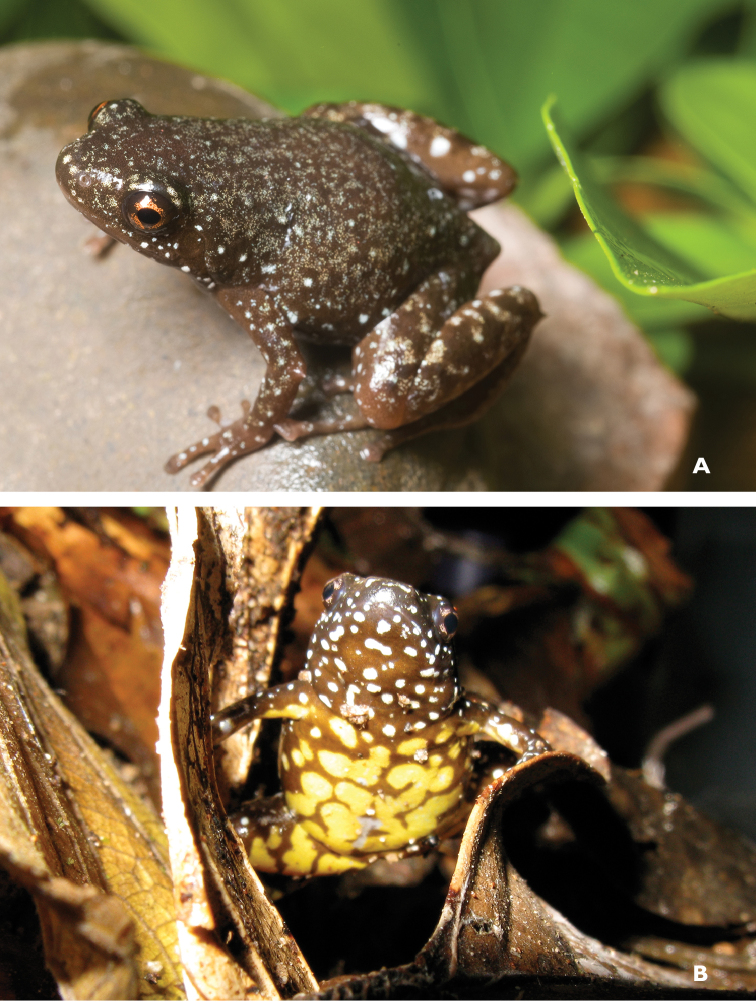
*Chaperina fusca* female (**A** KU 321410) from Barangay Tulosa, Pasonanca Natural Park, Zamboanga City Province, Mindanao Island. Photo: RMB; (**B** KU 319441.) male from 1900 masl, Mt. Balatukan, Municipality of Gingoog, Misamis Oriental Province. Photo: ACD.

Known only from Mindanao, Jolo, and Palawan in the Philippines, but also from Peninsular Malaysia, Thailand and central Borneo, this species (Fig. 19) inhabits a wide geographic range, but is characterized by a patchy and unpredictable distribution. We commonly find this species in moderately sized (10–25 individuals), tightly clustered choruses surrounding stream-side pools (preferred breeding habitat) in rock impressions. The advertisement call of this species is a series of high frequency, quiet “peeps” and it is currently classified as “Least Concern” (LC; IUCN 2016); that status should be revisited following molecular studies aimed at determining the number of evolutionary lineages in this widespread, but poorly understood species.

Sites and specimens: AS 5: KU 319440–48, AN 3: CAS 133542, CAS 133623.

##### *Kalophrynus sinensis* Peters, 1867

**Figure 20.**
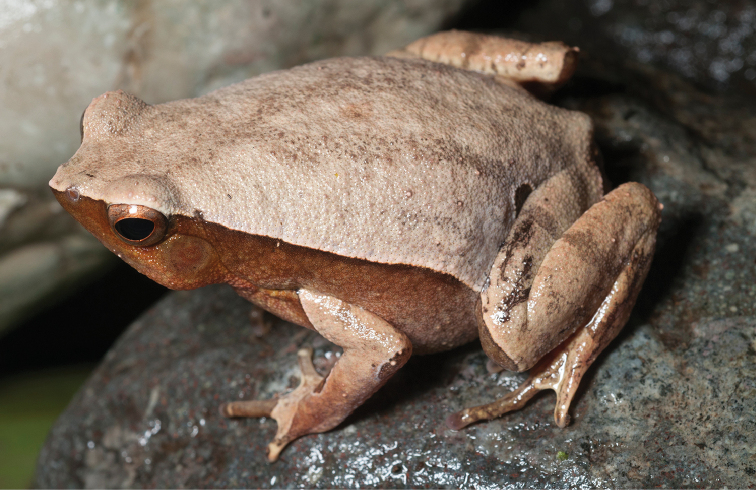
*Kalophrynus sinensis* male (KU 333148) from Mt. Hilong-hilong Municipality of Remedios T. Romualdez, Agusan del Norte Province. Photo: RMB.

Until recently, *Kalophrynus pleurostigma* Tschudi, 1838 (Fig. 20) was considered a widespread species from the Philippines, Indonesia, Singapore, Malaysia, Thailand and Myanmar (AmphibiaWeb 2013). Zug (2015) clarified the status of the Philippine population and resurrected *Kalophrynus sinensis*—the oldest available name, *Calophrynus pleurostigma* var. *Sinensis* Peters (1867)—for the Philippine lineage. In doing so, he considered all Philippine populations to be conspecific, and thus, considered Boettger’s 1897 *Calophrynus acutirostris* (type loc.: Samar Island) and Stejneger’s (1908) *Kalophrynus stellatus* (Basilan Island) to be junior synonyms of *Kalophrynus sinensis*. If future genetic studies determine that significant geographic structure exists to warrant the recognition of allopatric islands populations, additional names may need to be resurrected from the synonymy of *Kalophrynus sinensis* (Zug 2015). *Kalophrynus sinensis* frequently is encountered in the rainy season, calling while floating in temporary pools or water filled cavities in a variety of habitats of varying levels of disturbance. It is classified as “Least Concern” (LC; IUCN 2016). Our specimens were found floating in stream side pools, water-filled depressions on rocks, and even in water collected in half coconut shells in agricultural areas.

Sites and specimens: AN 10: KU 333152–53; AN 11: KU 333148, AN 12: KU 333149–51; AS 5: KU 319467–69, KU 314054–55; C 10: KU 301847–48, KU 327822–30; C 13: KU 309647–53, KU 310352; C 14: KU 309654–57; C 3: CAS-SUA 24093; C 7: CAS-SUA 22855, USNM 305724–26. D 4: KU 305872; D 5: KU 309969–73; AN 3: CAS 133567–69, CAS 133600–03, CAS 133633, CAS 133771–76, CAS 137516–18; C 1: CAS-SUA 24064–68, CAS-SUA 24094–96, CAS-SUA 24114–18; C 2: CAS-SUA 24069; C 3: CAS-SUA 24070; C 7: CAS 139035–36, CAS 139172.

##### *Kaloula conjuncta meridionalis* Inger, 1954

**Figure 21.**
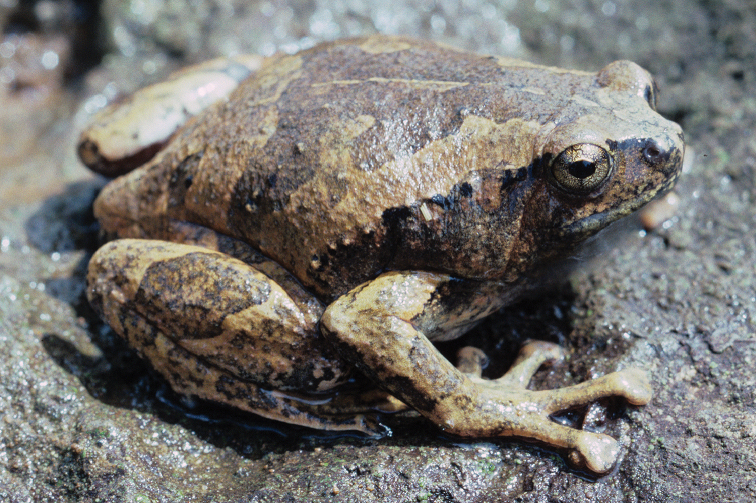
*Kaloula conjuncta meridionalis* females (KU 319452–53) were collected from Mt. Magdiwata, Barangay Bayugan II, Municipality of San Francisco, Agusan del Sur Province. Photo: RMB (TNHC 59636; from Municipality of Toril, Davao City Province).

The curious, patchy, and unpredictable distribution of the Mindanao PAIC representative of the *Kaloula conjuncta* group (Inger 1954; Alcala and Brown 1998) leads us to think that this “subspecies” (Fig. 21) has a distinctly different natural history than the other taxa in this group (e.g., *Kaloula conjuncta conjuncta* from Luzon and *Kaloula conjuncta negrosensis* from the western Visayan islands of Panay, and Negros). In a recent phylogenetic analysis, Blackburn et al. (2013) demonstrated the monophyletic and genetic distinctiveness of each of the subspecies of *Kaloula conjuncta*, ﻿suggesting that each may warrant specific status. If this action is followed by taxonomists, the conservation status of *Kaloula conjuncta meridionalis* will need to be independently assessed. *Kaloula conjuncta* currently is considered “Least Concern” (LC; IUCN 2016) and its subspecies have not been assessed individually. Frogs of the *Kaloula conjuncta* Complex emerges at the start of the rainy season, forming large breeding aggregations. At other times of the year they virtually undetectable (RMB *personal observation*), which will be a challenge for future conservation status assessments; we consider this taxon to be a distinct evolutionary lineage, worthy of species rank; as such we classify it as Data Deficient (DD; IUCN 2010).

Sites and specimens: AS 2: USNM 229325–29; AS 5: KU 319451–53; C 13: KU 319364; C 14: KU 309658; SN 1: CAS 89800, CAS-SUA 16166–69.

##### *Kaloula picta* (Duméril & Bibron, 1841)

**Figure 22.**
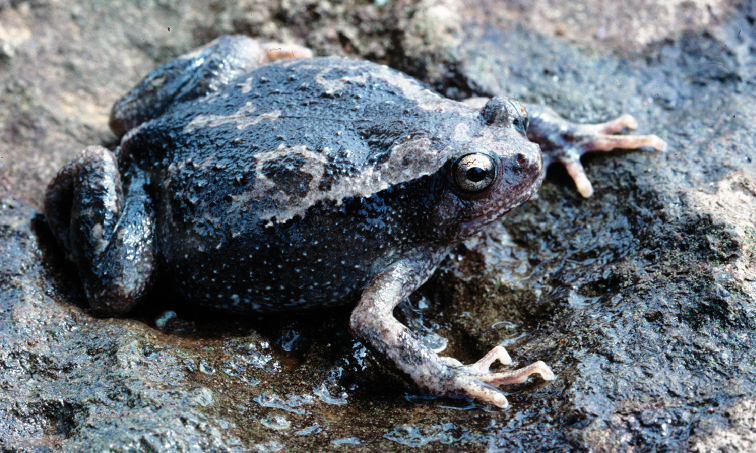
*Kaloula picta* were observed (specimens not collected) at the Municipality of Gingoog City, Misamis Oriental Province. (TNHC 56371; from Municipality of Toril, Davao City Province). Photo: RMB.

Distributed throughout the Philippines and formerly quite commonly encountered in dense aggregations in rice fields and temporary bodies of water in the rainy season, *Kaloula picta* (Fig. 22) has been considered “Least Concern” (LC; IUCN 2016). We consider *Kaloula picta* to be “Data Deficient” (DD; IUCN 2010), owing to lack of recent field observations confirming the species’ actual (versus presumed; IUCN 2016) distribution and the extent to which it may be threatened by invasive species. Unevaluated threats to this species may include competition and/or direct predation from recently introduced invasive species *Kaloula pulchra* and *Hoplobatrachus rugulosus* (Diesmos et al. 2006, 2015). Blackburn et al. (2013) demonstrated the genetic uniformity of this species throughout the Philippines, which would tend to refute Inger’s (1954) suggestion that regional morphological differentiation within *Kaloula picta* might eventually warrant taxonomic partitioning.

Sites and specimens: AS 4: KU 314056–57; C 12: KU 301865–76; C 14: KU 309659–61, KU 310356; D 1: USNM 229309–10.

##### *Kaloula* sp. (undescribed)

**Figure 23.**
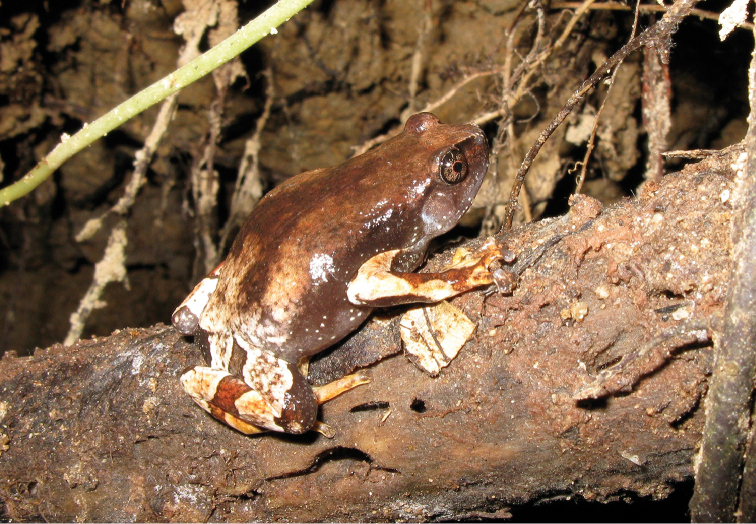
*Kaloula* sp. female (KU 319451) from 1900 masl, Mt. Balatukan, Municipality of Gingoog City, Misamis Oriental Province. Photo: ACD.

An undescribed species of forest cavity-dwelling (tree hole) *Kaloula* has been documented on Leyte, Samar islands (Blackburn et al. 2013), and northeast Mindanao (Fig. 23). Distictive “honking” vocalizations were heard during our 2012 survey of Mt. Hilong-hilong (RMB, *unpublished data*) but the source of these apparently species-specific calls was never documented because of the sheer, canyon-like terrain at Eye Falls. Nevertheless, we are certain of this new species’ identity, having traced its distinctive advertisement call to individual calling males on numerous occasions on Samar and Leyte, and so inclusion of this undescribed species in the this report is clearly advisable at this time.

Sites and specimens: none.

##### Oreophryne cf. nana Brown & Alcala, 1967

**Figure 24.**
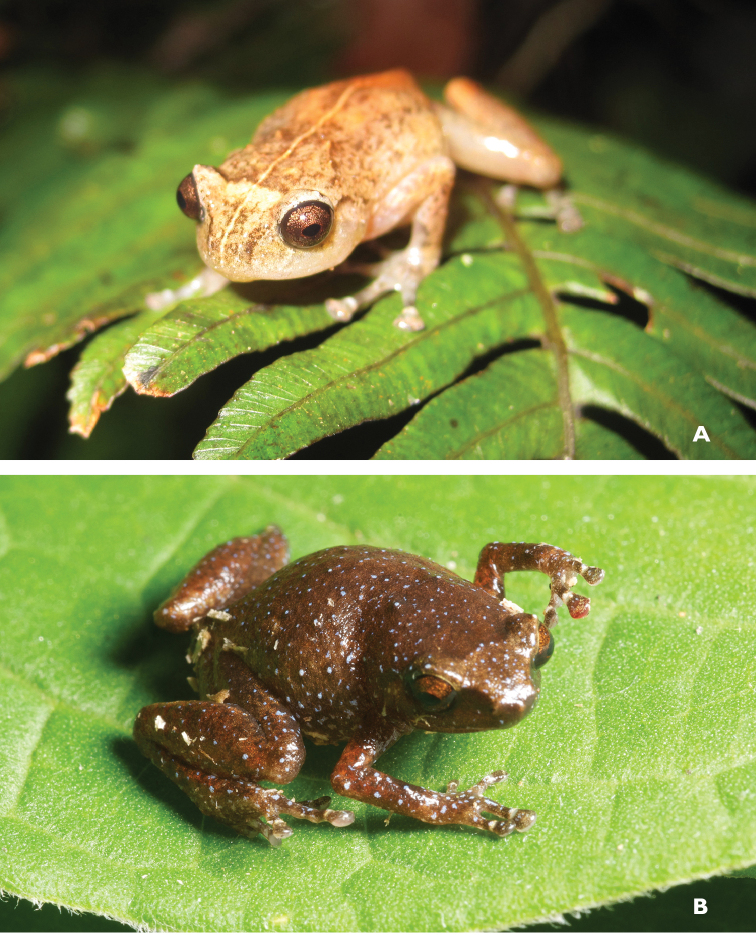
Oreophryne cf. nana male (**A** KU 334100) from 1700 masl, Mt. Lumot, Municipality of Gingoog City, Misamis Oriental Province. (Photo: KAC) and from (**B** KU 333330), male from Barangay Civoleg, Haribon Site, 1700 m, Mt. Lumot, Municipality of Gingoog City, Misamis Oriental Province.Photo: RMB

Described from Camiguin Sur Island (Brown and Alcala 1967), *Oreophryne nana* reportedly differs from *Oreophryne anulata* (from Mt. Apo) on the basis of the absence of subarticular tubercles on the hand. Now that specimens of *Oreophryne* have been collected from numerous high and low elevation sites throughout Mindanao, a comprehensive appraisal of the genetic and morphological variation in this group is overdue. Our impression is that there are several sites where the degree of distinctiveness of the subarticular tubercles varies and that this character may require additional study. For now, we refer all our Northeast Mindanao *Oreophryne* to “Oreophryne cf. nana” with the caveat that we are not at all certain of this identification. This species (Fig. 24) is listed as “Data Deficient” (DD; IUCN 2016) due to taxonomic and species distribution uncertainty and as a taxonomic reappraisal becomes available, the status for the Camiguin Sur Island and northeast Mindanao populations will need to be revisited. Specimens from our Mt. Lumot expedition were positive for chytrid fungus (Brown et al. 2012b; Diesmos et al. 2012, 2015), which leaves us with concerns for the long-term status of these populations.

Sites and specimens: AS 6: KU 314058; C 13: KU 309662–84; C 3: CAS-SUA 24089; C 7: CAS 137552–54; C 8: CAS-SUA 22055–62; MO 2: KU 333341–42, KU 334100; MO 3: KU 333178, KU 334099; MO 4: KU 333310–40.

#### Family Ranidae

##### *Pulchrana grandocula* (Taylor, 1920)

**Figure 25.**
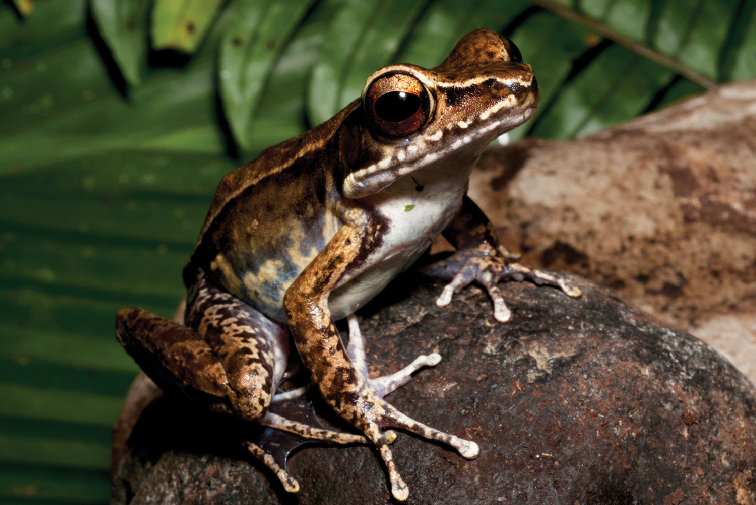
*Pulchrana grandocula* female (KU333633) from Mt. Lumot, Municipality of Gingoog City, Misamis Oriental Province. Photo: RMB.

This species (Fig. 25) is widespread, commonly encountered at high abundances, and distributed throughout the Mindanao PAIC islands (Brown and Guttman 2002; Brown and Siler 2013). Classified as “Least Concern” (LC; IUCN 2016) the species can be found in a variety of disturbed habitats and is distributed across much of the elevational relief of Mindanao. Males form choruses around pools in streams and rivers and call when water levels are relatively low; females have been observed in these same riparian habitats, but also relatively far away from water (100 m or more), sometimes perching in low branches of understory trees, and occasionally aggregating in shallow caves formed by overhanging stream banks (RMB *personal observation*). The discovery of a new, morphologically similar, and exceedingly rare stream frog species that had previously been confused with *Hylarana grandocula* (Brown and Siler 2013; Brown 2015) leads us to speculate that mountains of northeast Mindanao may also harbor undocumented populations of this second Mindanao *Hylarana* taxon. Oliver et al. (2015) recently published a phylogeny for many members of the African, Papuan, and southeast Asian members of the genus *Hylarana* and recognized “*Pulchrana*” as the available name corresponding to the *Hylarana signata* complex (*sensu* Brown and Guttman 2002; Brown and Siler 2013). Although this action is arbitrary and unnecessary and no justification for a maximally atomized classification was provided (Wiens et al. 2009; Poe 2013; Brown et al. 2015) we adopt the most recently published name.

Sites and specimens: AN 10: KU 333592–602; AN 11: KU 333551–91; AN 5: USNM 305600–01; AS 2: USNM 229359–60; AS 5: KU 319627–40; C 12: KU 302374–79; C 14: KU 309771–819, KU 310355; C 6: USNM 305733–36; C 7: USNM 305737–38; D 2: KU 306445–72; D 2: KU 306480–90; D 4: KU 306439–44, KU 306473–79; D 5: KU 310015–28, KU 310370; MO 2: KU 333612–49; MO 4: KU 333603–11; MO 5: KU 319783–89; MO 6: KU 333650–71; AN 1: CAS 133722, CAS 186127; AN 3: CAS 133382, CAS 133503–04, CAS 133553, CAS 133664–65, CAS 137535–39, CAS 139397–98, CAS 145938; C 1: CAS-SUA 24071–77, CAS-SUA 24080–86, CAS-SUA 24101–53; C 6: CAS 139173–74, CAS-SUA 23064–83; C 7: CAS 139041–43, CAS 139175; SS 2: CAS-SUA 17499.

##### *Sanguirana albotuberculata* (Inger, 1954)

**Figure 26.**
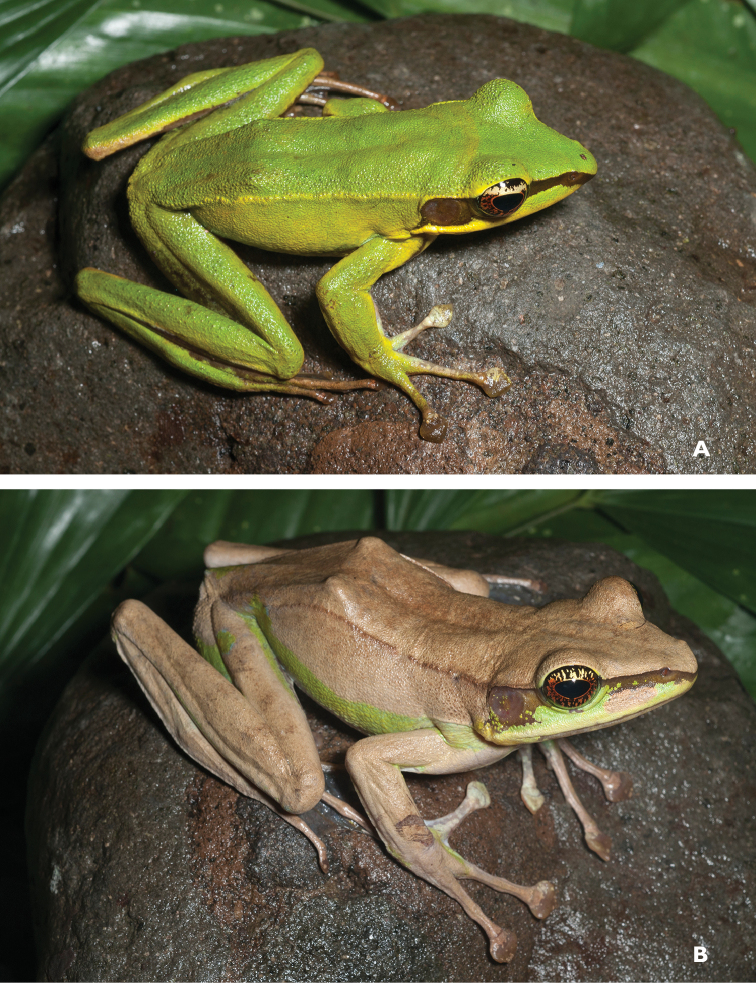
*Sanguirana albotuberculata* male (**A** KU 333000) and female (**B** KU 333001) from Mt. Hilong-hilong, Municipality of Remedios T. Romualdez, Agusan del Norte Province. Photos: RMB.

Previously considered a subspecies of “*Rana everetti*” (Inger 1954), the treatment of the Samar-Leyte-northeast Mindanao population of slender stream frog as a full species, distinct from the southwest Mindanao population (topotypic *Sanguirana everetti*) is an arrangement that was first postulated on the basis of morphometric data (Brown et al. 2000b, 2002; Fuiten et al. 2011), and has now been confirmed with genetic data (Brown et al. 2016). When reproductively active (July–August on Mt. Lumot) this species (Fig. 26) can be exceedingly common and locally abundant in riparian habitats, and usually perches in streamside vegetation, on steep banks, or large boulders, several meters from water; we have observed individuals as high as 4–5 m in riverbank trees. Lacking vocal sacs, *Sanguirana albotuberculata* males call with a slow, dull, pulsed rattle vocalization (the apparent advertisement call). Females also vocalize with a series of rapid “squeaks” and “whistles” delivered in a series of descending frequency notes (RMB *personal observation*) as has been reported two related species, *Sanguirana luzonensis* (Brown et al. 2000) and the lineage from the West Visayan islands’ (Brown et al. in review). Previously considered “Data Deficient” (DD; IUCN 2016), this species has now been recorded at a sufficient number of localities that we can evaluate it against IUCN (2010) conservation status criteria. *Sanguirana albotuberculata* does not qualify for elevated threat status; we consider it “Least Concern” (LC; Diesmos and Brown 2011; Diesmos et al. 2014).

Sites and specimens: AN 11: KU 332972–3007; MO 2: KU 333008–30, KU 333034–41, KU 333059–67; MO 4: KU 333043–58; MO 5: KU 319777–82; MO 6: KU 333031–33, KU 333042; AN 3: CAS 133422–23, CAS 133469, CAS 133501, CAS 137533–34, CAS 139394–95; AN 5: USNM 305594–97.

##### *Staurois natator* (Günther, 1858)

**Figure 27.**
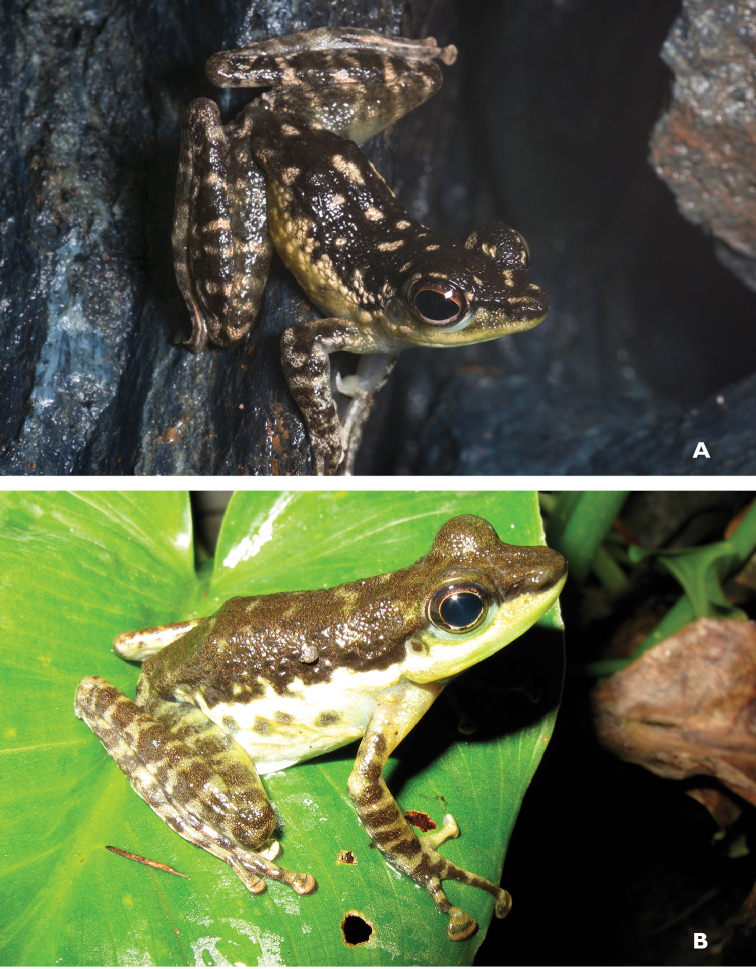
*Staurois natator* male (**A** KU 327816) from Municipality of Mambajao, Mt. Mambajao, Camiguin Sur Island (Photo: CDS) and female (**B** KU 319809) from 1900 masl, Mt. Balatukan, Municipality of Gingoog, Misamis Oriental Province. Photo: ACD.

Common throughout the Mindanao faunal region, *Staurois natator* (Fig. 27) is a frequently observed component of most amphibian communities of the southern Philippines (Alcala and Brown 1998). Arifin et al. (2011) demonstrated the distinction between Palawan faunal region populations (*Staurois nubilis*) versus those of the Mindanao PAIC (*Staurois natator*); remaining taxonomic issues include the status of Basilan populations and the unique color pattern exhibited by populations of Samar and Leyte islands (RMB *personal observation*). This species is characterized as “Least Concern” (LC; IUCN 2016).

Sites and specimens: AN 1: CAS 133683; AN 13: KU 333775–801; AN 3: CAS 133214, CAS 133264–68, CAS 133435–42, CAS 133464, CAS 133494, CAS 133555, CAS 133605–08, CAS 133658–61, CAS 133672, CAS 137525–32; AN 4: CAS 133146–47, CAS 133168–77, CAS 133230, CAS 133244–48, CAS 133282, CAS 133323, CAS 133342; AN 5: USNM 305602–11, KU 319500–07; D 2: KU 306562–91; D 5: KU 310034–66; MO 2: KU 333710–74; MO 4: KU 333686–94; MO 5: KU 319799–843; MO 6: KU 333695–709; SS 2: CAS-SUA 17497–98.

#### Family Rhacophoridae

##### Theloderma (Nyctixalus) spinosum (Taylor, 1920)

**Figure 28.**
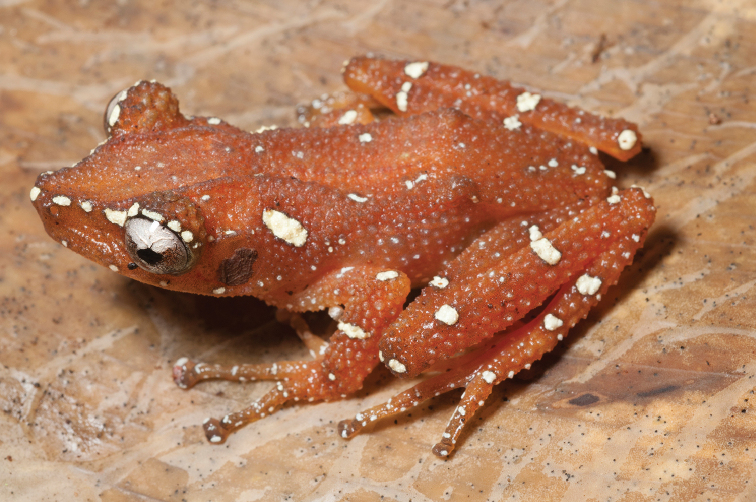
*Theloderma spinosum* female (KU 333805) from May-Impit, Mt. Hilong-hilong, Municipality of Remedios T. Romualdez, Agusan del Norte Province. Photo: RMB.

This somewhat rarely encountered species (Fig. 28) is known from Basilan, Leyte, Samar, Mindanao, and Bohol. Taylor speculated that its apparent “rarity” might be due to the species’ arboreal microhabitat preferences; he reported finding individuals underneath leaf litter inside a tree cavity (Taylor 1920a). We suspect that this species, which does not form choruses, is rare in collections because of the difficulty of localizing and tracking its soft, tonal advertisement call, and so we do not recommend elevating conservation status (*sensu* IUCN 2016) of this species on the basis of the frequency with which it has been collected historically in faunal surveys. We typically locate individuals on shrubs and saplings after triangulating (with multiple field collectors) on the source of its recognizable, but soft whistling call. *Theloderma spinosum* calls with a quiet, high frequency, pure tone chirp or a rapid series of quiet, high frequency, ascending chirps. Based on our observations this species seems to rely on primary and mature secondary forest and has been collected along elevational ranges of 300–1,100 m (Brown and Alcala 1994; Alcala and Brown 1998). On the basis of a molecular phylogenetic analysis Poyarkov et al. (2015), recommended that Nyctixalus be considered a subgenus of Nyctixalus. This species was previously classified as “Vulnerable” (VU; IUCN 2016). It has recently been reassessed (Diesmos et al. 2014) on the basis of new data and qualifies only for “Near Threatened” (NT; IUCN 2010; Diesmos et al. 2014, 2015).

Sites and specimens: AN 12: KU 333804–05; AS 5: KU 319400–02; MO 6: KU 333806; AN 3: CAS 133561, CAS 133629–32, CAS 139319.

##### *Philautus acutirostris* (Peters, 1867)

**Figure 29.**
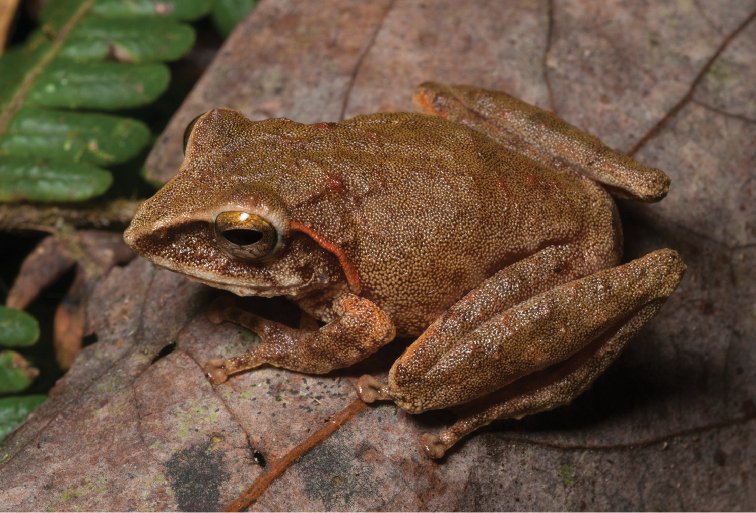
*Philautus acutirostris* female (KU 334110) from May-Impit, Mt. Hilong-hilong, Municipality of Remedios T. Romualdez, Agusan del Norte Province. Photo: RMB.

This small shrub frog (Fig. 29) is one of the most conspicuous, locally abundant, and commonly recorded montane forest frogs—provided that field workers recognize its distinctive advertisement call (a rattle, with decremental pulse rate), conduct surveys on nights following heavy rains, and search its preferred microhabitat (small leaf perches, including undersides of leaves, 0.3–1.0 m above forest floor). It is known from Jolo and Basilan islands of the Sulu Archipelago, and throughout the entirety of Mindanao Island from the extreme southwestern Zamboanga City area (RMB, ACD, CDS, and MBS *personal observations*) to the mountains of the northeast (Brown and Alcala 1994). As noted by Diesmos et al. (2014), the species IUCN conservation status (“Vulnerable:” B1ab(iii); IUCN 2016) is incorrect because no actual population research has ever confirmed the “populations trend decreasing due to continuing decline in the extent and quality of its forest habitat” justification (IUCN 2016). We found this species to be quite common, in dense aggregations, even in highly disturbed matrices of regenerating second growth and shifting agriculture on the lower slopes of Mts. Balatukan, Magdiwata, Hilong-hilong, and Lumot. Characterizing this species’ range as “severely fragmented” also appears to be erroneous because it is generally a mid- high-montage species (and, thus, has a naturally discontinuous distribution on isolated mountains). *Philautus acutirostris* was screened extensively for fungal; pathogens in 2010–2012 and we detected no signs of infection by chytrid fungus.

Sites and specimens: AN 3: CAS 133262, CAS 133394, CAS 133576, CAS 133649, CAS 133650; AN 4: CAS 133140, CAS 133164–67, CAS 133207, CAS 133211–12, CAS 133259, CAS 133289–90, CAS 133298–300, CAS 133308–09, CAS 133311–12, CAS 133334–36; AN 5: NMNH 497019–21; AN 12: KU 334108–55, KU 334157; AN 13: KU 334156.

##### *Philautus poecilius* Brown & Alcala, 1994

**Figure 30.**
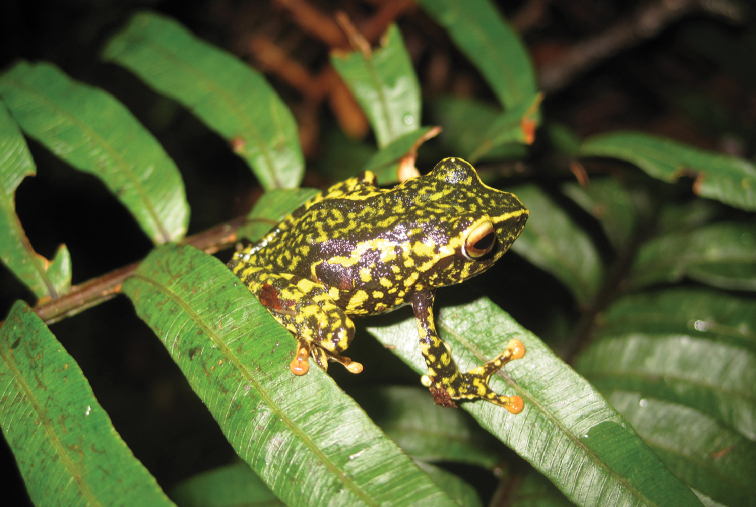
*Philautus poecilus* male from Mt. Lumot, Municipality of Gingoog, Misamis Oriental Province. Photo: KAC.

This high elevation Mindanao endemic shrub frog (Fig. 30) calls from a variety of perches in primary forests above 700 or 800 meters. It has been recorded from forests of eastern Mindanao (Brown and Alcala 1994; Plaza and Sanguila 2015) to the more central high elevation forests of Mt. Lumot to Mt. Malingdang of western Mindanao (Nuñeza et al. 2010). This is a species (in contrast to *Philautus acutirostris* and *Philautus worcesteri*), which actually does appear to be limited to relatively smaller areas of occurrence towards the higher elevation reaches of montane habitats. Although we emphasize that a naturally discontinuous geographical distribution does not constitute a “severely fragmented” range (IUCN 2016), that this species occurs in multiple protected areas, and that additional montane localities are certain to be added to the species geographical range once the mountains of Mindanao are properly surveyed, we follow Diesmos et al. (2014) and hold downgrading of *Philautus poecilius*’s conservation status in abeyance because available data do suggest that this species is a high elevation specialist, with a distribution limited to original forest. At one high elevation site (Mt. Lumot) where we found this species, we detected high prevalence and levels of chytrid infection in other species of treefrogs; *Philautus poecilius*, however, was negative for chytrid.

Sites and specimens: AN 6: CAS 133524–26, CAS 133530, CAS 133532, CAS 133543–44; MO 3: KU 334208–23.

##### *Philautus surrufus* Brown & Alcala, 1994

**Figure 31.**
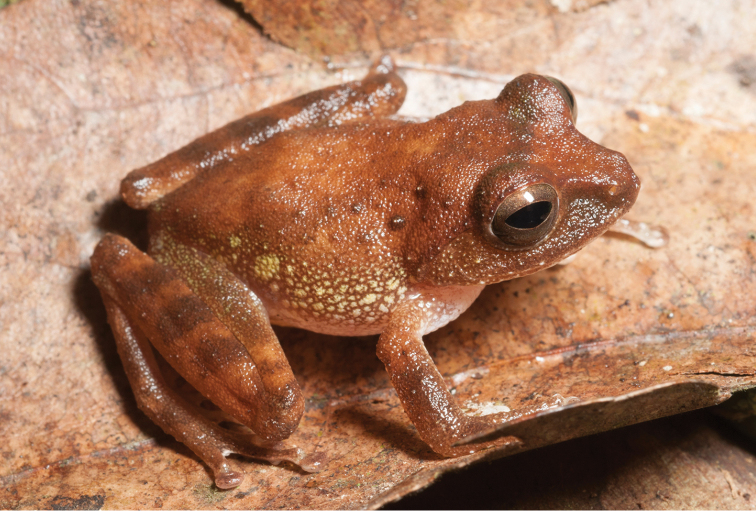
*Philautus surrufus* female from May-Impit, Mt. Hilong-hilong, Municipality of Remedios T. Romualdez, Agusan del Norte Province. Photo: RMB.

Described originally from Dapitan Peak (10 km from Masawan, Misamis Occidental Province), this species (Fig. 31) is also known from another site in the same province (Mt. Malingdang; Nuñeza et al. 2010), from Mt. Kitanglad (IUCN, 2016) and now from our work on Mt. Lumot (Misamis Oriental Province), constituting a substantial extension of its range, to the east. When Diesmos et al. (2014) downgraded from “Endangered” (EN; IUCN 2016) to “Vulnerable” (VU; IUCN 2016), they found that the species no longer qualified for the higher threat category principally on the basis of its much wider area of occurrence, and also the fact that it appears to tolerate at least some levels of disturbance to its preferred upper montane forest habitat.

Sites and specimens: MO 2: KU 334159–65; MO 3: 334158; MO 5: KU 321835–42.

##### *Philautus surdus* (Peters, 1863)

**Figure 32.**
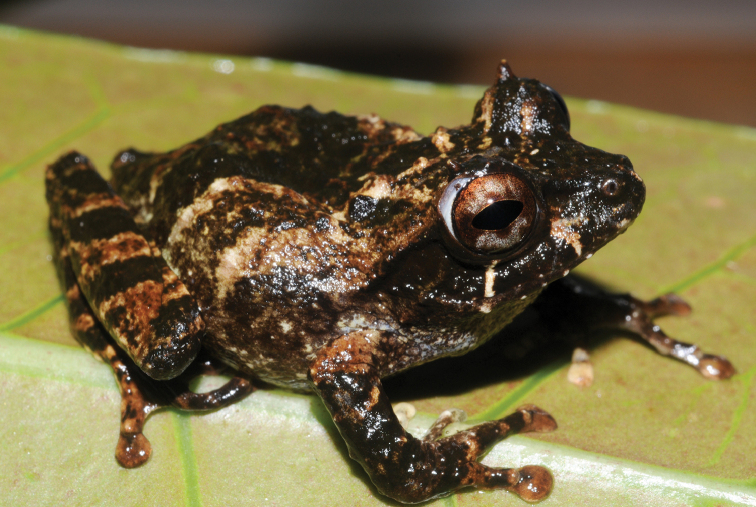
*Philautus surdus* male from May-Impit, Mt. Hilong-hilong, Municipality of Remedios T. Romualdez, Agusan del Norte Province. Photo: RMB.

The presence of *Philautus surdus* (Fig. 32) on Mindanao is attributed exclusively to specimens from several sites in Agusan Del Norte province. Elsewhere in the Philippines, this species has been recorded from Bohol, Polillo, Catanduanes, and throughout Luzon (Brown and Alcala 1994). Considered “Least Concern” (LC; IUCN 2016) because of its wide distribution across multiple islands and including many protected areas. However, we emphasize that Philippine *Philautus* are notoriously difficult to identify (because of their similar size, variable appearance, and because most species exhibit color pattern polymorphism and have similar “crunch” mating calls), and we would not be surprised if future molecular data revealed the presence of multiple species masquerading within this oddly widely distributed species.

Sites and specimens: AN 3: CAS 133263, CAS 133646–47, CAS 133698–99, CAS 133809–10, CAS 133791, CAS 133793; AN 4: CAS 133163, CAS 133199–01, CAS 133343, CAS 133565, CAS 182565, CAS 182568, CAS 183202–04; AN 5: NMNH 305622–25; AN 12: KU 333815–20, KU 333822–23.

##### *Philautus worcesteri* (Stejneger, 1905)

**Figure 33.**
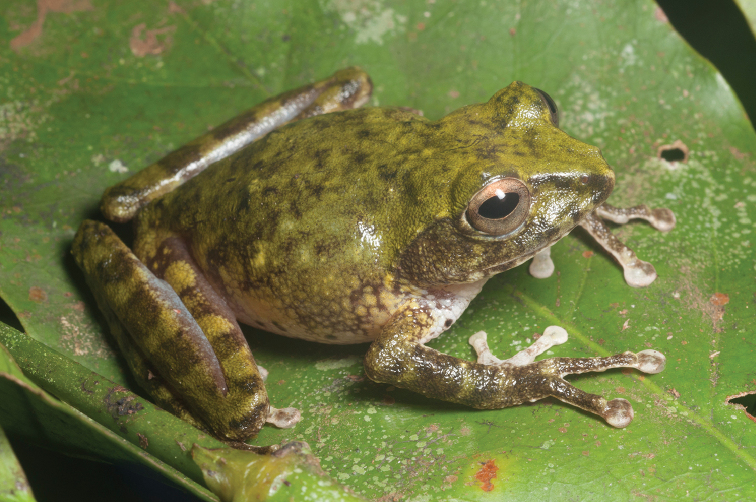
*Philautus worcesteri* male (KU 334207) from 1200 masl, Shrine Site, Municipality of Gingoog, Mt. Lumot, Misamis Oriental Province. Photo: RMB.

Brown et al. (1998) resurrected *Philautus worcesteri* from the synonomy of *Platymantis guentheri* (where it was placed by Inger [1954]). Once recognized as the distinctive large-bodied *Philautus* of Mindanao, it was recorded at numerous montane sites throughout the island (Brown and Alcala 1994) and since has been documented at a variety of sites in the island’s northeast and southwest. This species (Fig. 33) no longer qualifies for a listing of “Vulnerable” (VU; IUCN 2016) because (1) it is no known from a much greater area of occurrence than when it was originally assessed (IUCN 2004), (2) it is known from many lower elevation areas (originally considered restricted to 800–2,000 m), and (3) because it has recently been recorded at several highly disturbed agro-forest, lower-elevation sites (Zamboanga City area, lower slopes of Mts. Lumot and Hilong-hilong). We therefore classify it as “Near Threatened” (NT; IUCN, 2010) and we urge field workers to focus on its distinctive advertisement call (a loud, “crunch,” sounding to the human ear like “Yaak!,” repeated two or three times) when conducting future surveys. We suspect that this species has, in some past studies, not been detected because it perches higher and calls less frequently under drier atmospheric conditions. We screened this species for chytrid fungus at multiple sites between 2010 and 2012 and no infection was detected.

Sites and specimens: AN 4: CAS 133237, CAS 133252, CAS 133305-06, CAS 183415; AN 5: 497022; AN 6: CAS 1333521–23, CAS 133533–34; AN 12: KU 334175; D 5: KU 310010–310014; MO 2: KU 334182–334207, 334224; MO 3: KU 334158; KU 334177–334181; MO 5: KU 321845, KU 321847.

##### *Polypedates leucomystax* (Gravenhorst, 1829)

**Figure 34.**
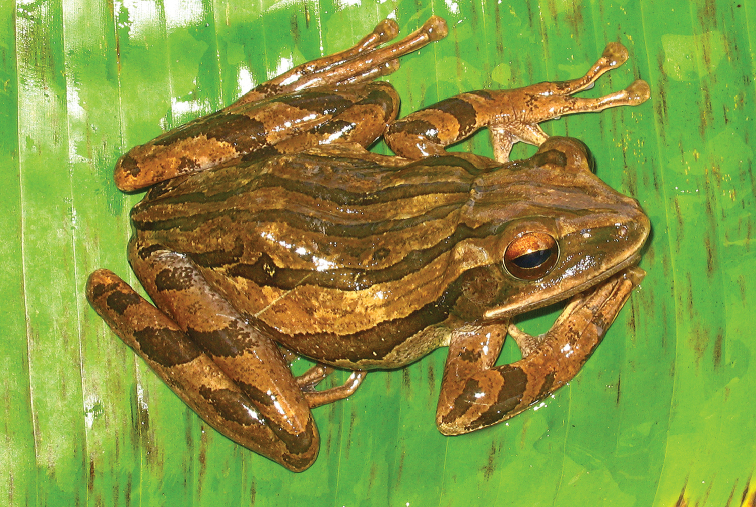
The frog *Polypedates leucomystax* is represented in the Philippines by at least two highly divergent mitochondrial gene lineages (Brown et al. 2010); one, shown here, is widely distributed from northern Luzon to northern Mindanao. Photo: K. Hesed (from Isabela Province, Luzon; KU 307625).

Common and distributed throughout the archipelago, *Polypedates leucomystax* (Fig. 34) is a species that persists well in disturbed habitats, is ubiquitous in agricultural areas, and is considered “Least Concern” (LC; IUCN 2010, 2016). Philippine populations are composed of two divergent and unrelated mitochondrial gene lineages (Brown et al. 2010b), one of which is limited to the Mindanao faunal region, Borneo, and the Malay Peninsula. The taxonomic status of these two lineages remains unresolved (Brown et al. 2010b).

Sites and specimens: AN 1: CAS 133712–25; AN 10: KU 333068–72; AN 3: CAS 133562, CAS 133626–28, CAS 133643, CAS 133667–71, CAS 137520–24; AS 2: USNM 229361–67; AS 4: KU 314079–86; AS 6: KU 314078; C 1: CAS-SUA 24055, CAS-SUA 24111–13; C 12: KU 302450; C 14: KU 309820–21; C 6: CAS-SUA 23060–63; C 7: CAS-SUA 23045; C 8: CAS-SUA 23046–59; D 1: USNM 229314–18; MO 5: KU 319888–91; MO 6: KU 333073–96.

##### *Kurixalus appendiculatus* (Günther, 1858)

**Figure 35.**
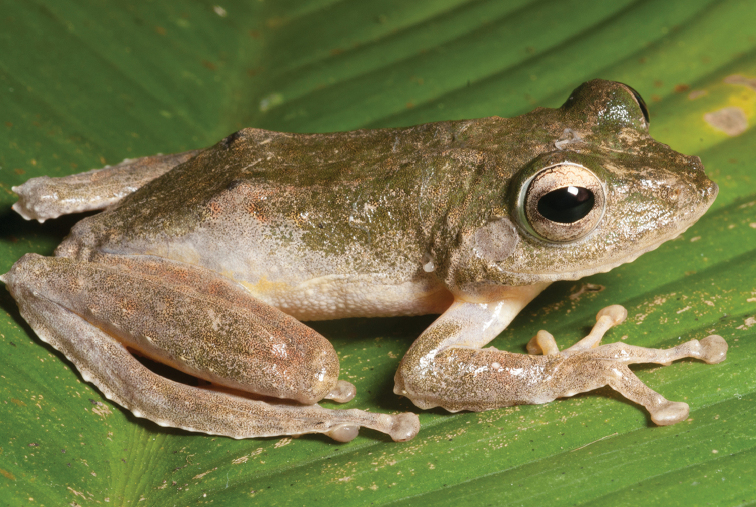
*Kurixalus appendiculatus* has been recorded in Agusan del Sur, Camiguin, and Misamis Oriental provinces (Fig. 1). Photo: RMB (from the Municipality of Balangiga, Samar; specimen deposited at KU).

Previously classified as a species of *Rhacophorus*, *Kurixalus appendiculatus* (Fig. 35) is distributed across much of the archipelago’s eastern island arc (Mindanao, Dinagat, Samar, Leyte, and northern Luzon), but is conspicuously absent on the intervening Bicol Peninsula (Brown and Alcala 1994). In a recent phylogeographic analysis of samples from throughout this range, Gonzales et al. (2014) suggested a novel island colonization scenario and provided some evidence to suggest that *Kurixalus appendiculatus* in the Philippines may be composed of several independent evolutionary lineage, which eventually may be recognized as separate species. Based on this information and the unresolved taxonomic status of the three major Philippine lineages, Diesmos et al. (2014) recommend classification of Philippine populations of *Kurixalus appendiculatus* as “Data Deficient” (DD; IUCN 2016). The resolution of the status of these populations is an urgent conservation priority for the immediate future. This is a swamp, ephemeral pool, and stagnant water specialist species, which most likely lays its eggs in mud; the tadpoles are unknown.

Sites and specimens: AS 5: KU 319454–66; AS 6: KU 314087; C 13: KU 309835–59; MO 6: KU 333802.

##### *Rhacophorus bimaculatus* (Peters, 1867)

**Figure 36.**
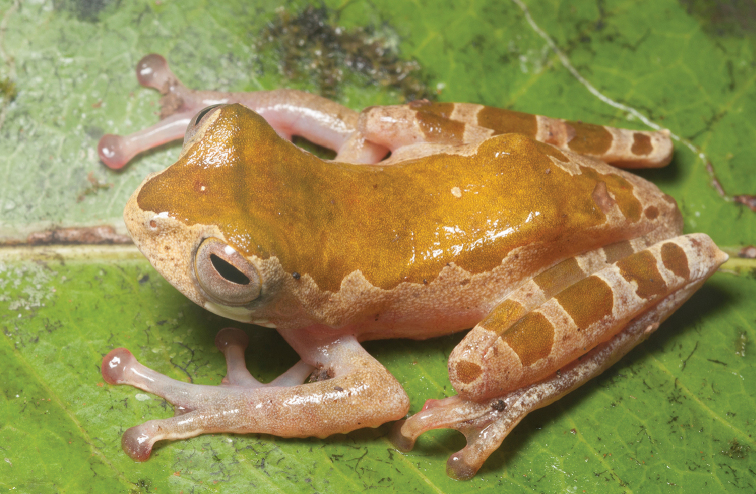
*Rhacophorus bimaculatus* male (KU 333195) from Mt. Lumot, Municipality of Gingoog City, Misamis Oriental Province. Photo: RMB.

This common tree frog (Fig. 36) inhabits overhanging understory vegetation surrounding rapidly cascading streams in lower- to mid-montane forests. Its distinctive advertisement call is a single brief, high frequency, shrill chirp—and can be heard over the sound of waterfalls (its favored microhabitat). Previously considered uncommon, this species is now appreciated for its very specific microhabitat preference, wherein it can be predictably encountered by experienced field workers. Originally classified in 2004 as “Vulnerable” (VU; IUCN 2016), this species now qualifies only for “Near Threatened” (NT; IUCN 2010; Diesmos et al. 2014) as a result of the numerous new localities at which it has been recorded (Gonzales et al. 2014), and the predictability with which it can be found, now that its habitat is known and can be purposefully surveyed (Diesmos et al. 2014, 2015).

Sites and specimens: AN 11: KU 333154–67; AN 12: KU 333171; AN 13: KU 333168–70; AS 5: KU 319574–91; D 5: KU 310032–33; MO 2: KU 333180–249; MO 3: KU 333172–77, KU 333179; MO 5: KU 319846–87; MO 6: KU 333250–58; AN 3: CAS 133395, CAS 133427, CAS 133558–60, CAS 133621, CAS 133655–6, CAS 133666, CAS 133787–88, CAS 139399, CAS 180678–79; AN 4: CAS 133178–80, CAS 133251, CAS 133295–97; AN 5: CAS 182564.

##### *Rhacophorus pardalis* (Günther, 1858)

**Figure 37.**
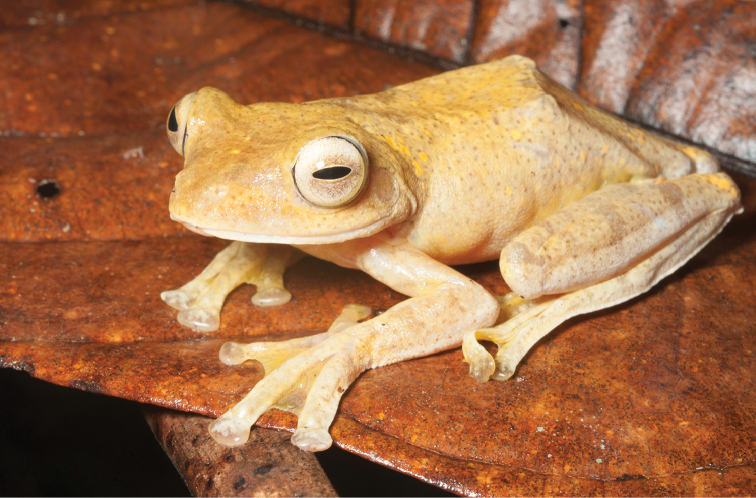
*Rhacophorus pardalis* has been recorded from Agusan del Sur Province and Camiguin Sur Island. Photo: RMB (from the Municipality of Burauen, Leyte Island; specimen deposited at KU).

This swamp- and ephemeral pond-breeding species (Fig. 37), as presently conceived, is distributed throughout the archipelago (Brown and Alcala 1994; Alcala and Brown 1998; Gaulke 2011). Outside of the country, populations referred to the same species have been reported from Peninsular Thailand, Borneo, and Sumatra (Manthey and Grossman 1997). Patchily distributed, this species is most often encountered perched in vegetation above stagnant pools such as feral pig wallows, and nearly any water-filled in original or even highly disturbed forest (RMB *personal observation*). Because of its wide distribution, this species is considered “Least Concern” (LC; IUCN 2016).

Sites and specimens: AS 5: KU 319647–50; C 1: CAS-SUA 24097–100; C 13: KU 309824; C 14: KU 309822–23, KU 309825–34.

#### Family Ichthyophiidae

##### *Ichthyophis mindanaoensis* (Taylor, 1960)

**Figure 38.**
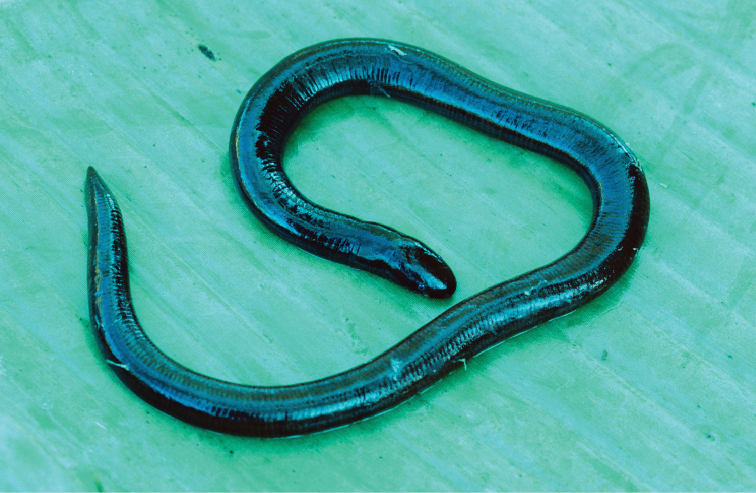
*Ichthyophis mindanaoensis* has been collected in Mt. Magdiwata, Municipality of San Francisco, Agusan del Sur Province. Photo: RMB (from Davao City Province, specimen deposited in Cincinnati Museum of Natural History).

A single specimen putatively identified as *Ichthyophis mindanaoensis* (Fig. 38) has been collected at Barangay Bayugan II, Municipality of San Francisco, Mt. Magdiwata. Collected in dry soil beneath a log several hundred meters from a small stream, this specimen appears to be a juvenile male. The taxonomy and extent of occurrence of *Ichthyophis mindanaoensis* is somewhat uncertain. Other specimens have been reported from Bukidnon (central Mindanao), Mt. Malindang (western Mindanao; most likely misidentified exemplars of *Ichthyophis glandulosus*, a close relative known from Basilan Island and the southern Zamboanga Peninsula), Davao City Province (eastern Mindanao) and South Cotabato Province (southern Mindanao; Taylor 1920a, 1960, 1965; Inger 1954, Diesmos et al. 2011, 2014, 2015). A much-needed effort to reexamine and reconsider the range of variation exhibited in traditional morphologically diagnostic characters (Taylor 1960, 1965) would now be possible, given the accumulation of specimens from throughout the island. Given the current uncertainty, however, concerning its distribution taxonomic status, this species is considered by the IUCN to be “Data Deficient” (DD; IUCN 2016).

Sites and specimens: AS 5: KU 319433.

### Reptilia: Lizards

#### Family Agamidae

##### *Bronchocela* sp.

**Figure 39.**
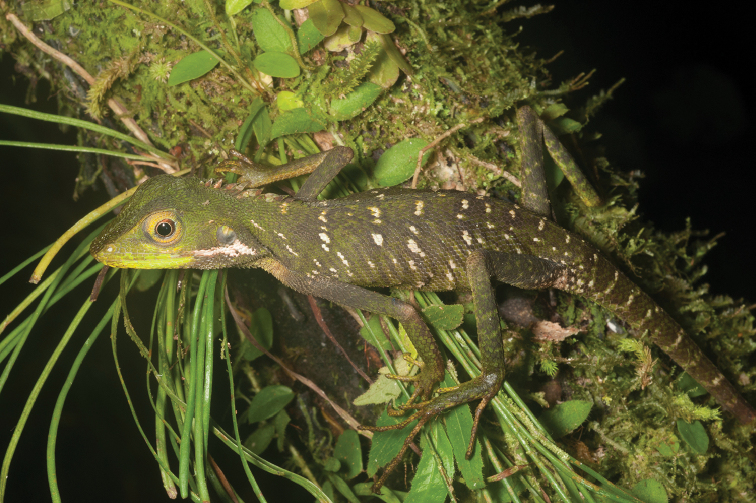
*Bronchocela* sp. male (KU 334441) from Eye Falls, Mt. Hilong-hilong, Municipality of Remedios T. Romualdez, Agusan del Norte Province. Photo: RMB.

This species (Fig. 39) was found asleep at night in shrubs and saplings 2–4 meters above the ground in secondary forest and along forest edges in agricultural areas. Individuals were especially common at site MO 6 at the edge of primary forest abutting a river. This species has a widespread distribution that includes Northeast Mindanao, Camiguin Sur, Dinagat and Siargao islands. At present, with the taxonomy of this group confused and unassessed using molecular data (Hallermann 2005), the conservation status of “*Bronchocela cristatella*” remains unassessed (IUCN 2016). If this taxon is as widespread as currently conceived (Hallermann 2005), its extremely broad distribution throughout the Philippines and surrounding Sundaic landmasses, plus its constant presence in a wide variety of forested and unforested habitats, would qualify it for “Least Concern” (LC) using IUCN conservation status assessment criteria (IUCN 2010). We recommend treating this species as “Data Deficient” (DD; IUCN 2016) until a formal taxonomic review using both morphological and genetic data, can be performed. We suspect Philippine populations are not conspecific with the lineage at the type locality (Java Island, Indonesia); although Taylor named *Bronchocela marmorata* from northern Luzon, that name most likely would not apply to the distinctive population on the Mindanao PAIC (see also: Grismer et al. 2015, 2016).

Sites and specimens: AN 12: KU 334001; AN 5: USNM 497026; C 13: KU 309860–62; D 5: KU 310374; MO 2: KU 334002; MO 6: 334003–09; AN 1: CAS 133734; AN 3: CAS 133565; C 1: CAS-SUR 28348; C 2: CAS-SUR 28335; C 6: CAS-SUR 26132, CAS-SUR 26138.

##### *Draco bimaculatus* (Günther, 1864)

**Figure 40.**
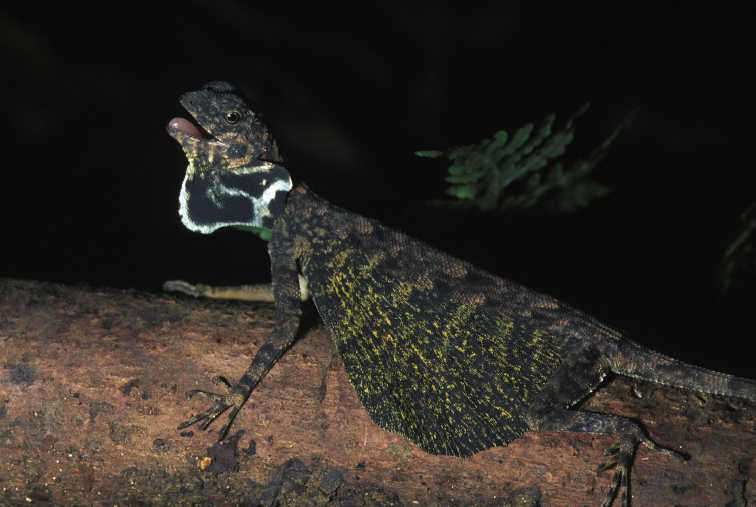
*Draco bimaculatus* is widely distributed throughout low elevation and coastal areas of northeast Mindanao. Photo: RMB (female from the Municipality of Bilar, Bohol Province, Bohol Island (specimen deposited in PNM).

This species occurs throughout the Mindanao and Sulu faunal regions (McGuire and Alcala 2000). We encountered *Draco bimaculatus* (Fig. 40) in both primary and secondary forests as well as at the edges of coconut plantations immediately adjacent to forest; our observations are consistent with those of McGuire and Alcala (2000). The maximum elevational extent of this species appears to extend up to 990 m in elevation on Mt. Hilong-hilong. We observed individuals of this species low on trunks along the edges of primary forest, during the day; however one individual was found asleep on a branch at night. This species is classified by the IUCN as “Least Concern” (LC; IUCN 2016).

Sites and specimens: AN 10: KU 333951, KU 333980; AN 12: KU 333978–79; AS 2: USNM 229368–70; AS 5: KU 319645–46; AS 6: KU 314088–89; D 2: KU 305587; D 4: KU 310085–89; D 5: KU 310069–84; MO 6: KU 333952–77; AN 1: CAS 133685–6; AN 3: CAS 154695.

##### *Draco cyanopterus* Peters, 1867

This species was quite common in coconut plantations and, like *Draco bimaculatus*, was found far from forest in a few instances. This species is classified by the IUCN as “Least Concern” (LC; IUCN 2016) and its population size may be increasing due to the expansion of Mindanao’s ubiquitous coastal coconut plantations.

Sites and specimens: AN 15: KU 333989–97; AS 5: KU 319642–4; C 11: KU 309942–43; C 13: KU 309939–41; C 15: KU 309937–38, KU 309944–45; D 2: KU 305589–93; D 5: KU 310180; MO 5: KU 319949; MO 6: KU 333981–88; C 1: CAS-SUR 28200, CAS-SUR 28339; C 2: CAS-SUR 28349.

##### *Draco mindanensis* Stejneger, 1908

**Figure 41.**
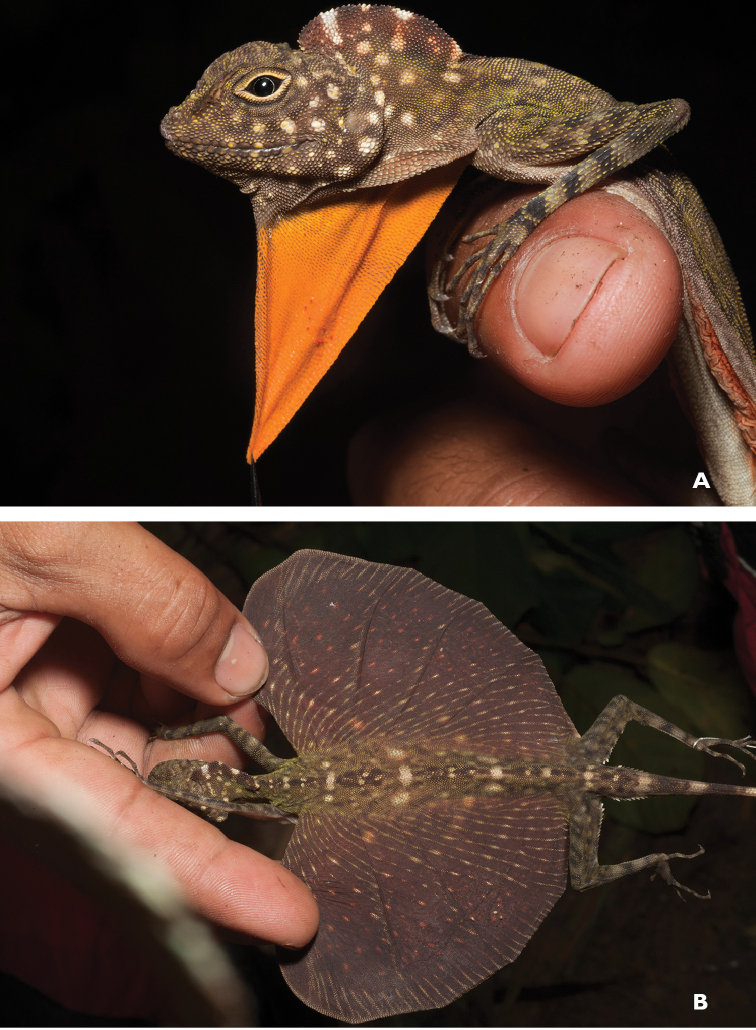
*Draco mindanensis*, a seldom-recorded forest species from Agusan del Norte and Agusan del Sur Provinces. **A** Close-up of head, male specimen **B** Male patagial coloration. Photos: RMB (from the Municipality of Balangiga, Samar Island; specimen deposited at KU).

This species (Fig. 41) is rarely encountered in the wild and the extent of its occurrence is poorly known; it has been documented from Mindanao, Leyte, Samar, Dinagat and Siargao isands (Taylor 1922a; Ross and Lazell 1990; Smith 1993a; McGuire and Alcala 2000; Realubit et al. 2015). Specimens have only been observed in primary and mature secondary growth forest habitats, active during the day on trunks, usually quite high above the ground (5–10 m above the ground; RMB *personal observation*). A true forest obligate, this phylogenetically distinct (McGuire and Kiew 2001) species qualifies for formal recognition as a “Vulnerable” (VU; IUCN 2010, 2016) conservation status taxon. It is noteworthy that biologists have always considered this species to be rare (for review, see McGuire and Alcala 2000) and almost a century ago, following several years of near-continuous fieldwork in heavily forested central Mindanao, Taylor (1922a) had only collected a few specimens.

Sites and specimens: AS 5: KU 319641; AN 1: CAS 133684; AN 3: CAS 133566.

##### *Draco ornatus* (Gray, 1845)

**Figure 42.**
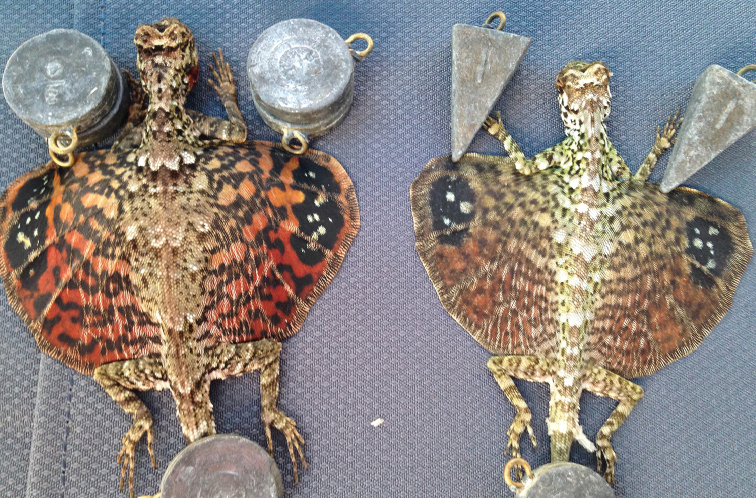
*Draco ornatus* has been recorded only from Dinagat Island and Agusan del Norte Province. Photos: RMB (**A** dorsal; **B** ventral, from the Municipality of Burauen, Leyte Island; specimens deposited at KU).

Known from Mindanao, Dinagat, Samar, Leyte and Bohol islands (McGuire and Alcala 2000), this species (Fig. 42) has been characterized as a primary and secondary forest inhabitant, that can also occasionally be observed in coconut palm plantations immediately adjacent to forest (McGuire and Alcala 2000). Like all *Draco*, it feeds exclusively on ants and termites. It is categorized as “Least Concern” (LC; IUCN 2016) for conservation planning purposes.

Sites and specimens: D 5: KU 310067–68; AN 4: CAS 133151–52, CAS 133254.

##### Gonocephalus cf. interruptus

**Figure 43.**
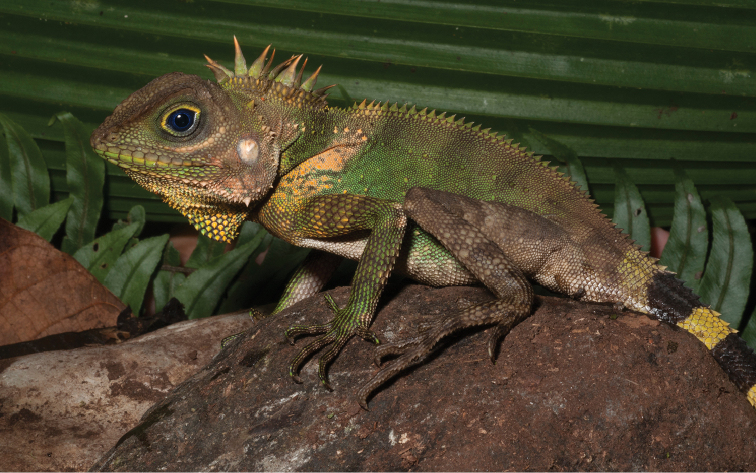
Gonocephalus cf. interruptus male (KU 334325) from Mt. Lumot, Municipality of Gingoog, Misamis Oriental Province. Photo: RMB.

*Gonocephalus interruptus* is the only species of *Gonocephalus* in the Philippines with a type specimen bearing specific locality data indicating that it was collected on Mindanao Island (although where on Mindanao is unclear). Precise type locality data are unavailable for *Gonocephalus semperi* and *Gonocephalus sophiae*, species originally reported only to have been originally collected from “The Philippines.” Although recent taxonomists and biogeographers have referred to specimens from Mindoro, Mindanao, and Bohol to *Gonocephalus semperi* and others from Luzon, Mindanao and the western Visayas (Negros, Panay, Masbate) to *Gonocephalus sophiae*, both species’ original descriptions were not accompanied by specific locality data. Thus, detailed comparisons of the name-bearing type specimens to fresh material from known localities will be necessary to definitively determine the proper application of these names to Philippine populations. Based on the crest morphology of our specimens and comparison to original illustrations (Boulenger 1885; Taylor 1922a, 1923) and their known provenance (Mindanao), we treat this taxon as most likely representative of *Gonocephalus interruptus* (Fig. 43). We concede that additional phylogeny-based taxonomic review will be required to confirm this designation and properly assign this name to the phenotypically most similar population, following examination of the name-bearing type (Boulenger 1885). We encountered this species at night, sleeping on the trunks of small trees and saplings in both moderately disturbed mature primary and secondary forest. *Gonocephalus interruptus* is treated by the IUCN as a “Data Deficient” (DD; IUCN 2016) taxon.

Sites and specimens: AN 11: KU 334319–20; AN 12: KU 334321–23; AS 5: KU 319903–19. C 7: CAS 139034, CAS 185492, CAS-SUR 26137. C 9: KU 309869, KU 309873; C 13: KU 309863; C 14: KU 309864–72. MO 2: KU 334324; MO 6: KU 334325–28.

##### *Hydrosaurus pustulatus* Eschsholtz, 1829

**Figure 44.**
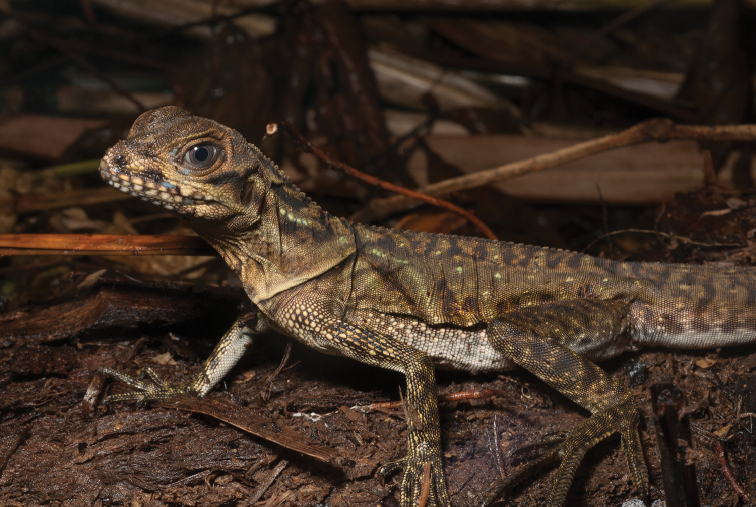
*Hydrosaurus pustulatus*, the widespread Sailfin Lizard has been recorded at numerous sites in Agusan del Norte and Misamis Oriental Provinces; the species is also known from Caminguin Sur Island. Photo: RMB (from the Municipality of Burauen, Leyte Island; specimen deposited at KU).

An inhabitant of lowland riparian corridors, coastal forests, and mangroves (Smith 1993a, Siler et al. 2014a), we encountered *Hydrosaurus pustulatus* (Fig. 44) along rivers at lower elevation sites ranging from 170–420 m above sea level. Specimens were collected at night while sleeping in rock crevices as well as during the day when this species is active. Due to the rapid and continuing decline and fragmentation of the habitat upon which it depends, this species has been classified as having a “Vulnerable” conservation status (VU; IUCN 2016). A recent phylogeographic study supports the monophyly of Philippine *Hydrosaurus pustulatus*, demonstrates that *Hydrosaurus amboinensis* does not occur in the Philippines, and indicates that there are six well structured haplotype groups across the archipelago, although given the data used in the study, not all haplotype groups corresponded with unique geographic ranges (Siler et al. 2014a). Specimens from Camiguin Sur and Dinagat island populations were recovered in several of these clades. Genetic samples from Northeast Mindanao were not available at the time of this study, and so additional research will be necessary to determine the relationship of the Northeast Mindanao populations to other populations of *Hydrosaurus pustulatus* in the Philippines, and whether or not they constitute their own Evolutionarily Significant Unit (ESU) for conservation.

Sites and specimens: AN 10: KU 334768–69; AN 12: KU 334767; C 14: KU 309874–76; C 9: KU 309877–79; D 1: USNM 229319; D 2: KU 305154, KU 305170, KU 305860–69; D 5: KU 310090–92; MO 6: KU 334770–72, KU 334803; C 6: CAS-SUR 26178; SS 3: CAS 15561.

#### Family Dibamidae

##### Dibamus cf. leucurus Taylor, 1915

**Figure 45.**
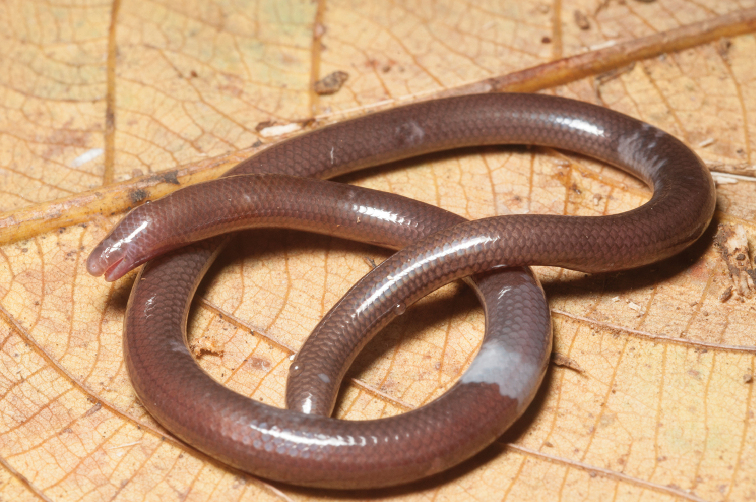
Dibamus cf. leucurus male (KU 334071) from Mt. Lumot, Municipality of Gingoog, Misamis Oriental Province. Photo: RMB.

We collected one specimen (Fig. 45) at site MO 6 on Mount Lumot; this individual matches Taylor’s description of the holotype of *Dibamus argenteus*, which he collected from Butuan, Agusan Del Norte, Mindanao Island. We suspect that the southern Philippine population currently referred to *Dibamus leucurus* is diagnosable as a distinct species, but this will need to be verified with a comparison to specimens from Sumatra, Indonesia (the type locality for *Dibamus leucurus*). To our knowledge our new specimen is now the only representative of the genus from Northeast Mindanao, as Taylor’s type specimen was destroyed during WWII (Brown and Alcala 1980). Other documented localities include Basilan Island and Camiguin Sur (CAS specimens). Our specimen was found beneath a pile of discarded coconut husks within a coconut plantation, mixed with secondary growth forest. So little is known about these secretive lizards that we are unable to comment on their conservation status and regard them as “Data Deficient” (DD; IUCN 2016).

Sites and specimens: MO 6: KU 334071; C 1: CAS-SUR 28205, CAS-SUR 28334; C 6: CAS 138253–54, CAS-SUR 26140, CAS-SUR 26293, CAS-SUR 26298; C 7: CAS 137551, CAS-SUR 26147, CAS-SUR 26199.

#### Family Gekkonidae

##### *Cyrtodactylus agusanensis* (Taylor, 1915)

**Figure 46.**
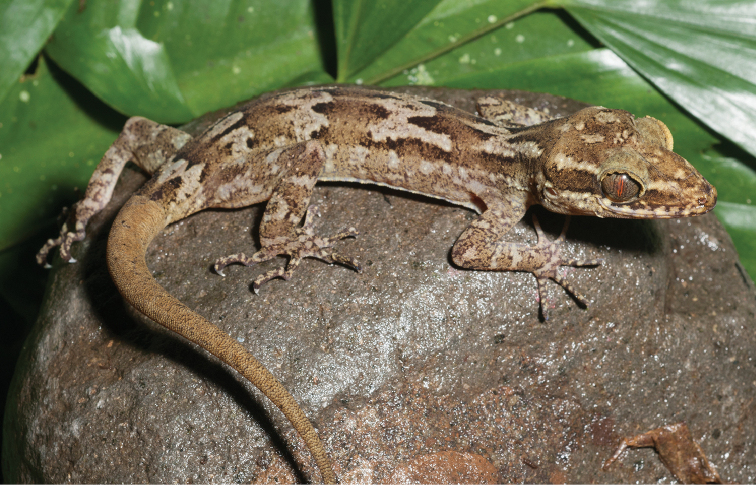
*Cyrtodactylus agusanensis* male (KU 334046) from Eye Falls, Mt. Hilong-hilong, Municipality of Remedios T. Romualdez, Agusan del Norte Province. Photo: RMB.

Formerly part of a species complex known from Mindanao, Leyte, Dinagat and Siargao islands (Taylor 1922a, Brown and Alcala 1978, Ross and Lazell 1990), the distribution of this species (Fig. 46) has since been restricted to Eastern Mindanao following taxonomic revision (Welton et al. 2010). Consistent with observations reported by Brown and Alcala (1978), Smith (1993a), and Welton et al. (2010), we collected this species among boulders on the banks of streams. We also found them to be common on vegetation adjacent to streams as well as albeit less common on vegetation well away from water. One individual was collected from inside a cave at site 12. This species is currently listed as “Least Concern” (LC; IUCN 2016).

Sites and specimens: AN 11: KU 334034, KU 334036, KU 334061–2; AN 12: KU 334044–52, KU 334054; AN 13: KU 334038–40; AN 5: USNM 496787; AS 5: KU 320014–21; MO 2: KU 334063; MO 6: KU 334059–70; AN 3: CAS 133424–6, CAS 133506–13, CAS 133634–35, CAS 133662–63, CAS 133697, CAS 133708, CAS 139316–8, CAS 186129.

##### *Cyrtodactylus annulatus* (Taylor, 1915)

**Figure 47.**
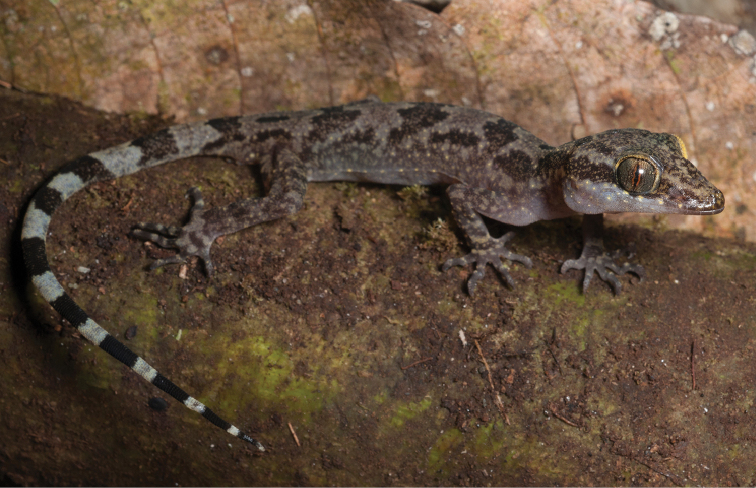
*Cyrtodactylus annulatus* female (KU 334025) from Eye Falls, Mt. Hilong-hilong, Municipality of Remedios T. Romualdez, Agusan del Norte Province. Photo: RMB.

Occurring in sympatry with *Cyrtodactylus agusanensis*, *Cyrtodactylus annulatus* (Fig. 47) can be identified by its smaller size and lower non-overlapping range of paravertebral scales (Brown and Alcala 1978; Welton et al. 2009). *Cyrtodactylus annulatus* has been reported from a number of localities across the archipelago including Camiguin Sur Island and in the northeastern regions of Mindanao Island. We frequently encountered these geckos on vegetation in the forest at night. This species is currently listed as “Least Concern” (LC; IUCN 2016).

Sites and specimens: AN 10: KU 334037; AN 11: KU 334022–33, KU 334035; AN 12: KU 334041–43, KU 334053; AS 2: USNM 229373; AS 5: KU 320008–13; AS 6: KU 314944–46; C 14: KU 309363–66; MO 6: KU 334055–58, KU 334060, KU 334066–67; AN 1: CAS 133694, CAS 133760–61; AN 3: CAS 133556–57, CAS 133574, CAS 133783–84; C 1: CAS-SUR 28230–28, CAS-SUR 28390–96, CAS-SUR 28398–406; C 2: CAS-SUR 28397, CAS-SUR 28407; C 6: Sites and specimens: CAS 137542, CAS 139032, CAS 208601–02, CAS-SUR 26204, CAS-SUR 26211–13, CAS-SUR 26224, CAS-SUR 26227, CAS-SUR 26237, CAS-SUR 26303, CAS-SUR 26311, CAS-SUR 26319; C 7: CAS 137543–50, CAS 139033, CAS 185740–41, CAS 186413, CAS-SUR 26200–03, CAS-SUR 26240, CAS-SUR 26242, CAS-SUR 26307, CAS-SUR 26312, CAS-SUR 26315; C 8: CAS-SUR 26217–18, CAS-SUR 26238, CAS-SUR 26243, CAS-SUR 26304; SN 1: CAS 131838.

##### *Cyrtodactylus mamanwa* Welton, Siler, Linkem, Diesmos & Brown, 2010

**Figure 48.**
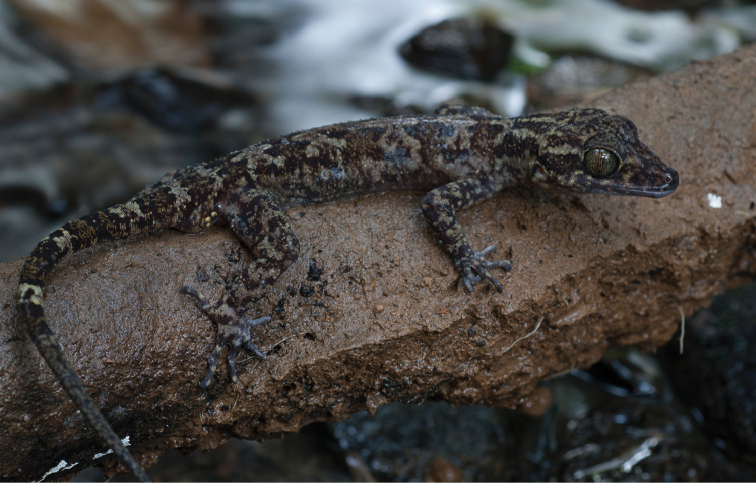
*Cyrtodactylus mamanwa* male (KU 305564) from Municipality of Loreto, Dinagat Island. Photo: CDS.

Formerly recognized as part of the *Cyrtodactylus agusanensis* Complex, the Dinagat Island clade is now referred to as a unique Philippine endemic species, *Cyrtodactylus mamanwa* (Fig. 48), as a result of an analysis of molecular and morphological evidence and a comprehensive taxonomic review (Welton et al. 2010). This species was collected among large boulders and logs on the bank of a stream at night. Future surveys may reveal additional populations on Siargao Island. This species does not qualify for any elevated threat categories and we classify it as “Least Concern” (LC; IUCN 2010).

Sites and specimens: D 2: KU 305564–65 (paratypes); D 5: KU 310094–108 (paratypes), 310109 (holotype), 310110–12 (paratypes).

##### *Gehyra mutilata* (Wiegmann, 1834)

This species of house gecko is quite common throughout the Philippines, and regularly encountered in residential habitats (in poorly lighted areas, in contrast to most house geckos such as *Hemidactylus platyurus* and *Hemidactylus frenatus*, which are most frequently encountered below lights) mixed and disturbed forest edge habitats. To date, no phylogenetic studies have focused on understanding population genetic structure or patterns among the widespread species of house geckos in the Philippines (*Gehyra mutilata*, *Hemidactylus platyurus*, or *Hemidactylus frenatus*). Owing to its widespread distribution, this species does not qualify for any elevated threat categories and is classified as “Least Concern” (LC; IUCN 2010).

Sites and specimens: C 10: KU 302653–54; MO 6: KU 334018; C 1: CAS-SUR 28204, CAS-SUR 28379; C 6: CAS-SUR 26206, CAS-SUR 26210, CAS-SUR 26215, CAS-SUR 26222, CAS-SUR 26226, CAS-SUR 26300–01, CAS-SUR 26321.

##### Gekko cf. mindorensis

**Figure 49.**
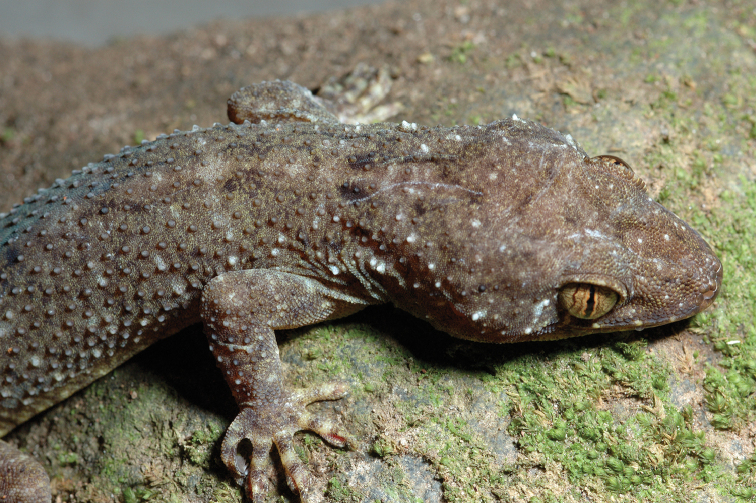
Gekko cf. mindorensis (specimen not collected) from Municipality of Mambajao, Mt. Mambajao, Camiguin Sur Island. Photo: CDS.

A recent phylogeographic (Siler et al. 2012c, 2014b) investigation identified eight genetically divergent and geographically structured clades within this Philippine endemic species, confirming previous speculation (Ferner et al. 2001, Brown et al. 2013) that many Philippine populations constitute a cryptic species complex. In a follow-up study, Siler et al. (2014b) recognized *Gekko kikuchii* as a member of the *Gekko mindorensis* Complex, with a distribution that included populations from the islands of Luzon (northern Philippines) and Lanyu (Taiwan). Because these changes render *Gekko mindorensis* paraphyletic, Siler et al. (2014b) suggest the recognition of *Gekko mindorensis* as a monophyletic clade that includes populations from the Mindoro PAIC, the Bicol Peninsula and Catanduanes Island of the Luzon PAIC, the Visayan PAIC, and Bohol and Camiguin Sur islands of the Mindanao PAIC. Following these studies, we recognize populations of Camiguin Sur (Fig. 49) and Panglao as Gekko cf. mindorensis in anticipation of taxonomic resolution of this complex and diverse group of Philippine endemic geckos. *Gekko mindorensis* is classified as “Least Concern” (LC; IUCN 2016) but should now be considered “Data Deficient” (DD; IUCN 2010) until a taxonomic assessment of species diversity can be performed.

Sites and specimens: C 10: KU 302683–86.

##### *Gekko gecko* (Linnaeus, 1758)

This species occurs throughout Southeast Asia and has been recorded at most well surveyed sites throughout the Philippines with the exception of the Batanes and Babuyan Island Groups (Brown and Alcala 1970, 1978; Siler et al. 2012). We frequently observed this species on manmade structures, in well-lighted areas (Brown et al. 2012a, 2013a) or in nearby trees such as plantations. Its conservation status is “Least Concern” (LC; IUCN 2016).

Sites and specimens: C 12: KU 304145–46; C 15: KU 309880–82; KU 310360; MO 3: KU 334015–16.

##### *Gekko monarchus* (Shlegel, 1836)

*Gekko monarchus* although reported from a few localities in the oceanic portions of the Philippines (Taylor 1922a; Brown and Alcala 1978) has most frequently and convincingly been reported from Palawan. Recent molecular analyses of *Gekko* samples from throughout the country (Siler et al. 2012, 2014b) have failed to find true *Gekko monarchus* at any Philippine localities beyond Palawan, leading us to consider that the species may be extremely rare on the Mindanao PAIC or may be attributed to the oceanic (non-Palawan) portion of the archipelago in error.

Sites and specimens: C 6: CAS-SUR 26299; SS 3: CAS 15556–57.

##### *Hemidactylus frenatus* (Duméril & Bibron, 1836)

We observed this human commensal species in nearly every site we have surveyed in northeast Mindanao and off shore islands. Considered “Least Concern” (LC; IUCN 2016) because of its constant presence in human habitations, this species frequently has been discounted in faunal surveys and is, as a result, poorly studied.

Sites and specimens: AS 2: USNM 229374–81; C 14: KU 309883–5; D 1: USNM 229320–21; D 2: KU 305759–65; D 2: KU 305767–72; D 4: KU 305766; MO 7: KU 326553; C 6: CAS-SUR 26225.

##### *Hemidactylus platyurus* (Schneider, 1792)

Like its congener, *Hemidactylus platyurus* is understudied and often overlooked in faunal inventories because it is a constant presence in and around human habitations. Although it may warrant its current IUCN conservation status (“Least Concern”; IUCN 2016), this categorization remains to be critically evaluated.

Sites and specimens: AS 2: USNM 229371–72; D 4: KU 305509–10; SS 3: CAS 15558.

##### Hemiphyllodactylus cf. typus Bleeker, 1860

In response to Zug’s (2010) consideration of *Hemiphyllodactylus typus* as a widespread, “low diversity” and invariant taxon, Grismer et al. (2013) showed deep phylogenetic structure within Hemiphyllodactylus “typus” suggesting the existence of many distinct species, several of which subsequently have been named. The taxonomic status of the Mindanao population(s) have not yet been studied, so we refer to them as Hemiphyllodactylus cf. typus in hopes that a thorough review of their status (at multiple sites throughout the island) will soon be undertaken. The conservation status of *Hemiphyllodactylus typus* formally is “Least Concern” (LC; IUCN 2016) although pending taxonomic revision may show this species to be much more range restricted. Similarly, Philippine populations most likely will be partitioned into a number of species (Grismer et al. 2013) suggesting that like *Gekko mindorensis*, all *Hemiphyllodactylus* populations in the Philippines should now be considered “Data Deficient” (DD; IUCN 2010) until taxonomic reassessment in available.

Sites and specimens: AS 6: KU 314090–91.

##### *Lepidodactylus aureolineatus* Taylor, 1915

This Mindanao PAIC endemic is a species that historically has been heavily collected (possibly indicating its stable and widespread population status; Brown and Alcala 1978) but which has been conspicuously absent in recent surveys. Considered “Least Concern” (LC; IUCN 2016), this species may be common at low elevations and thus, was frequently encountered in late 1900s surveys, but absent in recent studies targeting montane habitats. A focused investigation of its distribution and taxonomic status would be highly desired.

Sites and specimens: C 6: USNM 198148; AN 3: CAS 139941, CAS 156687; C 1: CAS-SUR 28203, CAS-SUR 28387, CAS-SUR 28389; C 2: CAS-SUR 28201–02, CAS-SUR 28388, CAS-SUR 28985–86; C 5: CAS-SUR 26207–09, CAS-SUR 26316; C 6: CAS 191257–63, CAS-SUR 26128, CAS-SUR 26139, CAS-SUR 26196–98, CAS-SUR 26205, CAS-SUR 26214, CAS-SUR 26216, CAS-SUR 26219–21, CAS-SUR 26223, CAS-SUR 26302, CAS-SUR 26305–06, CAS-SUR 26309–10, CAS-SUR 26313–14, CAS-SUR 26317–18, CAS-SUR 26320; C 7: CAS 141404–19, CAS-SUR 26127.

##### *Lepidodactylus labialis* (Peters, 1864)

This previously considered common and “Least Concern” (LC; IUCN 2016) species has not been recorded in any of our recent surveys, even those focused near the type locality (Mt. Hilong-hilong. Siler et al. (2014c) demonstrated that this species had previously been incorrectly placed in the genus *Pseudogekko* (Brown and Alcala 1978), and actually possesses diagnostic characters that ally it with *Lepidodactylus*. This species has not been observed since its original collection and should now be designated “Data Deficient” (DD; IUCN 2010) until an assessment in the form of a field survey at the type locality can be undertaken. It is possible that *Lepidodactylus labialis* could be a range-restricted microendemic species and a major conservation concern.

Sites and specimens: AN 1: CAS 133687; AN 3: CAS 133396, CAS 133701, CAS 133790; AN 4: CAS 133150, CAS 133209–10, CAS 133238, CAS 133243, CAS 133258, CAS 133314–18, CAS 133329–30, CAS 133338–39, CAS 133353–6, CAS 139388–89; AN 5: USNM 496788; MO 6: KU 334019.

##### *Pseudogekko pungkaypinit* Siler et al., 2014

**Figure 50.**
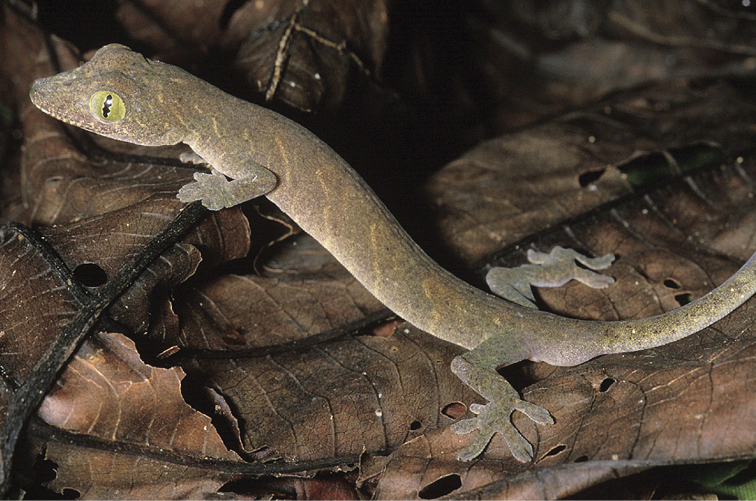
*Pseudogekko pungkaypinit* has been collected in Mt. Lumot, Municipality of Gingoog, Misamis Oriental Province. Photo: RMB (from the Municipality of Baybay, Leyte Island; KU 326243).

This rare forest-obligate species (Fig. 50) is a member of an endemic genus of geckos in the Philippines, and formerly recognized as a member of the *Pseudogekko compresicorpus* Complex (Brown and Alcala 1978, Siler et al. 2014c, d). Recently, the species complex was revised using genetic and morphological data, restricting the range of true *Pseudogekko compresicorpus* to the Luzon PAIC, and recognizing three new species within the species complex (Siler et al. 2014c,d). The longer-bodied species found in central and northeastern Mindanao is now recognized as *Pseudogekko pungkaypinit*, and at this time does not qualify for elevated or threatened conservation status (LC; Siler et al. 2014; IUCN 2016).

Sites and specimens: MO 6: KU 334019.

#### Family Scincidae

##### *Brachymeles vulcani* Siler, Jones, Diesmos, Diesmos & Brown, 2012

Formerly considered conspecific with *Brachymeles gracilis* (Taylor 1917a; Brown and Alcala 1980), the Camiguin Sur Island population was described as a distinct species (*Brachymeles vulcani*) by Siler et al. (2012). Most specimens of this species have been collected from under or inside dry rotting logs in contact with the forest floor, including secondary forests and coconut palm plantations adjacent to forested areas at low- to mid-elevations on Mt. Mambajao. Although quite common in earlier collections (Brown and Alcala 1980), Siler et al. (2012b) reported the collection of only a single specimen following three targeted herpetological expeditions to Camiguin Sur Island. Although the authors recommended against the use of negative data to inflate conservation urgency, Siler et al. (2012b) evaluated this species against IUCN (2010) criteria and elevated its conservation status to “Vulnerable” (VU; Siler et al. 2012b) based on restricted area of occurrence.

Sites and specimens: C 1: CAS-SUR 28314, CAS-SUR 28329, CAS-SUR 28331, CAS-SUR 28358–59; C 2: CAS-SUR 28199; C 6: CAS-SUR 26294, CAS-SUR 26236, CAS-SUR 26165–66, CAS-SUR 26184–86; C 7: CAS 138255, CAS 139030–31, CAS-SUR 26142, CAS-SUR 26144–46, CAS-SUR 26231; C 8: CAS-SUR 26295; C10: PNM 9766 (holotype; formerly KU 310359); C 14: KU 310359.

##### *Brachymeles tiboliorum* Siler, Jones, Diesmos, Diesmos & Brown, 2012

One juvenile of undetermined sex (KU 326109) collected in Initao National Park, Barangay Initao, Municipality of Tubigan, Misamis Oriental Province in May 2001 by ACD. This specimen was tentatively identified as *Brachymeles tiboliorum* during a recent review of the *Brachymeles gracilis* complex (Siler et al. 2012b). *Brachymeles tiboliorum* is otherwise restricted to southern Mindanao, which makes this single specimen record somewhat tentative. It is conceivable that, with the collection of additional specimens from northern Mindanao, this population may be recognized in future studies as a distinct species. Although this species has not yet been assessed (IUCN 2016) it is quite clear that it should be regarded as “Data Deficient” (DD; IUCN 2016)

Sites and specimens: MO: KU 326109.

##### *Brachymeles hilong* Brown & Rabor, 1967

**Figure 51.**
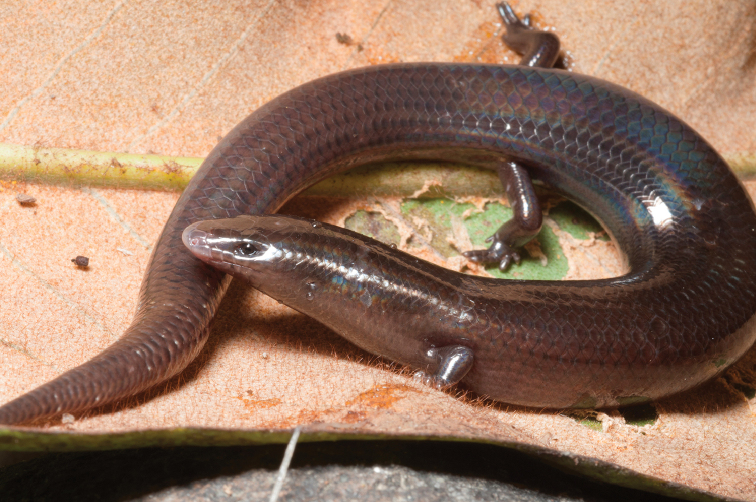
*Brachymeles hilong* male (KU 334282) photographed at the type locality, Mt. Hilong-hilong. This specimen was collected at Municipality of Remedios T. Romualdez, Agusan del Norte. Photo: RMB.

Like *Brachymeles orientalis*, this species (Fig. 51) of slender skink is quite common in Caraga Region of northeast Mindanao; it formerly was classified as a subspecies of the “widespread” *Brachymeles gracilis* group (Brown and Alcala 1980) and the subspecies has also been reported from Samar and Leyte islands (now recognized as *Brachymeles samad*) and Camiguin Sur Island (now recognized as *Brachymeles vulcani*; Siler et al. 2012b). However, following Siler et al.’s (2012b) taxonomic revision of the *Brachymeles gracilis* complex, the range of *Brachymeles hilong* has been restricted to northeastern Mindanao (type locality: Mt. Hilong-hilong), where it can often be found in sympatry with *Brachymeles orientalis* (Smith 1993a; Siler et al. 2012b). Individuals are often found in residential, disturbed, and secondary-growth forest at low elevations. They are commonly observed within rotting logs, in leaf litter, in the root networks of tree buttresses, as well as beneath rotting piles of coconuts (Siler et al. 2012b). The species has been found to qualify for a conservation status of “Near Threatened” (NT; IUCN 2016; Siler et al. 2012a,b).

Sites and specimens: AN 1: USNM 496790–99, CAS 208594–96, CAS 133691–93, CAS 133740, CAS 133743–48; AN 11: KU 334289–300; AN 2: CAS 102406; AN 3: CAS 133215, CAS 133577–85, CAS 133586, CAS 133609–14, CAS 133702–06, CAS 133785, CAS 152194; AS 5: KU 319920, KU 319922, KU 319926–27, KU 319934–40; MO 6: KU 334301–08; SN 2: KU 327339–40; SS 2: CAS-SUR 24315–16.

##### *Brachymeles orientalis* Brown & Rabor, 1967

**Figure 52.**
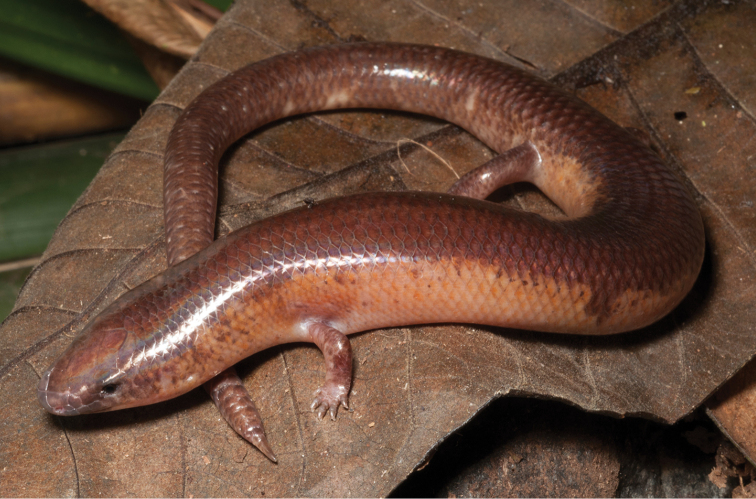
*Brachymeles orientalis* male (KU 334282) from Mt. Hilong-hilong in the Municipality of Remedios T. Romualdez, Agusan del Norte Province. Photo: RMB.

This common species of semi-fossorial slender skink (Fig. 52) can be found throughout the eastern and northern islands of the Mindanao PAIC, as well as Bohol, Leyte, and Camiguin Sur islands (Brown and Alcala 1980; Smith 1993a; Siler et al. 2011b) and Samar Island (specimens in KU). The species can often be found in sympatry with *Brachymeles hilong* on northeastern Mindanao, *Brachymeles samad* on Leyte and Samar islands, and *Brachymeles vulcani* on Camiguin Sur Island (Siler et al. 2012b). Individuals are often found in residential, disturbed, and secondary-growth forest at low elevations. They are commonly observed in soil and forest floor detritus around tree buttresses and under nearly any form of ground cover in coconut palm plantations adjacent to forest (Siler et al. 2010). Because of its wide geographical distribution and tolerance of disturbance, this species does not qualify for any elevated threat categories and is classified here as “Least Concern” (LC; IUCN 2010).

Sites and specimens: AN 1: CAS 133749–54; AN 3: CAS 133616; AN 4: CAS 133301; AN 5: USNM 496800; AN 9: KU 327126–28; AN 11: KU 334275–80; AN 12: KU 334281–86, KU 334405; AS 2: USNM 229382–83; AS 5: KU 319921, KU 319923–25, KU 319928–31; AS 6: KU 314092–96; C 1: CAS-SUR 28315–28, CAS-SUR 28330, CAS-SUR 28332, CAS-SUR 28333, CAS-SUR 28336, CAS-SUR 28338, CAS-SUR 28340, CAS-SUR 28342–45, CAS-SUR 28350, CAS-SUR 28353–57, CAS-SUR 28360–72, CAS 110976–83; C 7: CAS-SUR 26141, CAS-SUR 26188, CAS-SUR 26234; C 8: CAS-SUR 26228–29; C 13: KU 327129–33; C 14: KU 310357–58; D 4: KU 305469; MO 2: KU 334287–88; MO 5: KU 319948.

##### *Eutropis multicarinata* (Gray, 1845)

**Figure 53.**
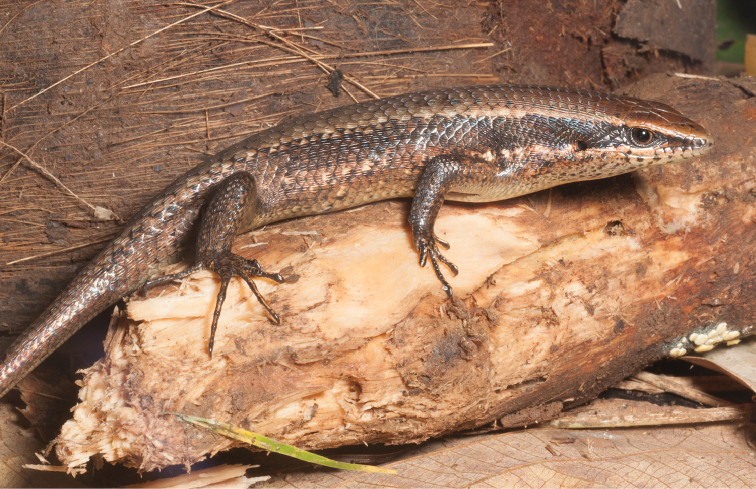
*Eutropis multicarinata* female (KU 334227) from Mt. Lumot, Municipality of Gingoog City, Misamis Oriental Province. Photo: RMB.

The southern Philippine common sunskink traditionally known as *Eutropis multicarinata multicarinata* (Fig. 53) has been documented on most of the islands of the Mindanao PAIC (Brown and Alcala 1980; Smith 1993a). Although the type locality of this species was designated simply “The Philippines,” Barley et al. (2013) matched the type material phenotype to populations from Mindanao, Leyte, and Dinagat islands. Two additional lineages in the large *Eutropis multicarinata* Group clade were identified in the southern Philippines: one from southwest Mindanao (“Clade A;” Barley et al. 2013) and another from Mindanao, Bohol, Dinagat and Siargao (“Clade E;” Barley et al. 2013. Although the type locality of this species was designated simply AS 2: USNM 2293 of associated genetic samples in older collections) under the name *Eutropis multicarinata* pending a taxonomic revision that will be published elsewhere (Barley et al. *unpublished data*). Given the taxonomic uncertainty and morphologically cryptic nature of these populations, this taxon must be considered “Data Deficient” (DD; IUCN 2016).

Sites and specimens: AN 3: CAS 133267–72, CAS 133370, CAS 133428, CAS 133485, CAS 133624; AN 4: CAS 133138–39; KU 314097–98; AN 5: USNM 497027; AS 2: USNM 229384–85; AS 4: KU 314105–07; AS 5: KU 320025–29; C 1: CAS-SUR 28261–62, CAS-SUR 28347; C 2: CAS-SUR 28217, CAS-SUR 28263–64; C 7: CAS 139037; D 2: KU 306194; D 4: KU 306195; MO 5: KU 320030.

##### Eutropis cf. multicarinata

Barley et al. (2013) demonstrated the genetic distinctiveness of some individuals of “*Eutropis multicarinata*” at selected populations on Mindanao, Bohol, and Dinagat (and possibly and Siargao) islands (“Clade E;” Barley et al. 2013). Our specimens from Mt. Lumot (Misamis Oriental Province), Mt. Magdiwata, Bunawan (Agusan del Sur Province), and Loreto, Dinagat Island have been genetically identified as members of this highly divergent clade, which we anticipate will eventually be recognized as a distinct species.

Sites and specimens: AS 1: KU 314098; AS 5: KU 320028; KU MO 6: KU 334225–30; D 2: KU 310152, 310154, 310156; D 4: 314105, 314106.

##### Eutropis cf. indeprensa (Brown & Alcala, 1980)

This relatively common Philippine sunskink has been reported as occurring throughout the southern portion of the archipelago (Brown and Alcala 1980), including Palawan and even northern Borneo. Some populations from the northern portion of the archipelago have been described as a distinct, but closely related species, *Eutropis cumingi*. When naming *Eutropis indeprensa*, Brown and Alcala (1980) restricted the type locality to Mindoro, which made clear Barley et al.’s (2013) assignment of the name-bearing types to a definite population on Mindoro (Barley at al. 2013). Brown and Alcala (1980) reported the species from Mindanao, Leyte, Samar, “Camiguin” (presumably Camiguin Sur Island), Palawan, Negros and Cebu, but Barley et al. (2013), using a multilocus molecular analysis showed that these populations do not form a monophyletic clade, and that this species is restricted to Mindoro Island and Borneo—a highly unusual and disjunct distribution if true. Barley et al.’s (2013) highly divergent “Clade C” (which is not closely related to true, type-locality *Eutropis indeprensa*) contained samples from Dinagat, Mindanao, Samar, Panay, Cebu, and the Bicol Peninsula of Luzon (see Barley et al. 2013: Figure 3) and we find it likely that these eventually will be recognized as a distinct species. *Eutropis indeprensa* species has not been assessed for conservation status but may qualify for an elevated level of threat should additional studies confirm a Mindoro-only distribution in the Philippines (IUCN 2010); for now it is best considered “Data Deficient” (DD; IUCN 2010).

Sites and specimens: AS 6: KU 314104; D 4: KU 310156; D 5: KU 310149–55; C 1: CAS-SUR 28218–19, CAS-SUR 28265, CAS-SUR 28270, CAS-SUR 28337, CAS-SUR 28351; C 6: CAS-SUR 26172.

##### *Eutropis multifasciata* (Kuhl, 1820)

**Figure 54.**
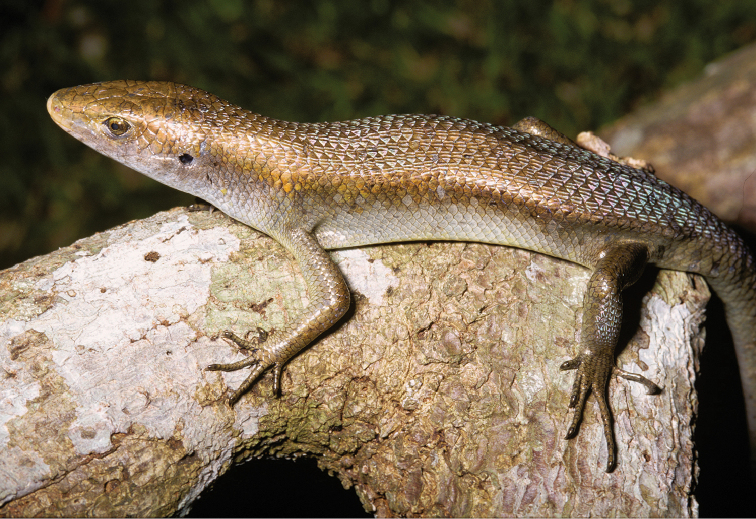
*Eutropis multifasciata* male (KU 334403) from Mt. Hilong-hilong in the Municipality of Remedios T. Romualdez, Agusan del Norte Province. Photo: RMB.

This geographically widespread habitat generalist species (Brown and Alcala 1980; Grismer 2011) has been recorded and observed throughout the Philippines, most of the western landmasses of the Indo-Australian archipelago, southwest Asian and northward into Indochina. Related to *Eutropis grandis* (syn. *Eutropis macropthalma*) and *Eutropis rudis* (Barley et al. 2014), this species (Fig. 54) clearly possesses the dispersal capacity to overcome marine barriers to colonization and yet, at a much finer scale, has been shown to possess the genomic signature of habitat fragmentation in the Philippines (Barley et al. 2015). A truly widespread species, *Eutropis multifasciata* does not qualify for any elevated level of threat and must be considered “Least Concern” (DD; IUCN 2016).

Sites and specimens: AN 10: KU 334403; AS 4: KU 314100–03; AS 6: KU 314099; C 15: KU 309886; D 5: KU 310371–72; C 1: CAS-SUR 28346; C 6: CAS-SUR 26129, CAS-SUR 26133–34, CAS-SUR 26156, CAS-SUR 26158, CAS-SUR 26195.

##### *Lamprolepis smaragdina philippinica* (Mertens, 1928)

**Figure 55.**
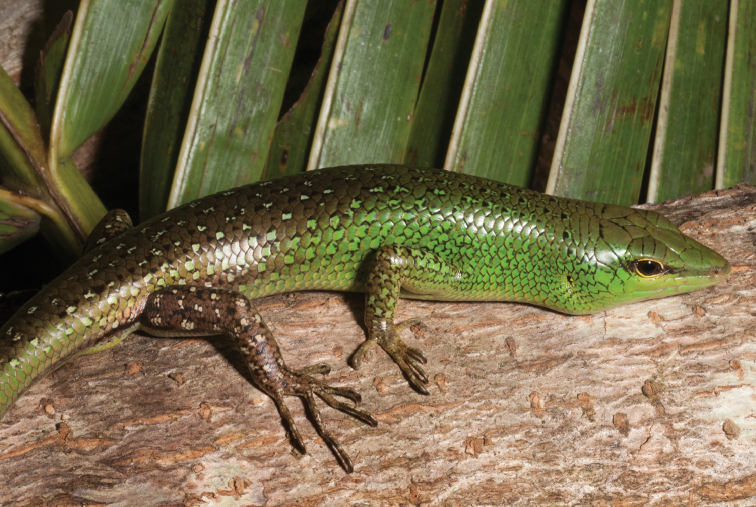
*Lamprolepis smaragdina philippinica* is common throughout coastal areas of northeast Mindanao. Photo: RMB (from the Municipality of Burauen, Leyte Island; specimen deposited at KU).

This species is widely distributed throughout the archipelago and locally abundant in coastal and agricultural areas (Brown and Alcala 1980; Smith 1993a). We typically encountered *Lamprolepis smaragdina* (Fig. 55) on coconut trees in plantations, as well as developed areas in close proximity to agricultural areas. Due to its tolerance for human disturbance and its ubiquitous distribution, this species is considered “Least Concern” (LC; IUCN 2016). A recent phylogeographic study (Linkem et al. 2013) identified the presence of numerous highly divergent evolutionary lineages in the Philippines, suggesting that reassessment of conservation status should be attempted once taxonomic reconsideration of this group has been undertaken.

Sites and specimens: AN 15: KU 334240–43. AS 4: KU 314108–09; C 1: CAS-SUR 28196; C 11: KU 309888–97; C 12: KU 302857–59; C 13: KU 309887; C 6: CAS-SUR 26136, CAS-SUR 26170, CAS-SUR 26296; D 2: KU 305932–59; D 4: KU 305938; SN 1: CAS 60583–84.

##### *Lipinia auriculata herrei* (Taylor, 1922)

Taylor (1917b) described the species *Siaphos* (currently *Lipinia*) *auriculata* on the basis of a few specimens from northern Negros and he later described *Lipinia kempi* from Mindoro (Taylor 1919) and *Lipinia herrei* from Polillo Island (Taylor 1922a). At the time of their review of Philippine scincid lizards, Brown and Alcala (1980) remarked on the close phenotypic similarity of the original specimens, and therefore considered them to be closely related and recognizable only at the level of subspecies (Brown and Alcala 1980). Interestingly, although few rare forest Philippine reptile taxa had been recorded to possess wide ranges encompassing the Luzon, Mindanao and Mindoro faunal regions (Taylor 1928; Leviton 1963; Brown and Alcala 1970), Brown and Alcala (1980) assigned one specimen from Bohol and two specimens from northeast Mindanao to the subspecies *Lipinia auriculata herrei* on the basis of a single character in common: their shared, distinctly paired (i.e., unfused) frontoparietal scales. Remarking on this oddly disjunct distribution, the authors did however explicitly state that they anticipated the species would eventually be recorded at intervening localities on the islands of Leyte and Samar (Brown and Alcala 1980:90). *Lipinia auriculata* has been classified by the IUCN (2016) to be of “Least Concern.”

Sites and specimens: AN 3: CAS 133700, CAS 133778.

##### *Lipinia pulchella pulchella* Gray, 1845

**Figure 56.**
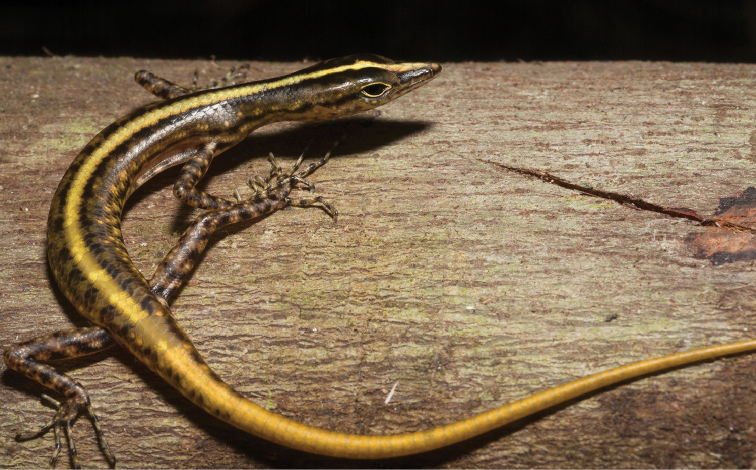
*Lipinia pulchella pulchella* has been recorded in Agusan del Norte and Agusan del Sur Provinces. Photo: RMB (from the Municipality of Burauen, Leyte Island; specimen deposited at KU).

Gray (1845) described *Lipinia pulchella* (the type species for the genus; Fig. 56) on the basis of a single specimen from Mindanao Island; Taylor (1917b) subsequently confirmed the species on Negros Island (= *Lipinia pulchella taylori* Brown and Alcala 1956) and Brown and Alcala (1980) used large samples from throughout the archipelago to define three subspecies: *Lipinia pulchella pulchella* (from Mindanao, Leyte, Samar, southern Luzon and Polillo islands), *Lipinia pulchella taylori* (Negros Island), and *Lipinia pulchella levtoni* (northern Luzon). The various populations of *Lipinia pulchella* are all arboreal and specimens are most often encountered climbing on tree bark, out on the ends of tree branches, or hidden within epiphytic vegetation. Despite the paucity of recent information, the wide geographic range of the *Lipinia pulchella* Complex renders it “Least Concern” (LC; IUCN 2016).

Sites and specimens: AN 1: CAS 133688–89, CAS 133730–33, CAS 133757–58; AN 3: CAS 133563–64, CAS 133625, CAS 133786, CAS 154715; AS 4: KU 314121–23; AS 5: KU 319899–902; AS 6: KU 314110–20; D 5: KU 310139–48.

##### *Lipinia quadrivittata* (Peters, 1867)

Peters (1867) described “*Lygosoma*” (=*Lipinia*) *quadrivittata* from three Mindanao Island specimens, and the species subsequently has been reported from Bohol, Leyte, Negros, Palawan, Siquijor, Cebu and Camiguin Sur islands (Taylor 1922a; Brown and Alcala 1980). The Mindanao specimens with habitat data were collected from under the moss on tree trunks at 800+ m above sea level and one was collected from an arboreal fern (Brown and Alcala 1980). This species has not been assessed for conservation status (IUCN 2016) and we consider it “Data Deficient” (DD; IUCN 2016) given the absence of any other information on its distribution and status. Although *Lipinia quadrivittata* appears to have a wide distribution on numerous Philippine islands, we hesitate to classify it as “Least Concern” on the arbitrary basis of distribution alone. On Negros, Brown and Alcala (1980) noted that this species was predictably observed on trees in swamps. Given that swamp ecosystems are severely imperiled throughout the archipelago, plus the fact that this habitat is quite different than that noted for Mindanao (montae forest), we suspect that more *Lipinia quadrivittata*, as currently conceived, may be a complex of more than one species. The species’ anomalous distribution pattern (three Philippine faunal regions + Borneo) reinforces our suspicion that more than one species is involved. *Lipinia quadrivittata* should be the subject of a taxonomic review, which should immediately be followed by conservation status assessment.

Sites and specimens: AS 6: KU 314124–25; 6: CAS-SUR 26161–62, CAS-SUR 26175–76, CAS-SUR 26244, CAS-SUR 26290

##### *Otosaurus cumingi* Gray, 1845

*Otosaurus cumingi*, the largest Philippine *Sphenomorphus*-Group lizard of the family Scincidae is a monotypic taxon, which was recently elevated to the level of a distinct genus (previous recognized as a member of the genus *Sphenomorphus*; Brown and Alcala 1980) by Linkem et al. (2011). Not closely related to other Philippine *Sphenomorphus*-Group lizards, this large-bodied taxon is part of an unresolved polytomy involving the genus *Otosaurus* the Philippine clade, and the Australian clade (Linkem et al. 2011). The single species of *Otosaurus* (Philippine *Otosaurus cumingi*) is widespread throughout the archipelago, except Palawan (Taylor 1922a; Brown et al. 1980; Brown et al. 2012a, 2013). This widespread species is considered "Least Concern" (LC; IUCN 2010).

Sites and specimens: AS 5: KU 319961.

##### *Parvoscincus kitangladensis* (Brown, 1995)

**Figure 57.**
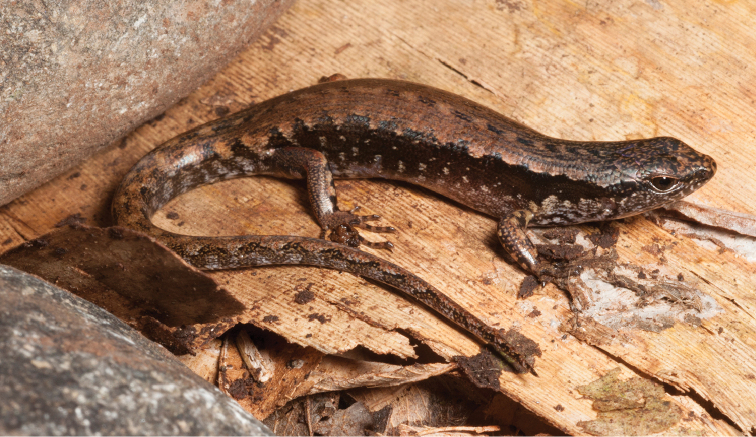
*Parvoscincus kitangladensis* male (KU 334078) from Eye Falls, Mt. Hilong-hilong, Municipality of Remedios T. Romualdez, Agusan del Norte Province. Photo: RMB.

Described from high elevation forested plateaus of Bukidnon Province, central Mindanao (Brown 1995), this small forest species (Fig. 57) is now also known from eastern and northeastern Mindanao. *Parvoscincus kitangladensis* is most closely related to the miniature Luzon forest species (e.g., *Parvoscincus lawtoni*, and possibly *Parvoscincus luzonensis*) but is phenotypically similar to members of the *Parvoscincus decipiens* complex and other small bodied taxa from Luzon (i.e. *Parvoscincus laterimaculatus*, *Parvoscincus leucospilos*; Linkem et al. 2011; Siler et al. 2014e) and even Palawan (e.g., *Insulasaurus victoria*, *Insulasaurus traanorum*; Linkem et al. 2010a, 2011). Considered “Least Concern” (LC; IUCN 2010, 2016), this species is reasonably widespread but remains poorly understood in microhabitat preferences.

Sites and specimens: AN 1: CAS 133695, CAS 133762–65; AN 11: KU 334078–82; AN 12: KU 334083–85; AN 3: CAS 133273–79, CAS 133371–76, CAS 133412–20, CAS 133459–63, CAS 133471–73, CAS 147273–76, USNM 496804; AN 4: CAS 133235, CAS 133294; AN 5: USNM 496803, USNM 497029; AS 5: KU 326067; MO 3: KU 334244–46; MO 5: KU 326613–22; SS 2: CAS-SUR 24207.

##### *Parvoscincus steerei* (Stejneger, 1908)

Members of the large, geographically structured, genetically diverse taxon *Parvoscincus steerei* are widespread throughout the oceanic islands of the Philippines (Brown and Alcala 1980) and exhibit some color variation, but very little variability in external morphology (scalation; Linkem et al. 2011; RMB *personal observation*). Frequently encountered from disturbed second growth and agro-ecosystem matrices, this species extends up to mid-elevation original forests (700–900 m) and is common in leaf litter, under woody forest debris, and in forest detritus surrounding roots and buttresses of trees. This widespread species, as presently conceived, is considered “Least Concern” (LC; IUCN 2010, 2016) but genetic evidence suggest that *Parvoscincus steerei* is a species complex composed of numerous evolutionary lineages; these may warrant individual conservation assessment with future investigation.

Sites and specimens: AN 11: KU 334072–75; AS 6: KU 314126–27; MO 2: KU 334076–77; MO 5: KU 326628–31; SN 2: KU 326632; AN 1: CAS 133736–37; AN 3: CAS 133407–08, CAS 133575; AN 4: CAS 133141–44, CAS 133240–41, CAS 133255–56, CAS 133320–21, CAS 133325–27, CAS 133362, USNM 496806; C 1: CAS-SUR 28207–09, CAS-SUR 28247; C 6: CAS-SUR 26131, CAS-SUR 26150–51, CAS-SUR 26160, CAS-SUR 26173–74, CAS-SUR 26241, CAS-SUR 26288, CAS-SUR 26289, CAS-SUR 26291.

##### *Pinoyscincus abdictus abdictus* (Brown & Alcala, 1980)

**Figure 58.**
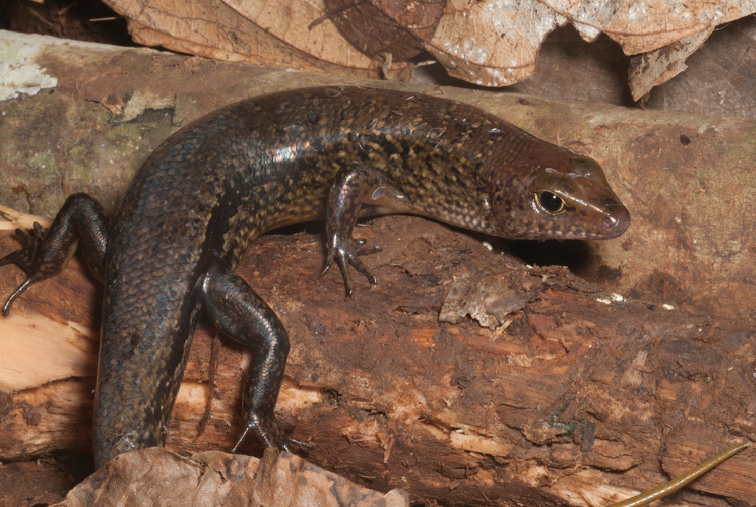
*Pinoyscincus abdictus abdictus* (KU 334268) from Mt. Hilong-hilong, Municipality of Remedios T. Romualdez, Agusan del Norte Province. Photo: RMB.

Originally described from Dinagat Island (Fig. 1), this northeast Mindanao faunal region taxon (Fig. 58) was reported to be distinguishable from the Luzon faunal region subspecies *Pinoyscincus abdictus aquilonius* on the basis of slight scalation differences and color pattern (Brown and Alcala 1980). In a recent multi-locus phylogenetic analysis, Linkem et al. (2011) demonstrated that this population was more closely related to *Pinoyscincus coxi*, and *Pinoyscincus llanosi* than it was to its putative closely-related subspecies, a finding that reinforced an earlier mitochondrial DNA analysis (Linkem et al. 2010b). This species is frequently encountered in a wide variety of habitats, including second growth forests abutting agricultural areas, riparian areas, and interior mature and second growth montane forests. This common species is considered “Least Concern” (LC; IUCN 2010, 2016).

Sites and specimens: AN 1: CAS 133755; AN 10: KU 334267–9; AN 11: KU 334265–6. AN 3: CAS 133615; AS 5: KU 321819–30. C 1: CAS-SUR 28341; C 14: KU 309911. C 6: CAS 137564, CAS-SUR 26183, CAS-SUR 26194; D 2: KU 306539–41; D 4: KU 306535–8; D 4: KU 306542–44; D 5: KU 310133–34; MO 6: KU 334270–74. SS 2: CAS 185979.

##### *Pinoyscincus coxi coxi* (Taylor, 1915)

Originally described from central-western Mindanao, *Pinoyscincus coxi coxi* was distinguished from S. Luzon and Mindoro Island populations of *Pinoyscincus coxi divergens* by slight differences in scalation and color pattern (Brown and Alcala 1980) but was shown in recent phylogenetic studies to be more closely related to *Pinoyscincus abdictus abdictus* and *Pinoyscincus llanosi* (Linkem et al. 2011). *Pinoyscincus coxi divergens*, in contrast, appears most closely related to *Pinoyscincus abdictus aquilonius* from neighboring geographic regions of Luzon (Linkem et al. 2010b). This species is a low- to mid-elevation forest edge generalist (Smith 1993a; RMB *personal observation*). This common species is considered “Least Concern” (LC; IUCN 2010, 2016).

Sites and specimens: AN 1: KU 326572–74; AN 12: KU 334234–5, KU 334237–9; AN 14: KU 334236; AN 3: USNM 204794; AS 1: CAS 62044–6; AS 5: KU 320412–4; C 1: CAS-SUR 28214–5, CAS-SUR 28241; C 14: KU 309908–10; C 2: CAS-SUR 28216; C 6: CAS-SUR 26159, CAS-SUR 26180–1, CAS-SUR 26235; C 7: CAS-SUR 26143, CAS-SUR 26148, CAS-SUR 26153, CAS-SUR 26155, CAS-SUR 26177, CAS-SUR 26191–3, CAS-SUR 26232–3; MO 1: KU 327676; SS 2: CAS 134076; AN 1: CAS 133742, CAS 133766, AN 3: CAS 133280–1, CAS 133458, CAS 133495, CAS 133587–9, CAS 133591–95, CAS 133618–20, CAS 133707, CAS 133789, CAS 152193; AN 4: CAS 133133, CAS 133234, CAS 133236, CAS 133292, CAS 133319, CAS 133328, CAS 133331–41.

##### *Pinoyscincus jagori jagori* (Peters, 1864)

Unlike the taxonomically confused and highly polyphyletic “species” *Pinoyscincus abdictus* and *Pinoyscincus coxi*, *Pinoyscincus jagori* fall into a single monophyletic clade (Linkem et al. 2010b, 2011). Known from eastern Mindanao, Dinagat and Siargao, populations keying out to *Pinoyscincus jagori* constitute four distinct, genetically divergent lineages (Linkem et al. 2010b, 2011): three of these, are identified as *Pinoyscincus jagori jagori* but nested within them is the population identified as *Pinoyscincus jagori grandis* (from the western Visayan islands; Brown and Alcala 1980). These large bodied skinks are common throughout disturbed and forested areas from near sea level to 500 or 600 m (RMB *personal observation*). This common species is considered “Least Concern” (LC; IUCN 2010, 2016).

Sites and specimens: AN 10: KU 334230–31; AN 9: KU 327645; AS 5: KU 321818; D 4: KU 306545–47; D 5: KU 310373.

##### *Pinoyscincus mindanensis* (Taylor, 1915)

**Figure 59.**
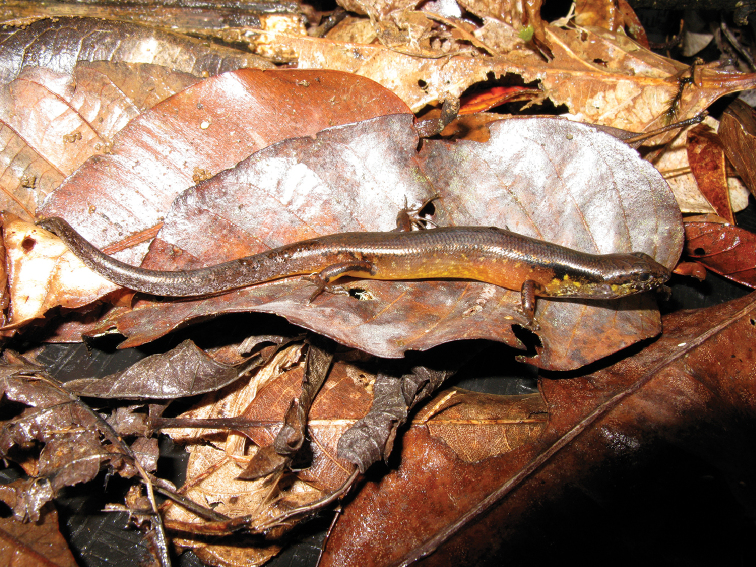
*Parvoscincus mindanensis* (specimen not collected) Mt. Balatukan, Municipality of Gingoog, Misamis Oriental Province. Photo: ACD.

This unusual small-bodied species (Fig. 59) was recently placed in the new genus *Pinoyscincus* (Linkem et al. 2011) together with former members of the *Sphenomorphus abdictus-jagori-coxi* assemblage (Taylor 1922a; Brown and Alcala 1980), in recognition of its close relationship with this large clade including numerous species, named subspecies, and morphologically cryptic but genetically distinct lineages (Linkem et al. 2010b). Unlike most other members of this group, *Pinoyscincus mindanensis* is a small bodied, montane forest species, whereas other taxa in this genus are large-bodied habitat generalists most frequently encountered at lower elevations (RMB *personal observation*). The other outlier in this group is *Pinoyscincus llanosi*, a medium-sized, low elevation riparian habitat specialist most frequently encountered on Samar and Leyte islands (but possibly present in northeast Mindanao) that is unique in that it retreats into running water when disturbed. Previously considered “Near Threatened” (NT; IUCN 2010), this species’ conservation status assessment is outdated and in need of reconsideration given the new distributional data presented here.

Sites and specimens: AN 11: KU 334247; D 5: KU 310113–14, KU 310135–38; SN 2: KU 332625–28; AN 1: CAS 133741; AN 3: CAS 133381, CAS 133397, CAS 133497, CAS 133590, CAS 147274, USNM 497030; AN 4: CAS 133145, CAS 133291, CAS 133293, CAS 133322; AN 5: USNM 497031.

##### “*Sphenomorphus*” *acutus* (Peters, 1864)

An ecologically unique and morphologically highly distinctive species, “*Sphenomorhus acutus*” was placed in the large clade of Philippine skinks (genera *Pinoyscincus* and *Parvoscincus*), but with low support for its phylogenetic position (Linkem et al. 2011). In recognition of its uncertain phylogenetic affinities and its extremely distinctive morphology, the authors elected to leave this taxon and *Sphenomorphus diwata* (its sister species) *incertae sedis*, and thus temporarily remaining in *Sphenomorphus*, ﻿pending further investigation (Linkem et al. 2011). This species has most frequently been encountered in recent years during nocturnal surveys, in which it has been located asleep in suspended coils of dry wild banana and abaca leaves, hanging 1–2 m above the forest floor (in original forest; RMB *personal observations*). It has been reported active in arboreal substrates (understory tree branches) during the day (Brown and Alcala 1980) and has been observed retreating into running forest streams when pursued by field workers (RMB *personal communication* with J. Fernandez). Despite this species’ enigmatic ecology and natural history, its widespread distribution and generalist habitat characterization render it “Least Concern” (LC; IUCN 2010, 2016).

Sites and specimens: AN 5: USNM 497028; AS 5: KU 319962–64; AN 1: CAS 133756; AN 3: CAS 133496, CAS 134228–29; AN 4: CAS 133134.

##### “*Sphenomorphus*” *diwata* Brown & Rabor, 1967

Member of Brown and Alcala’s (1980) “Group II *Sphenomorphus*”, *Sphenomorphus diwata* is an unusual montane forest species that shares with Luzon’s *Parvoscincus beyeri* Group (e.g. *Parvoscincus beyeri*, *Parvoscincus hadros*, *Parvoscincus igorotorum*, *Parvoscincus boyingi*; Brown et al. 2010a) the distinction of extremely high paravertebral scale counts (Brown and Alcala 1980; Brown et al. 1995a, b; Linkem et al. 2011). Surprisingly not related to these taxa, *Sphenomorphus diwata* (along with its sister species *Sphenomorphus acutus*) has been left *incertae sedis* by Linkem et al. (2011) in recognition of its uncertain phylogenetic affinities. This species is listed by the IUCN (2016) as “Data Deficient” (DD; IUCN 2010).

Sites and specimens: AN 6: USNM 496805, CAS 133514–15, CAS 133541, SS 2: CAS-SUR 24178.

##### *Sphenomorphus fasciatus* (Gray, 1845)

**Figure 60.**
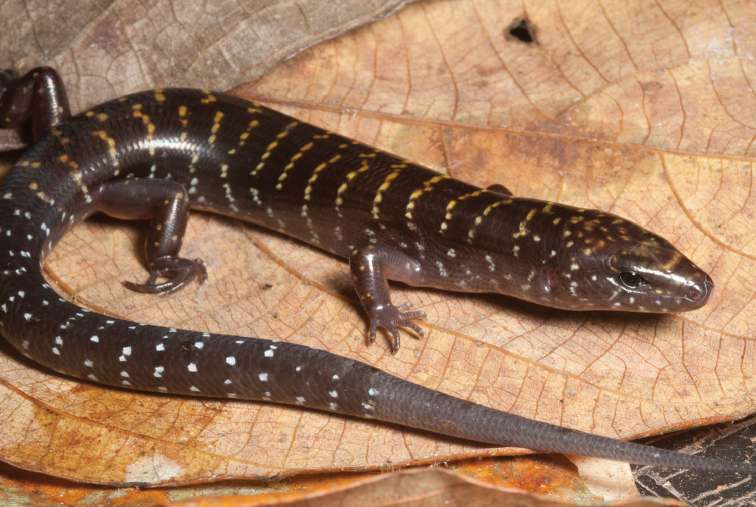
*Sphenomorphus fasciatus* (KU 334087) from Mt. Hilong-hilong, Municipality of Remedios T. Romualdez, Agusan del Norte Province. Photo: RMB.

Unrelated to other Philippine scincids, *Sphenomorphus fasciatus* (Fig. 60) is a morphologically distinctive species that is most closely related to Solomon Island taxa (e.g., *Sphenomorphus cranei*, *Sphenomorphus solomonis*, *Sphenomorphus concinatus*) and appears to be a single-species lineage that resulted in only one Philippine taxon (Linkem et al. 2011). Found throughout the islands of the Mindanao faunal region, this species is common in low elevation disturbed, second growth, and original coastal forests (Brown and Alcala 1980; Smith 1993a). This common species is considered “Least Concern” (LC; IUCN 2016).

Sites and specimens: AN 10: KU 334089–91; AN 11: KU 334087; AN 12: KU 334088; AN 9: KU 327110–12; AS 2: USNM 229386; AS 5: KU 319965–67; C 13: KU 327113–5; D 4: KU 310093; MO 6: KU 334092–98; AN 1: CAS 133735, AN 3: CAS 133767; C 1: CAS 110984–88, CAS-SUR 28239–40, CAS-SUR 28242–46, CAS-SUR 28248–60, CAS-SUR 28277–313, CAS-SUR 28373; C 2: CAS-SUR 28210; C 6: CAS-SUR 26152, CAS-SUR 26157, CAS-SUR 26167–9, CAS-SUR 26182, CAS-SUR 26186–7, CAS-SUR 26297; C 7: CAS 137563, CAS-SUR 26163–64.

##### *Sphenomorphus variegatus* (Peters, 1867)

**Figure 61.**
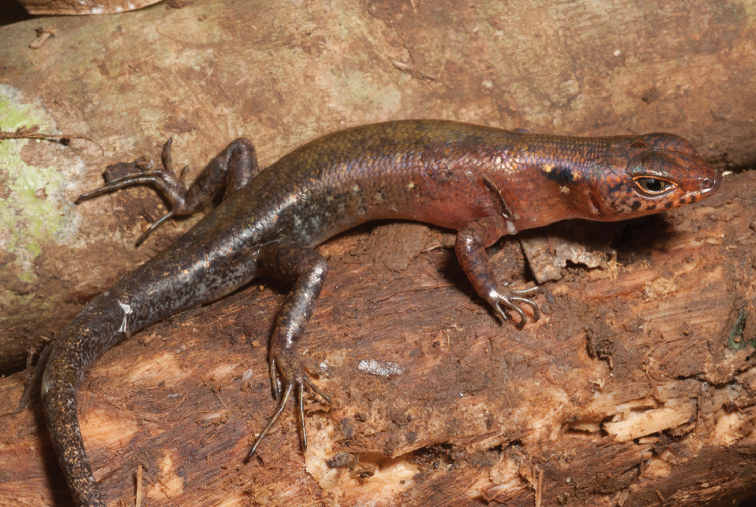
*Sphenomorphus variegatus* (KU 334312) from Mt. Hilong-hilong, Municipality of Remedios T. Romualdez, Agusan del Norte Province. Photo: RMB.

Unrelated to other Philippine scincid lizards, *Sphenomorphus variegatus* (Fig. 61) is a member of a clade consisting of non-Philippine species *Sphenomorphus maculatus*, *Sphenomorphus indicus*, *Sphenomorphus cyanolaemus*, *Sphenomorphus sabanus*, and *Sphenomorphus multisquamatus* (Linkem et al. 2011). The single Philippine representative of this clade, *Sphenomorphus variegatus* is a commonly encountered low elevation species wherever mature second growth or first growth forest is present on Mindanao (Taylor 1922a; Brown and Alcala 1980; Smith 1993a). This common widespread species’ conservation status has not been assessed by the IUCN (2016); we find that it does not qualify for any of the elevated threat categories, so we consider it as “Least Concern” (LC; IUCN 2010).

Sites and specimens: AN 11: KU 334309–15; AN 13: KU 334086; AS 2: USNM 229387; AS 5: KU 319941–47; C 13: KU 309899–907; D 5: KU 310115–32; MO 6: KU 334316–8; AN 1: CAS 133696, CAS 146461–2, CAS 133421, CAS 133596–97, CAS 133617, CAS 133654, CAS 133768–70, CAS 134230, CAS 154693–94; C 6: CAS-SUR 26171; C 7: CAS 137565, CAS-SUR 26149, CAS-SUR 26154, CAS-SUR 26292, CAS-SUR 26308, CAS-SUR 26628

##### *Tropidophorus misaminius* Stejneger, 1908

**Figure 62.**
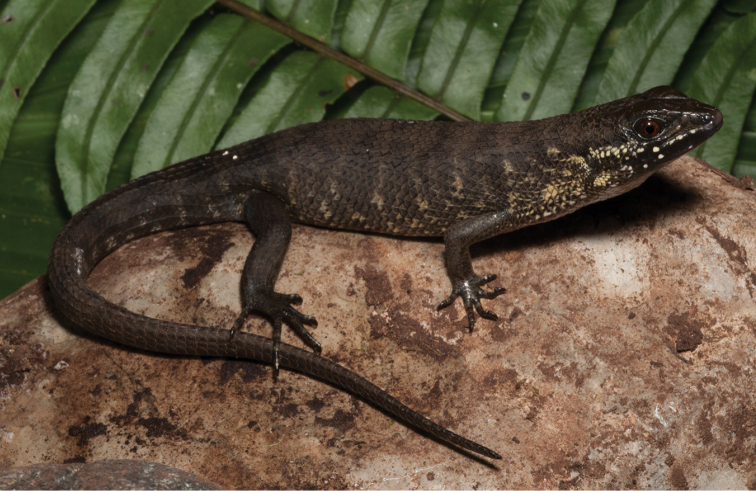
*Tropidophorus misaminius* male (KU 334249) from Mt. Hilong-hilong, Municipality of Remedios T. Romualdez, Agusan del Norte Province. Photo: RMB.

This Philippine endemic species (Fig. 62) is restricted to Basilan, Camiguin Sur, Dinagat, and Mindanao (type locality: Mt. Malindang, western Mindanao). During the day, we collected specimens on stream banks as well as in the forest where they were under logs or out in the open on the forest floor. At night, we found them sleeping among rocks and logs on stream banks. This species would often flee to the water in the presence of humans, demonstrating a remarkable adaptation for a semiaquatic lifestyle. Our observations of this species were restricted to primary and secondary growth forests, leading us to believe it is not tolerant of degraded habitat. However (Beukema 2006) reports this species as being able to tolerate some degree of habitat disturbance. This species is currently listed as “Least Concern” (LC; IUCN 2016).

Sites and specimens: AN 1: CAS 133759; AN 3: CAS 133480–84. AN 5: USNM 497032; AS 1: CAS 62047; C 1: CAS-SUR 28225–28, CAS-SUR 28352; C 6: CAS-SUR 26130, CAS-SUR 26135, CAS-SUR 26179, CAS-SUR 26239; C 7: CAS 139044–46; AN 10: KU 334253–55; AN 11: KU 334248–51; AN 12: KU 334252; AS 5: KU 320024, KU 320117–27; C 13: KU 315221; C 14: KU 315222–32; D 5: KU 310157–60; MO 2: KU 334256–64.

##### *Tropidophorus partelloi* Stejneger, 1910

Also a Philippine endemic, *Tropidophorus partelloi* is restricted to the islands of Mindanao and Siargao (Taylor 1922a; Brown and Alcala 1980; Ross and Lazell 1990) with an apparently wide yet patchy distribution that does not include areas impacted by anthropogenic disturbance (Smith 1993a). At one site (AN 12) we found this species in sympatry with *Tropidophorus misaminius*. We collected this species both on the banks of streams and in the forest well away from water. In the forest we collected specimens from under logs or from the forest floor. On the banks of streams this species was found both out in the open as well as asleep among rocks and logs. Similarly to *Tropidophorus misaminius*, this species will flee to the water when disturbed. This species is currently listed as “Least Concern” (LC; IUCN 2016).

Sites and specimens: AN 12: KU 334232–3; AS 5: KU 320022–3; AN 3: CAS 133404; AN 4: CAS 152195.

#### Family Varanidae

##### *Varanus cumingi* Martin, 1839

**Figure 63.**
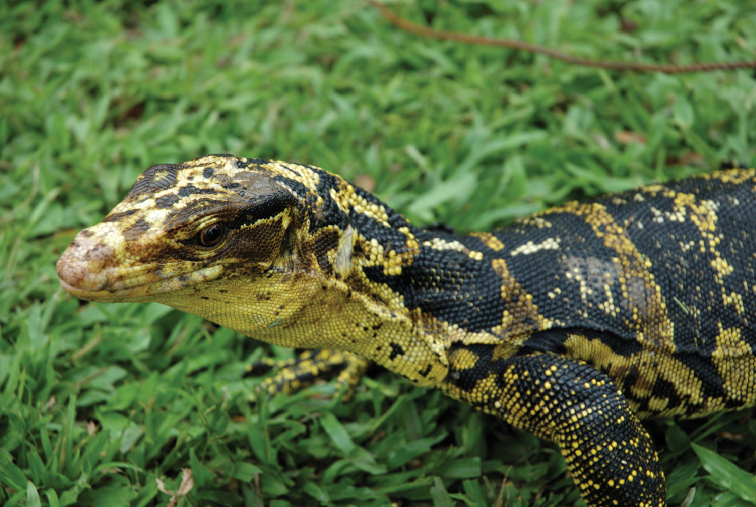
*Varanus cumingi* photographed at the Municipality of Mambajao, Camiguin Sur Island. Photo: CDS (specimen not collected)

This Mindanao faunal region endemic monitor lizard recently was distinguished, at the level of full species, from its closest known relative *Varanus samarensis*, an endemic species native to Bohol, Samar and Leyte islands, just north of Mindanao proper (Welton et al. 2013a, 2014a); these two species apparently diverged as long as 2 million years before present (1.5–2.5; Welton et al. 2014b). *Varanus cumingi* (Fig. 63) is an extremely common, widespread species and its conservation classification is “Least Concern” (LC; IUCN 2016). Our specimen from Mt. Lumot was salvaged from a villager who had killed the animal as a pest (it had raided his chicken coop on several recent occasions, in search of eggs and newly hatched chicks). We salvaged the decomposing specimen after several days; it has been prepared as an osteological specimen, making it the only known skeleton of this species in existence.

Sites and specimens: AN 2: KU 335559.

### Reptilia: Snakes

#### Family Colubridae

##### *Ahaetulla prasina preoccularis* (Taylor, 1912)

*Ahaetulla prasina* is a common, widely distributed snake encountered in a variety of habitats throughout the Philippines (Leviton 1963a, 1967). We frequently encounter this species at night, sleeping on branches of bushes and saplings in low elevation, selectively logged forest, primary forest, or even secondary growth or along edges of agricultural areas. We noted exceptionally high densities of this species at low elevation, in riparian habitats on Mt. Hilong-hilong. It is considered “Least Concern” (LC; IUCN 2016) for conservation purposes.

Sites and specimens: AN 10–KU 334411–21; AN 11: KU 334407–09; AN 13: KU 334410; D 5: KU 310162; MO 6: KU 334422–30; AN 3: CAS 133398.

##### *Boiga cynodon* (Boie, 1827)

Leviton (1970a) described *Boiga cynodon* as widely distributed in Southeast Asia and the Philippines, as part of the enormous range of this highly variable species. We collected one specimen on Dinagat Island and assume that it occurs in riparian habitats throughout the Caraga region and all of northeast Mindanao. Although it is classified as “Least Concern” (LC; IUCN 2016), we anticipate that eventual taxonomic studies may find this species to be a complex of evolutionary lineages, which will require individual evaluation in the future.

Sites and specimens: D 5: KU 310161.

##### *Calamaria gervaisi* Duméril, Bibron & Duméril, 1854

**Figure 64.**
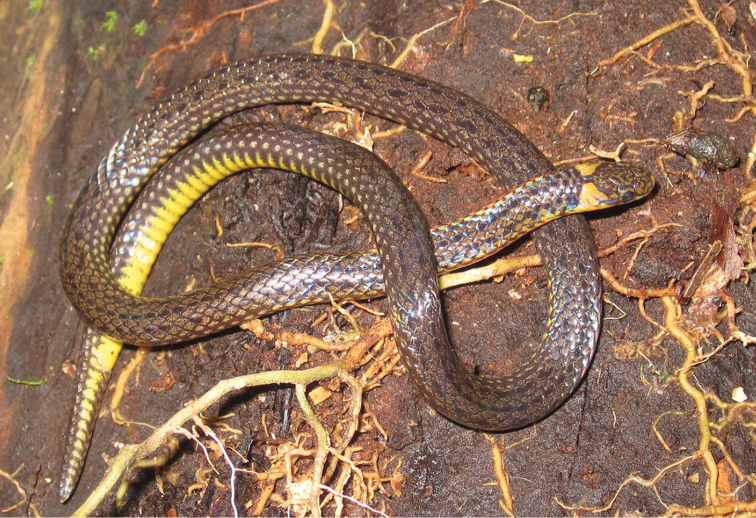
*Calamaria gervaisi* from Mt. Hilong-hilong, Municipality of Remedios, T. Romualdez, Agusan del Norte Province. Photo: KAC.

One of the most common fossorial Philippine snakes, *Calamaria gervaisi* (Fig. 64) is widely distributed in the Philippines (Inger and Marx 1965), and is recorded in most forest surveys of herpetological diversity (Brown et al. 1996, 2000a, 2012; McLeod et al. 2011; Siler et al. 2011, 2012). This species does not qualify for any elevated level of endangerment and is considered “Least Concern” (LC; IUCN 2016).

Sites and specimens: AN 13: KU 334486; AN 5: USNM 496807–8; MO 2: KU 334482, KU 334487; AN 3: CAS 133478; AN 4: CAS 133136–7, CAS 133253.

##### *Calamaria lumbricoidea* H. Boie in F. Boie, 1827

**Figure 65.**
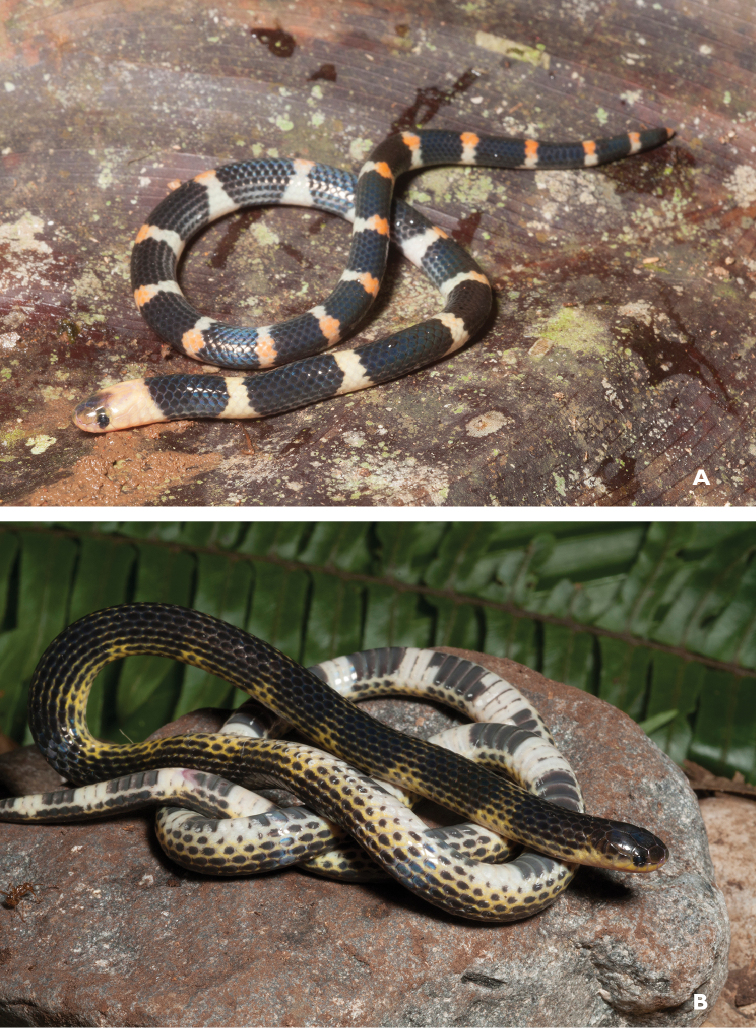
Ontogenetic color and pattern variation in juvenile (**A** KU 334478) versus adult (**B** KU 334476) specimens of *Calamaria lumbricoidea* from May-Impit, Mt. Hilong-hilong, Municipality of Remedios T. Romualdez, Agusan del Norte Province. Photos: RMB.

*Calamaria lumbricoidea* is frequently encountered throughout the Mindanao PAIC islands and we collected specimens from sea level to 1,200 m on several mountains of northeast Mindanao. It is widespread in Southeast Asia (type locality: Java). Its color pattern suggests adaptation for prey avoidance via a coral snake mimicry system, and its overall similarity to banded forms of *Hemibungarus* and *Calliophus* is striking (Fig. 65). This species is classified as “Least Concern” (LC; IUCN 2016).

Sites and specimens: AN 11: KU 334476; AN 12: KU 334477; AN 5: USNM 497033, KU 319955; C 13: KU 309919, KU 309946; C 14: KU 309920; MO 6: KU 334478–79; AN 3: CAS 133260; C 1: CAS-SUR 28275; C 7: CAS 137540; SS 2: CAS-SUR 24209.

##### *Chrysopelea paradisi* Boie, 1827

This widespread, non-endangered (“Least Concern;” IUCN 2016) species has been encountered on Dinagat Island several times, but only once in northeast Mindanao proper. This common “flying snake” has been reported to us on numerous occasions in the Caraga region by farmers and workers in coconut palm plantations who witness their directed aerial descent as a frequent locomotor mode (Dudley et al. 2007) during active foraging during the day. This species is infrequently encountered sleeping in vegetation 2–4 meters above the ground (in contrast to species of *Dendrelaphis* and *Ahaetulla*), suggesting its preferred retreat is higher in the forest canopy (or coconut palms) than biologists had suspected previously (Leviton 1964d). This species is classified as “Least Concern” (LC; IUCN 2016).

Sites and specimens: D 1: USNM 229322–23; D 2: KU 305494; D 5: KU 310165; AN 1: CAS 133711.

##### *Coelognathus erythrurus erythrurus* (Duméril, Bibron & Duméril, 1854)

Composed of four currently recognized subspecies, this Philippine rat snake has three distinctive phenotypes in the Philippines, corresponding to named taxa: *Coelognathus erythrurus manillensis* from Luzon (Brown et al. 1996, 2000, 2012, 2013; McLeod et al. 2011; Siler et al. 2011; Devan-song and Brown 2012), *Coelognathus erythrurus psephenoura* from the West Visayan, central islands of Negros, Cebu, Panay and possibly the Romblon Island Complex (Leviton 1979; Ferner et al. 2001; Gaulke 2011; Siler et al. 2012a), and *Coelognathus erythrurus erythrurus*, a form originally described from Java, Indonesia, but also reported on various islands of the Mindanao PAIC (Leviton 1979), including Mindanao, Camiguin Sur, Samar, and here: Dinagat (new island record). This complex, suspected to be composed of multiple evolutionary lineages deserving a specific rank, has not been evaluated by IUCN (2016) for conservation purposes.

Sites and specimens: AS 5: KU 319988; C 1: CAS-SUR 28272; C 14: KU 309924; D 4: KU 305168, KU 305648.

##### *Cyclocorus nuchalis taylori* Leviton, 1967

**Figure 66.**
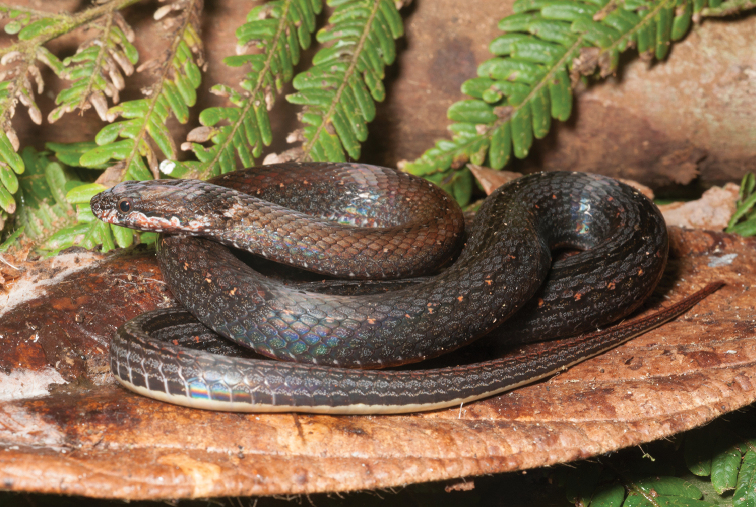
*Cyclocorus nuchalis taylori* (KU 334469) from May-Impit, Mt. Hilong-hilong, Municipality of Remedios T. Romualdez, Agusan del Norte Province. Photo: RMB.

Reported first by Taylor (1923), Leviton (1967) noted an apparent east–west hiatus in scalation across central Mindanao and recognized *Cyclocorus nuchalis nuchalis* (Zamboanga Peninsula’s southern most populations, and Basilan Island) for the western form and *Cyclocorus nuchalis taylori* (Fig. 66) for Mindanao’s eastern population. Since being reported by Smith (1993b) and Beukema (2011), we have also collected the species recently on Camiguin Sur, and Dinagat islands in addition to populations on Siargao Island (Ross and Lazell 1990), and farther north on Samar and Leyte islands. Considered “Least Concern” (LC; IUCN 2016), primarily due to its wide Philippine distribution, the taxonomic and conservation status of the variable populations will need to be evaluated with a molecular phylogenetic analysis.

Sites and specimens: AN 12: KU 334469; AN 3: CAS 133261; AN 7: CAS 15242; C 1: CAS-SUR 28274; C 7: CAS 137541; D 5: KU 310377.

##### *Dendrelaphis marenae* Vogel & Van Rooijen, 2008

An extremely common, widespread Philippine arboreal snake, *Dendrelaphis marenae* was collected by us in Agusan, Mindanao and previously has been reported throughout the Philippines (e.g., Leviton 1968; Gaulke 2011; Siler et al. 2011, 2012; Brown et al. 2012a, 2013; Devan-song and Brown 2012). It has been documented previously in the northeast Mindanao faunal region by Ross and Lazell (1990; Siargao Island) and was recently recognized as a Philippine and Sulawesi form by Vogel and van Rooijen (2008) who split the eastern form (*Dendrelaphis marenae*: Sulawesi and the Philippines) from the western nominate population (*Dendrelaphis pictus*: Sunda shelf and Asian mainland). This species has not been assessed for conservation status but its wide distribution would lead is to consider it “Least Concern” (LC; IUCN 2010).

Sites and specimens: AS 4: KU 314131; AS 6: KU 314130.

##### *Dendrelaphis philippinensis* (Günther, 1879)

Previously considered part of a widespread polytypic species complex (Leviton 1968), referred to *Dendrelaphis caudolineatus*, the name *Dendrelaphis philippinensis* recently was resurrected by van Rooijen and Vogel (2012) and applied to southern Philippine populations. The holotype specimen of *Dendrelaphis philippinensis* was collected from northern Mindanao (Günther 1879), indicating that this application is most likely correct. The biogeographically anomalous distribution of the populations currently referred to *Dendrelaphis philippinensis* (van Rooijen and Vogel 2012)—including Mindanao PAIC landmasses, but also Cebu Island (the West Visayan faunal region), the Bicol Peninsula and Polillo islands (the Luzon faunal region; Brown and Diesmos, 2009)—will require independent verification via a molecular test of these revised species boundaries. This species currently has not been assessed for conservation status, but its wide distribution, if correct, would lead as to consider it “Least Concern” (LC; IUCN 2010).

Sites and specimens: AN 11: KU 334483; C 6: CAS-SUR 26328; C 14: KU 310367; D 4: KU 310379.

##### *Gonyosoma oxycephalum* (Boie, 1827)

**Figure 67.**
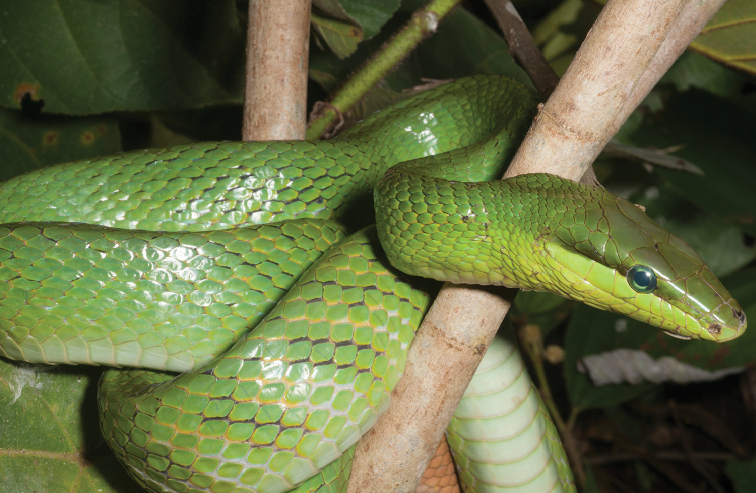
*Gonyosoma oxycephalum* has been collected on Dinagat Island and in Agusan del Sur; this specimen was photographed at the Municipality of Burauen, Leyte Island (specimen deposited in KU). Photo: RMB.

Reported at numerous localities throughout the Philippines, this widespread but relatively infrequently collected arboreal rat snake (Fig. 67) is known from the Luzon (Brown et al. 1996, 2013; Siler et al. 2011; Devan-song and Brown 2012), West Visayan (Ferner et al. 2000; Gaulke et al. 2011), Dinagat Island (Ross and Lazell 1990) Agusan, Mindanao (this study), Sulu (Gaulke 1994), Palawan (Boulenger 1894), and Romblon (Sy and Tan 2013) faunal regions. It is not considered threatened under the current understanding of its distribution and taxonomy (LC; IUCN 2016).

Sites and specimens: D 5: KU 310177; AS 3: CAS 15260.

##### *Lycodon capucinus* (Boie, 1827)

**Figure 68.**
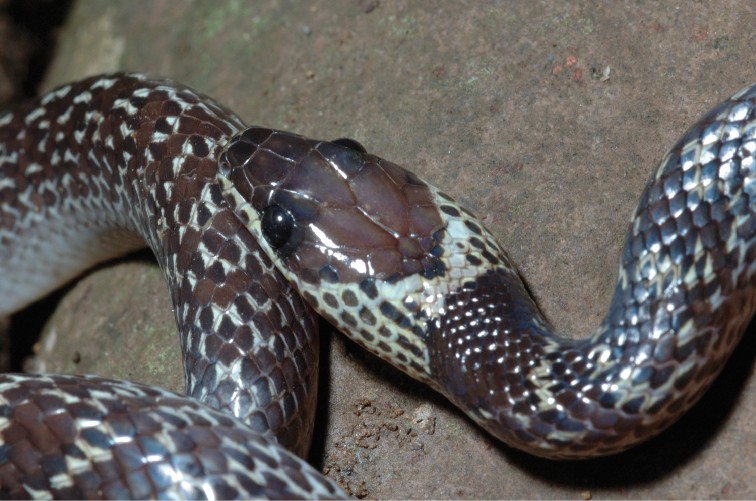
*Lycodon capucinus* (KU 305142) from Barangay Cabuan, Municipality of Guinsiliban, Camiguin Sur Island. Photo: CDS.

This common snake (Fig. 68) is encountered in the vicinity of human dwellings throughout the Philippines and Southeast Asia (Leviton 1963a, 1965b; Manthey and Grossman 1997; Inger and Voris 2001). Our previous phylogenetic analysis detected moderate genetic divergence among populations across its range, including the Philippines and Asian mainland (Siler et al. 2013). This species is “Least Concern” (LC; IUCN 2010).

Sites and specimens: AN 9: KU 327417; C 1: CAS-SUR 28271; C 10: KU 305142; D 4: KU 310166; SN 1: CAS 134506.

##### *Lycodon dumerilii* (Boulenger, 1893)

**Figure 69.**
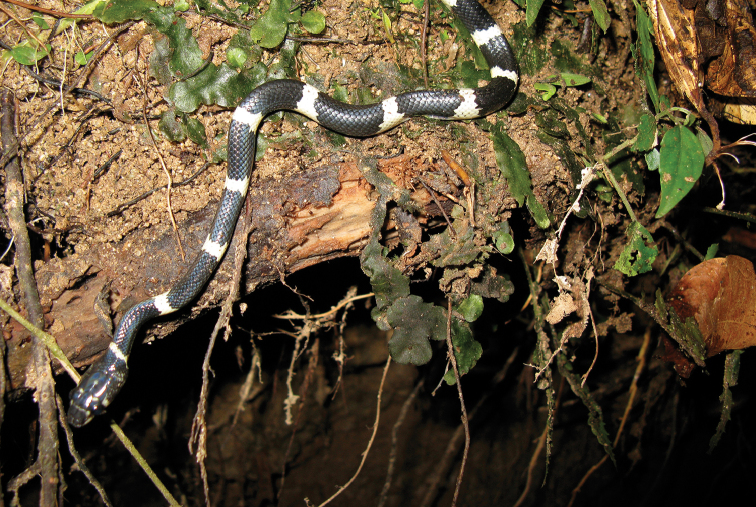
*Lycodon dumerilii* (KU 319989) from Mt. Magdiwata, Barangay Bagusan II, Municipality of San Francisco, Agusan del Sur Province. Photo: ACD.

This frequently encountered northeast Mindanao faunal region endemic has been reported from Mindanao and Samar (Taylor 1922e; Brown and Alcala 1970), and Leviton restricted the unspecified “Philippines” type locality of Boulenger (1893) to Surigao, northeast Mindanao (Leviton 1965b). Lanza (1999) described an apparently related species (*Lycodon ferroni*) from Samar, and the phylogenetic distinctiveness of the various, somewhat variable (RMB *personal observations*). Northeast Mindanao faunal region populations (Fig. 69) has not yet been examined in detail (Siler et al. 2013). This species is ranked “Least Concern” (LC; IUCN 2016).

Sites and specimens: AS 5: KU 319989; D 4: KU 306193; D 5: KU 310167–68.

##### *Oligodon maculatus* (Taylor, 1918)

**Figure 70.**
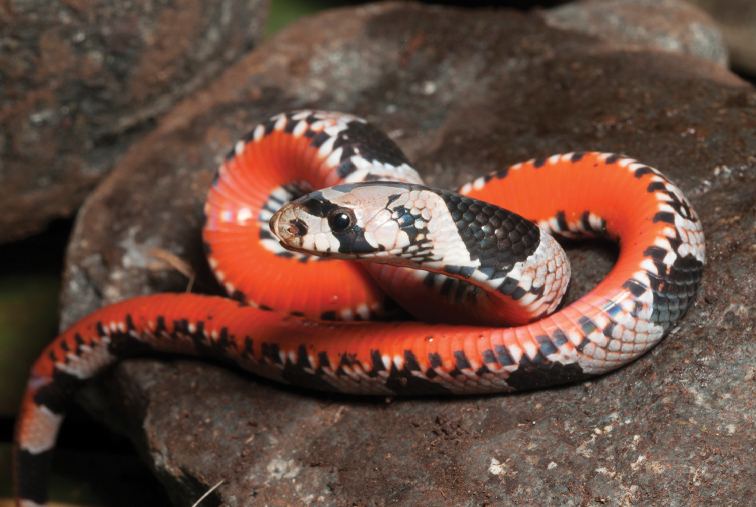
*Oligodon maculatus* juvenile (KU 334450) from Eye Falls, Mt. Hilong-hilong, Municipality of Remedios T. Romualdez, Agusan del Norte Province Photo: RMB.

Described by Taylor (1918a,b) from his Bunawan collections, *Oligodon maculatus* (Fig. 70) is recognized as a fairly common low- to mid-elevation snake from forested areas of the northeast Mindanao faunal region (Leviton 1963a,c; Smith 1993b). Considered “Least Concern” (LC; IUCN 2016) because of its widespread distribution, this species has not yet been recorded from any of the smaller islands, and its population status in western Mindanao is unclear. *Oligodon maculatus* is considered “Least Concern” (LC; IUCN 2016).

Sites and specimens: SS 3: CAS 15280; AN 10: KU 334451; AN 11: KU 334450.

##### *Stegonotus muelleri* Duméril, Bibron & Duméril, 1854

**Figure 71.**
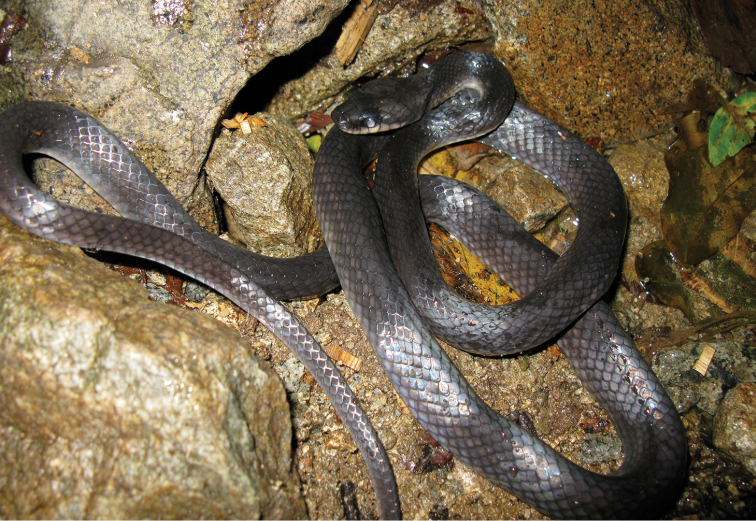
*Stegonotus muelleri* (KU 320004) from Mt. Magdiwata, Barangay Bayugan II, Municipality of San Francisco, Agusan del Sur Province. Photo: ACD.

Presumably because of the common species epithet, this species has been erroneously confused in taxonomic literature with *Lycodon muelleri* a species from Luzon, Polillo, and presumably Catanduanes and Marinduque islands (Siler et al. 2013). *Stegonotus muelleri* (Fig. 71), in fact is a large (1.5–2.0 m body length) rat snake that is unique among Mindanao PAIC herpetofauna in that it is a true Papuan biogeographical element, akin to the frog genus *Oreophryne* and extends no farther into the Philippines than the Mindanao faunal region (Inger 1954, 1967, 1999; Leviton 1963a; Brown and Alcala 1970). We have collected this common species throughout northeast Mindanao and Dinagat islands despite the fact that a decade ago, it qualified as “Near Threatened” (NT; IUCN 2010, 2016); the status of this common widespread species needs to be reconsidered now that there are new distributional data are available. We consider the species to be “Least Concern” (LC; IUCN 2010) given all of our data presented here and those not yet published from Leyte and Samar islands.

Sites and specimens: AN 11: KU 334773–74; AS 5: KU 320001–04; D 5: KU 310178; MO 6: KU 334775.

#### Family Natricidae

##### *Rhabdophis auriculata auriculata* (Günther, 1858)

**Figure 72.**
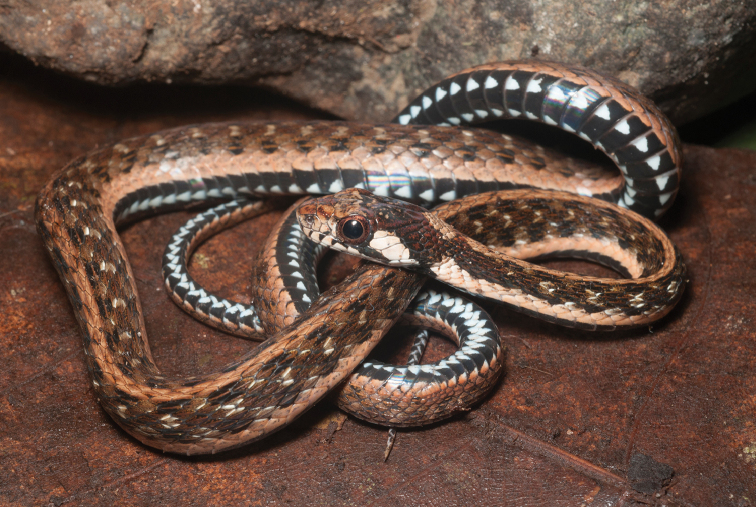
*Rhabdophis auriculata auriculata* (KU 334441) from Eye Falls, Mt. Hilong-hilong, Municipality of Remedios T. Romualdez, Agusan del Norte Province. Photo: RMB.

A widespread and exceptionally abundant species *Rhabdophis auriculata* (Fig. 72) is another species with taxonomic differentiation across central Mindanao (Günther 1858; Taylor 1922e; Leviton 1963a). Leviton (1970b) described the subspecies *Rhabdophis auriculata myersi* from western Mindanao and recognized the nominate form from central and eastern Mindanao, Bohol, Samar, Leyte islands. All published reptile faunal studies from Mindanao have noted this species (e.g. Smith 1993b; Beukema 2011), and we have collected it on the forest floor and riparian habitats in disturbed areas at numerous sites on Mindanao and Dinagat (Ross and Lazell 1990). This species is not considered threatened (LC; IUCN 2016).

Sites and specimens: AN 11: KU 334437–42; AN 12: KU 334443; AN 3: CAS 133456–57, CAS 133479, CAS 133499, CAS 133598, CAS 186141; AN 4: CAS 133360–61; AN 5: USNM 305863–67, USNM 497034–35, KU 319990–4; D 5: KU 310172–73, KU 310376; MO 2: KU 334444–8; MO 5: KU 319995; MO 6: KU 334449.

##### *Rhabdophis lineatus* (Peters, 1861)

**Figure 73.**
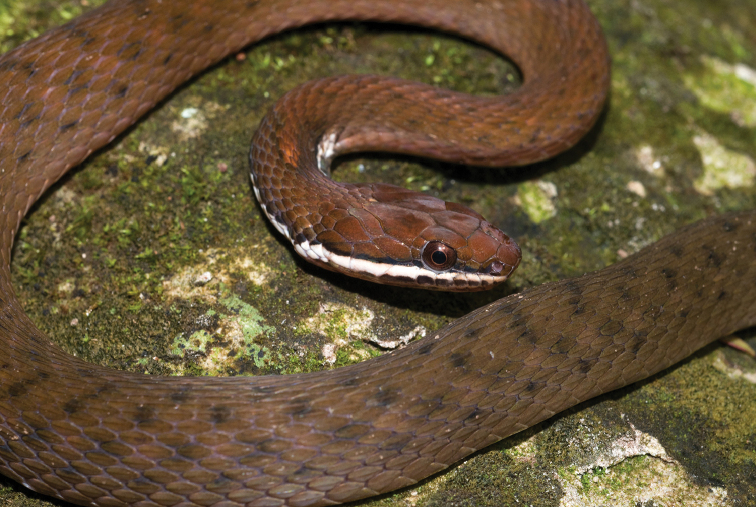
*Rhabdophis lineatus* (KU 334463) from Mt. Lumot, Municipality of Gingoog City, Misamis Oriental Province. Photo: RMB.

Another extremely common and widespread Mindanao faunal region species (Boulenger 1893; Taylor 1922c,e; Leviton 1963a), *Rhabdophis lineatus* (Fig. 73) commonly is encountered in the vicinity of fresh water at lower elevations on all the large islands of the Mindanao PAIC (Brown and Diesmos 2009). This species is not currently considered threatened (IUCN 2016), even by heavy disturbance to coastal freshwater environments, where it apparently persists without noticeable declines in abundance (RMB *personal observations*).

Sites and specimens: AN 11: KU 334452–59; AN 3: CAS 133365, CAS 133498, CAS 133502, CAS 133599, CAS 186140, CAS 186142; AN 4: CAS 133231, CAS 133286, CAS 133359; AN 5: USNM 497036; AS 5: KU 319956–9; D 5: KU 310174–75; MO 2: KU 334461–63; MO 3: KU 334460; MO 5: KU 319960; MO 6: KU 334464–67.

##### *Tropidonophis dendrophiops* (Günther, 1883)

**Figure 74.**
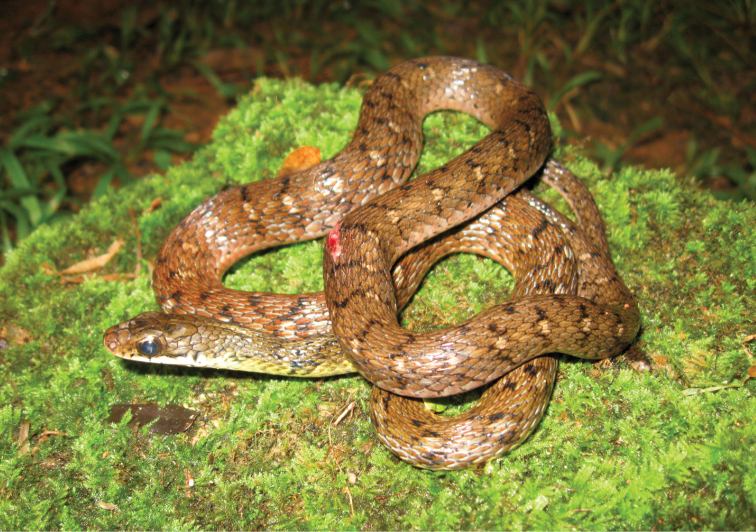
*Tropidonophis dendrophiops* (KU 319996) from Mt. Magdiwata, Barangay Bayugan II, Municipality of San Francisco, Agusan del Sur Province. Photo: ACD.

Described from Zamboanga, western Mindanao (Günther 1883; Boulenger 1893), this species (Fig. 74) is infrequently collected on Mindanao (Malnate and Underwood 1988; Smith 1993b) but can be encountered on Luzon fairly predictably in riparian habitats with clean water and some extent of vegetation cover (Brown et al. 1996, 2000, 2012, 2013a; Siler et al. 2011a; Devan-song and Brown 2012; McLeod et al. 2012). Given its wide distribution, this species is considered “Least Concern” (LC; IUCN 2016). A phylogeographic study, combined with appraisal of geographic variation from Luzon to Mindanao would be a desirable undertaking with this taxon, which we consider sensitive to environmental disturbance and which we note is conspicuously absent from low elevation, coastal, and highly disturbed fresh waterways (RMB *personal observation*).

Sites and specimens: AS 5: KU 319996; C 13: KU 309921, KU 310368; MO 6: KU 334488; C 7: CAS 137559, CAS 185748.

#### Family Elapidae

##### *Calliophis philippina* (Günther, 1864)

**Figure 75.**
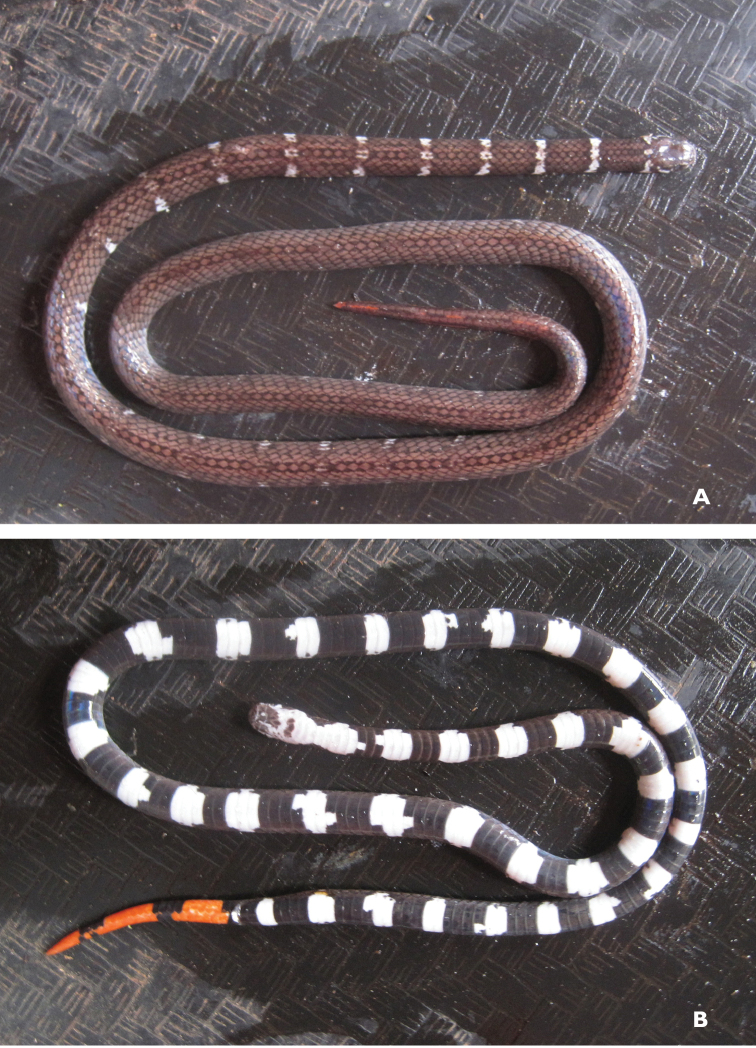
*Calliophis philippina* (KU 320005; **A** dorsal **B** ventral) from Mt. Lumot, Municipality of Gingoog City, Misamis Oriental Province. Photo: KAC.

This species (Fig. 75) is widespread in the Mindanao PAIC islands of Mindanao, Samar and Leyte (Leviton 1963b; Leviton et al. 2014) and is known from Agusan del Norte and Misamis Occidental provinces in the northeast, as well as Zamboanga Province in the west, and Davao and Lanao del Sur provinces in the south. Previously considered a subspecies of the widespread polytypic *Calliophis intestinalis* (which is considered “Least Concern” by IUCN [2016]) the revised taxonomy, considering Mindanao PAIC populations as a separate species has not yet been assessed for conservation purposes. Nevertheless, we consider this to be a reasonably common species in areas with any kind of forest cover and we have encountered specimens in many of our recent surveys in Agusan Del Norte and Agusan Del Sur provinces (Mindanao Island), and Camiguin Sur Island, as well as on recent surveys in extreme western Mindanao. Based on its wide distribution and lack of any specific threats, we consider this species to be “Least Concern” (IUCN 2010).

Sites and specimens: AN 9: KU 327217; AS 5: KU 320005; C 1: CAS-SUR 28273; C 13: KU 327218; C 14: KU 310369; C 6: CAS-SUR 26189; MO: KU 334481.

##### *Calliophis* sp.

We collected single specimen of a new species of elapid snake, tentatively assigned to the genus *Calliophis*, at the Barangay Santiago, Sitio Cambinlia (Sudlon), Municipality of Loreto, Dinagat Island. The specimen is so distinct as to initially defy identification to genus (Leviton et al. 2014). Possibly more closely related to the red-headed blue coral snake (*Calliophis bivirgata*) than to the sympatric and common striped coral snake *Calliophis philippina* (Eric Smith, *personal communication*), the new species will formally be named to science in another publication. A previous molecular phylogenetic analysis of Philippine “coral snakes” of the genus *Hemibungarus* determined those Philippine elapids were unrelated to true coral snakes and, rather, more closely related to cobras (Castoe et al. 2007). A phylogenetic analysis of DNA sequence data will likewise be necessary to determine the phylogenetic affinities of this unusual new species (E. Smith and RMB *unpublished data*). Without any information on the distribution of this new species, we hold conservation status assessment in abeyance until more data are available.

Sites and specimens: D 5: KU 310164.

##### *Naja samarensis* Peters, 1861

This venomous cobra species is known throughout Mindanao, Bohol, Samar, Leyte, Dinagat, and Camiguin Sur islands (Taylor 1922e; Leviton 1963a, 1964b; Wüster and Thorpe 1990; Smith 1993b; Leviton et al. 2014). Our specimen from Barangay Pandan, Municipality of Mambajao (Camiguin Sur Island) was active during the day on the edge forest and coconut palm plantations. Like all recognizably venomous snakes, Samar cobras are heavily persecuted by humans whenever the two species come in contact; however, *Naja samarensis* is known from a number of protected areas, and clearly can tolerate anthropogenic modification of its habitat; it is considered “Least Concern” (LC; IUCN 2016) despite the fact that all cobras are included in Appendix II of CITES (IUCN 2016) due to their commercial harvest for skin (leather) and traditional medicine trades.

Sites and specimens: AN 7: CAS 15340; C 14: KU 309923; C 7: CAS-SUR 26322; D 1: USNM 229324.

##### Family Hydrophiidae

Leviton et al. (2014) list multiple sea snakes form the coastal areas of Mindanao, including *Hydrophis atricepts* and *Laticauda laticaudata*. We were able to locate credible, identified specimens of only a single species from northeast Mindanao, although we do not discount sight records of earlier workers (Taylor 1922d; Leviton 1963; Brown and Alcala 1970).

##### *Hydrophis platyurus* (Linnaeus, 1766)

This extremely widely distributed sea snake is most likely found throughout the Philippines (Leviton et al. 2014), and outside of the country as far as Hawaii and the western coastal United States. The two specimens from Surigao Del Norte are hemipene preparations, taken by J. C. Thompson (a U. S. Marines office, stationed in the Philippines in the early 1900s) from voucher specimens deposited then in the University of Santo Tomas (Manila) collection. No information is available regarding the condition of these voucher specimens in the UST collection, and we have not been granted access to this historically important Manila-based collection (Taylor 1920a,b, 1922a,e; Brown and Alcala 1978). This species has been assessed by the IUCN as “Least Concern” with populations stable (IUCN 2016). The status of sea snakes in the waters surrounding Mindanao has been very poorly studied and almost nothing is known of species diversity in the southwest Philippines (Taylor 1922e; Uetz and Hošek 2015).

Sites and specimens: SN 1: CAS 15364–65.

#### Family Lamprophiidae

##### *Oxyrhabdium modestum* (Duméril, 1853)

**Figure 76.**
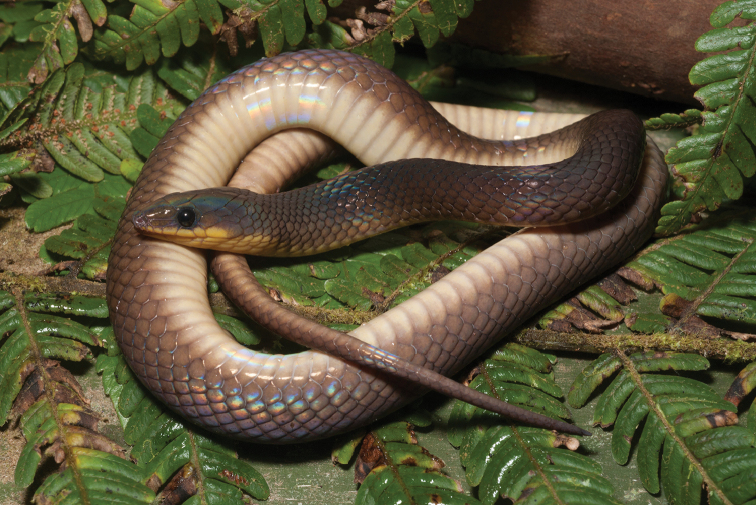
*Oxyrhabdium modestum* (KU 334388) from May-Impit, Mt. Hilong-hilong, Municipality of Remedios T. Romualdez, Agusan del Norte Province. Photo: RMB.

Like the closely-related Luzon PAIC species *Oxyrhabdium leporinum*, individuals of this common Mindanao PAIC faunal region endemic (Fig. 76) are most frequently encountered actively foraging on the ground in riparian habitats (Leviton 1964c; Brown et al. 2000a, 2012, 2013b; McLeod et al. 2011; Siler et al. 2011a; Devan-Song and Brown 2012). Juveniles of both species are encountered at night, perched asleep in herbaceous layer vegetation and small shrubs. Many of our specimens were found coiled on fronds or in axils of ferns along stream banks in selectively logged forests; others were found on the first few branches of small saplings. Owing to its ubiquitous distribution throughout the Mindanao PAIC and absence of any specific threats, *Oxyrhabdium modestum* is considered “Last Concern” (LC; IUCN 2016).

Sites and specimens: AN 10: KU 334390–91; AN 12: KU 334386–88; AN 14: KU 334389; AN 9: KU 327247; C 13: KU 309912–15, KU 310361–2, KU 327248–50; C 14: KU 309916–18, KU 310363–66; MO 1: KU 327251–52; MO 6: KU 334391–402; AN 3: CAS 133363, CAS 133399–401; AN 4: CAS 133135, CAS 133208, CAS 133239; C 1: CAS-SUR 28213; C 2: CAS-SUR 28276; C 6: CAS 139047, CAS-SUR 26190, CAS-SUR 26325–27, CAS-SUR 26329; C 7: CAS 137555–58, CAS 185747.

##### *Psammodynastes pulverulentus* (Boie, 1827)

This non-endemic, very widespread species has not been assessed by IUCN (2010, 2016) but clearly is not threatened with extinction due to its widespread distribution across many Southeast Asian countries and landmasses, along with its obvious tolerance of disturbance. We frequently find adults on the ground foraging, and juveniles in the branches (sleeping at night) of very small saplings. This species is not considered endangered at any elevated threat level (LC; IUCN 2016).

Sites and specimens: AN 12: KU 334384; AN 5: USNM 305862, USNM 497037; AS 5: KU 319997–99; AS 6: KU 314132; C 13: KU 309922; D 5: KU 310169–71; MO 2: KU 334385; AN 3: CAS 133477, CAS 133540; AN 4: CAS 133162, CAS 153870; C 1: CAS-SUR 28212; C 6: CAS 137560–61; CAS-SUR 26324; C 7: CAS 137562, CAS-SUR 26323.

#### Family Pareidae

##### *Aplopeltura boa* (Boie, 1828)

**Figure 77.**
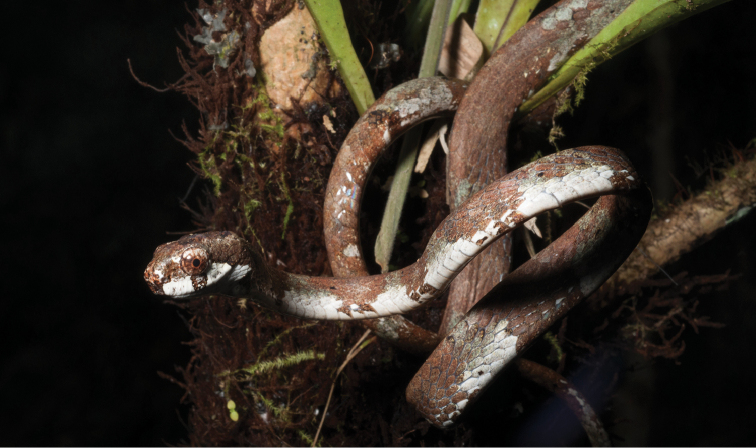
*Aplopeltura boa* (KU 334473) from Eye Falls, Mt. Hilong-hilong, Municipality of Remedios T. Romualdez, Agusan del Norte Province. Photo: RMB.

This widely distributed slug-eating snake (Fig. 77) is most frequently encountered in the vicinity of rivers and streams where it feeds exclusively on arboreal slugs and snails that are abundant following rain. It is considered “Least Concern” by IUCN (2016) due chiefly to its wide geographical distribution across many Southeast Asian landmasses.

Sites and specimens: AN 11: KU 334470–73; AN 12: KU 334474–75; AS 4: KU 314129; AS 5: KU 319950–54; D 5: KU 310163; AS 3: CAS 15351.

#### Family Pythonidae

##### *Malayopython reticulatus* (Schneider, 1801)

Caraga region reticulated pythons are quite common in various habitats, including agricultural plantations, and even residential areas—localities where humans have subsidized their prey base by inadvertently increasing rodent populations. We received numerous reports of pythons from residents around disturbed forests patches in the foothills of Mt. Hilong-hilong, Mt. Balatukan, Mt. Magdiwata, and Mt. Lumot. Hunted for meat (Gaulke 1998), *Malayopython reticulatus* is also exploited in other parts of its range for leather. The conservation status of natural populations of Philippine pythons is unclear; this species has not been assessed by the IUCN (2016), but it most likely will not require additional measures of protection unless it becomes commercially exploited in the area. We collected one specimen in a coconut palm plantation at night, at the base (170 m) of Mt. Hilong-hilong.

Sites and specimens: AN 9: KU 334997.

#### Family Typhlopidae

##### *Ramphotyphlops braminus* (Daudin, 1803)

This common, parthenogenetic, and presumably introduced species were collected from beneath all kinds of forest floor debris (leaf litter, logs, rocks, etc.) and on the edge of forests, in agricultural and residential areas. Frequent around human dwellings, this common “flower pot snake” is “Least Concern” (LC; IUCN 2016).

Sites and specimens: C 13: KU 327418–21; C 1: CAS-SUR 28197–98, CAS-SUR 28206; C 6: CAS-SUR 26230.

##### Ramphotyphlops cf. cumingii (Gray, 1845)

Owing to the near complete lack of information on the distribution of *Ramphotyphlops cumingii* (Gray 1845; Taylor 1922e; McDowell 1974; McDiarmid et al. 1999), plus wholesale taxonomic confusion surrounding species boundaries in these secretive taxa (McDiarmid et al. 1999; Wynn et al. 2015), *Ramphotyphlops cumingi* is considered “Data Deficient,” as are most species of Philippine typhlopid snakes. Imperfectly matching published accounts of the diagnostic scalation in *Ramphotyphlops cumingii*, our specimen from low elevation foothills of Mt. Hilong-hilong (coconut palm plantations) may represent yet another undescribed form.

Sites and specimens: AN 10: KU 334468.

##### *Malayotyphlops* sp.

Collected in 1971 above 700 m on the west side of Mt. Hilong-hilong on approach from the Municipality of Cabadbaran, CAS 133604 is the only known specimen of an undescribed species. Despite our return to the general area in 2010 and 2012, no additional specimens have been secured. In previous assessments, this specimen has been identified as “*Typhlops luzonensis*” Taylor 1919, so comparisons to that species are warranted. In a recent review of *Malayotyphlops luzonensis*, Wynn et al. (2015) re-described *Malayotyphlops luzonensis* on the basis of the holotype (CM 2653) and other specimens from the type locality (Mt. Makiling, Laguna Province, Luzon Island) and determined that other non-Luzon populations previously referred to this species (from Negros and this specimen Mindanao) were misidentifications of other unrecognized taxa.

Sites and specimens: AN 3: CAS 133604.

#### Family Viperidae

##### Trimeresurus cf. flavomaculatus (Gray, 1842)

**Figure 78.**
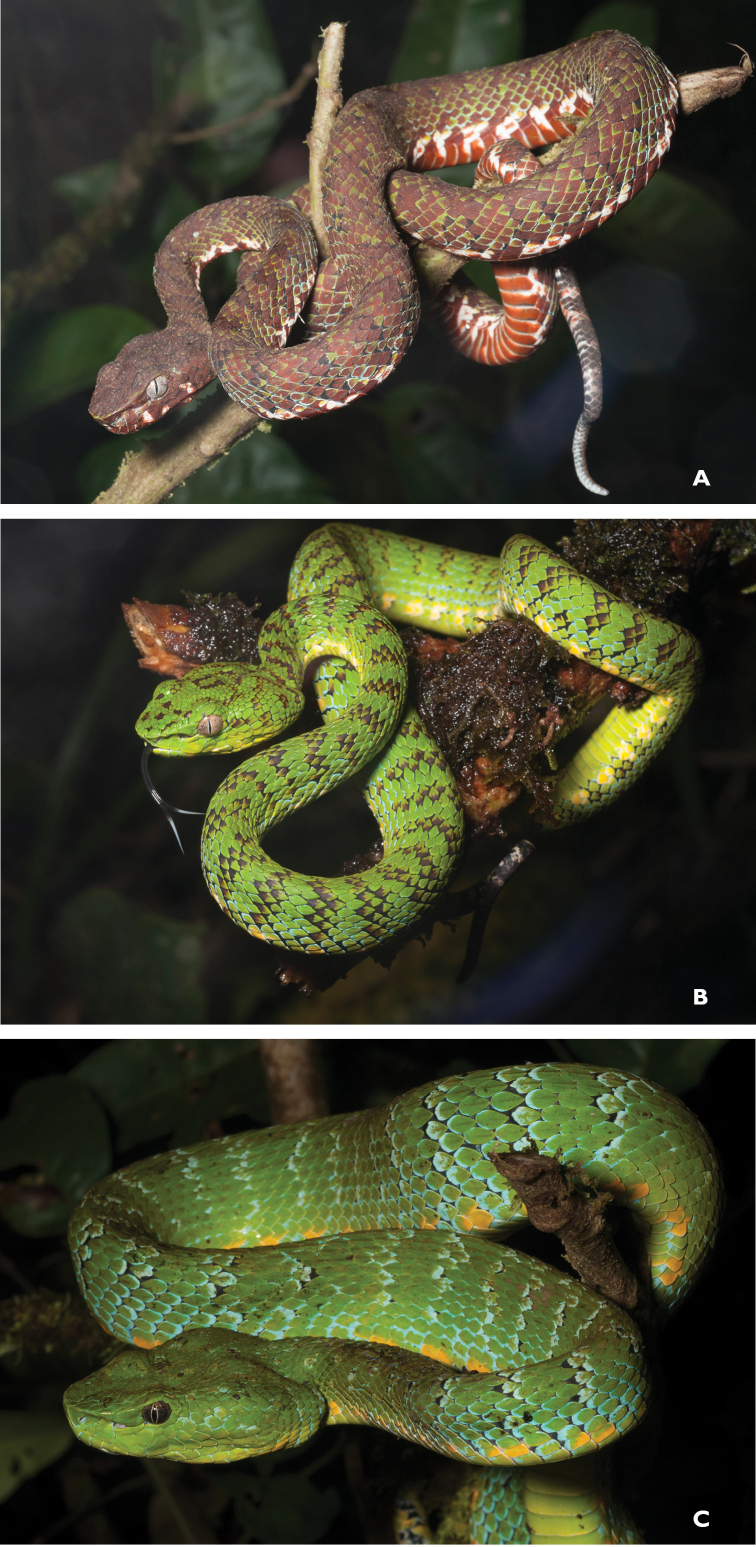
Trimeresurus cf. flavomaculatus red phase male (**A** KU 334433), green phase male (**B** KU 334466), green phase female (**C** KU 334432), all from Mt. Hilong-hilong, Municipality of Remedios T. Romualdez, Agusan del Norte Province. Photos: RMB.

*Trimeresurus flavomaculatus* (Fig. 78) is an exceedingly common pit viper, encountered in a variety of habitats on Luzon, Catanduanes, and Polillo (Taylor 1919; McLeod et al. 2011; Siler et al. 2011a; Brown et al. 2012a, 2013b; Devan-song and Brown 2012). On Mindanao PAIC islands, Trimeresurus cf. flavomaculatus is widespread and but somewhat common (Leviton 1964a; Leviton et al. 2014), prone to be encountered in mature forest, and with a distinctive color pattern. It is entirely possible that these observations may be simple inter-populational differences not indicative of species-level diagnostic characteristics. This certainly has been the case for *Trimeresurus flavomaculatus halieus*, a subspecies (*sensu* Leviton 1964a) originally described from Polillo Island (Taylor 1919) but which has been shown to be a mere color pattern variant, frequently found on Luzon (Brown et al. 2000, 2012, 2013a; Devan-song and Brown 2012; Leviton et al. 2014). On the other hand, previously recognized subspecies *Trimeresurus flavomaculatus mcgregori* (now recognized as *Trimeresurus mcgregori*, a Batanes-Babuyan archipelago endemic; Leviton et al. 2014), and *Trimeresurus flavomaculatus schultzei* (a Palawan endemic) have been elevated to the level of full species (Brown et al. 2000; Gumprecht et al. 2004; Oliveros et al. 2011; Leviton et al. 2014), leading us to consider a molecular test of species potential species boundaries in Luzon PAIC versus Mindanao PAIC *Trimeresurus flavomaculatus* populations highly desirable. Given its current “widespread” geographic distribution, *Trimeresurus flavomaculatus* currently is considered “Least Concern” (LC; IUCN 2016). This arrangement could change if Mindanao, west Visayan [Gaulke 2011], and/or Luzon populations are shown to be separate evolutionary lineages. Fischer (1885) described. *Trimeresurus schadenbergi* from Mindanao (currently a synonym of *Trimeresurus flavomaculatus*). This would seem to be the available name for the Mindanao faunal region lineage, should it be deemed worthy of specific rank. According to David and Ineich (1999), Leviton’s (1964a) restriction of the type locality of *Trimeresurus flavomaculatus* to Luzon may not be valid, thus necessitating that a full reconsideration of phenotypic variation in name-bearing types (or literature descriptions, given that some type specimens were destroyed in WWII; Brown and Alcala 1978; Uetz and Hosek 2015) be undertaken in coordination with any effort to distinguish these putative taxa using molecular data.

Sites and specimens: AN 11: KU 334432–33; AN 12: KU 334434–35; AS 5: KU 320006–07; D 5: KU 310378; MO 2: KU 334436.

##### *Tropidolaemus subannulatus* (Gray, 1842)

**Figure 79.**
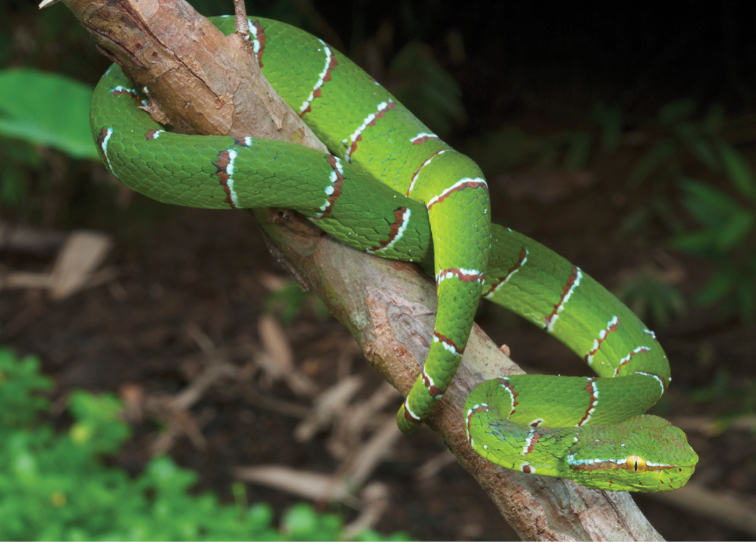
*Tropidolaemus subannulatus* (KU 306592) from Barangay Esperanza, Municipality of Loreto, Dinagat Island. Photo: CDS.

This common and widespread Luzon and West Visayan pit viper (Fig. 79) is frequently encountered in coastal to mid-montane elevation forested areas (Taylor 1919, 1922e; Leviton 1964a; Brown et al. 2012a, 2013a; Siler et al. 2011a; Devan-song and Brown 2012). Vogel et al. (2007) revised the *Tropidolaemus wagleri* complex and, in restricting *Tropidolaemus wagleri* to non-Philippine landmasses, resurrected Gray’s (1842) *Tropidolaemus subannulatus* for Philippine populations on numerous islands, including northeast Mindanao populations. The authors noted the “highly problematic” nature of these populations, with respect to their diagnosis from conspecific *Tropidolaemus philippensis* (below) and we concur: although some specimens clearly meet the definition of Gray’s *Tropidolaemus subannulatus*, others from the same or nearby populations are definitively *Tropidolaemus philippensis*. The status of these two possibly distinct and sympatric lineages needs to be addressed with molecular data and a full appraisal of variation in the name-bearing type specimens (Vogel et al. 2007). This species is considered “Least Concern” (LC; IUCN 2016).

Sites and specimens: AN 10: KU 334489; AN 5: USNM 305856; MO 6: KU 334490.

##### *Tropidolaemus philippensis* (Gray, 1842)

**Figure 80.**
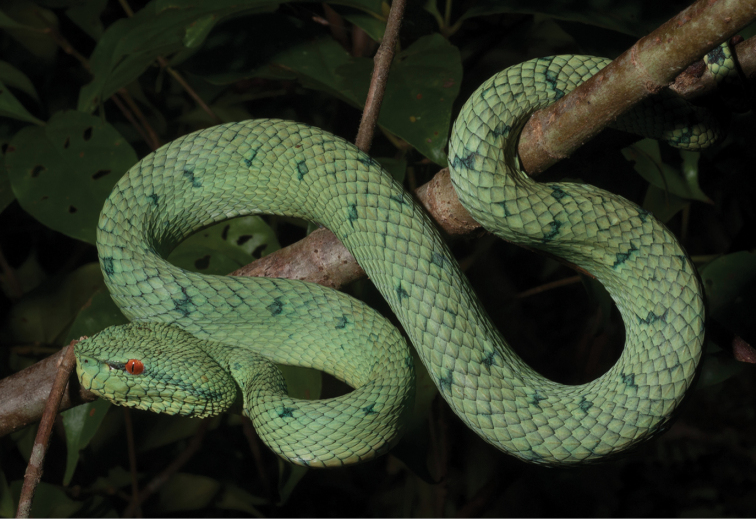
*Tropidolaemus philippensis* (KU 334490) from Mt. Lumot, Municipality of Gingoog City, Misamis Oriental Province. Photo: RMB.

As discussed above, Vogel et al. (2007) recently resurrected Gray’s (1842) *Tropidolaemus philippensis* (Fig. 80); both taxa are exemplified by holotypes bearing locality data no more specific than “The Philippines.” Although Taylor (1919, 1922e) recognized them both, Leviton (1964a) placed both in the synonymy of *Tropidolaemus wagleri* (Vogel et al. 2007; Leviton et al. 2014). That action, no longer tenable, has been revised to recognize all three species (and others) with the unresolved problem of a lack of clarification between *Tropidolaemus subannulatus* and *Tropidolaemus philippensis* (Vogel et al. 2007). With reference to clearly *Tropidolaemus philippensis* phenotypes from Samar and Leyte (e.g., KU 338863, 311290, 326317, from Leyte Island), we do not hesitate to identify a subset of our available specimens to *Tropidolaemus philippensis*. This species’ conservation status has not been assessed.

Sites and specimens: D 2: KU 306592; D 5: KU 310176.

### Reptilia: Turtles

#### Family Bataguridae

##### *Cuora amboinensis* (Riche in Daudin, 1802)

**Figure 81.**
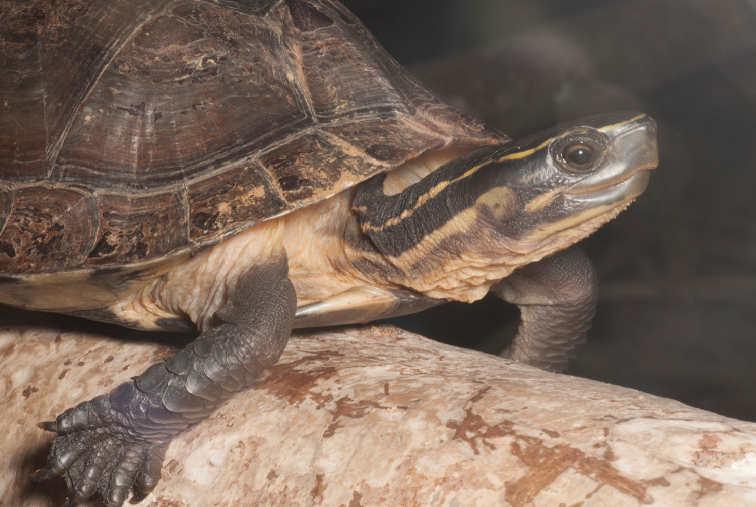
*Cuora amboinensis* is common at low elevations in riparian habitats throughout northeast Mindanao. Photo: RMB (from the Municipality of Balangiga, Samar Island; specimen deposited at KU).

This common geomydid freshwater turtle species (Fig. 81) has been documented by Diesmos et al. (2008) throughout the country. It is considered widely distributed and composed of two poorly differentiated subspecies, which may or may not be valid (the nominate form, *Cuora amboinensis amboinensis* is reported to occur throughout the eastern Philippines, including northeast Mindanao). Our records all originate from Dinagat Island where it is common on forest edges and agricultural areas and streams in secondary, disturbed forest. Although the taxonomy of this group is unresolved and despite its wide distribution throughout the Philippines, and adjacent landmasses of the western Australasian archipelago and adjacent mainland, this species is considered “Vulnerable” to extinction (VU; IUCN 2016) in recognition of the fact that freshwater turtles are so heavily exploited throughout Asia (Lehr and Holloway 2003; Schoppe 2008).

Sites and specimens: D 2: KU 305165, KU 305167, KU 305557–58; D 5: KU 310179.

### Reptilia: Crocodiles

#### Family Crocodylidae

##### *Crocodylus porosus* Schneider, 1801

This species, a saltwater and estuarine crocodile, is found distributed from Southeast Asia up to northern Australia (Iskandar 2000; Lewis et al. 2013). In the Philippines, Indo-pacific crocodiles are found throughout the archipelago and in north-central Mindanao; their presence previously has been documented inland at Agusan Marsh. The species inhabits inland lakes, swamps and marshes, as well as coastal brackish waters and tidal sections of rivers; terrestrial nest sites and basking areas are frequently observed along many Mindanao freshwater bodies of water. This species has an IUCN status of “Least Concern” (LC; IUCN 2016).

Sites and specimens: D 1: USNM 228414–16; SS 1: USNM 228413.

## Discussion

Our collective knowledge of the herpetological diversity of Mindanao Island (and, in general, the southern Philippines) relies on important mid- to late-19^th^ Century historical European museum collections and a few recent surveys, mostly concentrated at one region: northeast Mindanao (e.g., collections of E. H. Taylor, W. C. Brown and ACA; Plaza and Sanguila 2015), with scattered, limited-scope reports from the western (Nuñeza et al. 2010), central (Beukema 2011), and southeastern (Smith 1993a,b; Delima et al. 2006, 2007) corners of the island. The historical survey works of Taylor (1919, 1920a,b 1921, 1922a,b, 1923, 1960, 1965) based on his extensive survey work (1912–1915) from the north-central portion of the island (Taylor 1975) resulted in records of approximately 100 species, which provided the first picture of Mindanao’s herpetological diversity (Dickerson 1928; Taylor 1928, 1975). After this seminal work, the only subsequent summaries of Mindanao fauna were those of revisionary taxonomists (Inger 1954; Leviton 1963a–c, 1964a–c, 1965a,b, 1967, 1968, 1970a,b, 1979, 1983) and the biogeographical reviews of Brown and Alcala (1970, 1975, 1980, 1994), which relied primarily on Taylor’s specimens and augmented his records with all that was known of the island at the time (ancillary collections). Since Taylor’s work, the majority of our understanding of the herpetofaunal diversity of Mindanao (at least in terms of published literature and data in the public domain) has been augmented by excursions concentrated on specific sites. These include the records of 14–41 reptile species (Amoroso 2000; Beukema 2010) and 25 amphibian species records from central Mindanao (Delima et al. 2006), 34 species from southeastern portions of the island (Delima et al. 2007; Ates et al. 2009), 27 species reported from Mt. Hilong-hilong (Plaza and Sanguila 2015), 26–59 species recorded in the northwestern reaches of Mindanao (Tabaranza et al. 2001; Nuñeza et al. 2010), and 80–90 species recorded on the extreme western part of the island (Brown et al. *unpublished data*). Despite a paucity of information, the existence of high species diversity on Mindanao has been cited to rival other well-studied areas of the northern portions of the archipelago, where newly compiled levels of species diversity estimates range upwards of 100 species in some areas (Brown et al. 2012a, 2013a).

Because of our continued uncertainty regarding the true herpetological biodiversity of the southern Philippines, we strongly urge continued survey work aimed at providing accurate estimates of species richness in other subcenters of high-diversity within the Mindanao faunal region. Updated and continued assessment of the conservation status of Mindanao species (Diesmos and Brown 2011; Diesmos et al. 2014, 2015) is, likewise, urgently needed. Using new approaches of concentrated herpetological sampling efforts and repeat visits to several historical sites, we continue to ask: (1) how diverse is Mindanao’s herpetological fauna? (2) Are there differences among sites that would suggest ecological and evolutionary processes behind observed patterns? (3) Is the continued treatment of the Mindanao PAIC’s northern islands (e.g. Samar, Leyte) as simple faunal subsets of Mindanao an over simplification? (4) Just how different is the poorly studied fauna of western (Zamboanga del Norte, Zamboanga Sibugay, and Misamis Occidental provinces) and southern Mindanao (Maguindanao, Lanao del Norte, Lanao del Sur, Sultan Kudarat, South Cotabato, and Sarangani provinces) from the now moderately well-known central and northeastern sub-centers of diversity? (5) Do small islands, off the coast of Mindanao harbor endemic species and unrecognized diversity? These and other question should be the focus of a newly invigorated research program focused on the Mindanao faunal region.

To augment the record provided by published literatutre and publically served museum data, we surveyed isolated islands (Camiguin Sur and Dinagat) and mountain ranges spanning several provinces (Misamis Oriental, Agusan del Norte, Agusan del Sur, Surigao del Norte and Surigao del Sur) of northeastern Mindanao (Table 1). Based on our newly synthesized data (Table 2), we have confirmed the occurrence of numerous historically recorded species, and highlight many new distribution records on Dinagat Island, Agusan del Sur, and Misamis Oriental provinces. Our results suggest high herpetofaunal species diversity (approximately 35% of the total Philippine herpetofauna) in northeast Mindanao and adjacent small islands. We fully anticipate that this estimate of species diversity will continue to increase with future survey efforts targeting many inaccessible and/or poorly sampled intervening sites (Fig. 1) for which data currently are unavailable.

High estimates of regional herpetofaunal diversity in the Philippine archipelago have been confirmed in well-studied biogeographically distinct island groups such as Luzon (Brown and Stuart 2012; Brown et al. 2012a, 2013a). Understanding of the processes that have led to high species diversity in part relies on increased quality of geological inference (Hall 1996, 2002; Yumul et al. 2009), an understanding of the routes of colonization of the archipelago (Brown and Guttman 2002; Brown and Siler 2013), and mechanisms associated with *in situ* speciation (i.e., geographical and/or marine barriers, ecological gradients, and habitat heterogeneity (Welton et al. 2009, 2013a; Brown et al. 2013b). Our new appreciation of Mindanao as an aggregate terrain, formed from recent accretion events of paleoisland precursors as well as subduction events associated with the uplift of previously submerged crustal platforms (Hall 1998; Yumul 2003, 2009; Brown and Diesmos 2009), leads us to expect that these same mechanisms, inferred in islands to the north, may have likewise contributed to Mindanao’s within-island provincial diversity in the south (McGuire and Alcala 2000; Sanguila et al. 2011; Brown and Siler 2013; Brown 2015)

From our surveys, we note new Philippine records for native but non-endemic widespread species (those with a portion of their distributions outside the Philippines). These include *Pelophryne brevipes* (Bufonidae), *Chaperina fusca* (Microhylidae), *Polypedates leucomystax*, *Rhacophorus pardalis*, and *Kurixalus appendiculatus* (Rhacophoridae). These species’ distribution data supplement our understanding of the eastern island arc colonization route (Sulu archipelago-Mindanao-Leyte-Samar-Luzon) as the hypothesized entryway to explain the distribution of herpetofaunal species on Mindanao (Brown and Guttman 2002; Gonzales et al. 2014; Brown and Siler 2014), and to other parts of the archipelago today (review: Brown et al. 2013b). Our new species distribution records from the islands of Camiguin Sur and Dinagat (e.g., *Cyrtodactylus mamanwa*, Eutropis cf. indeprensa, Eutropis cf. multicarinata, *Brachymeles vulcani*, *Calliophis* sp.) re-affirm the prediction of faunal colonization from Mindanao to these ephemerally isolated islands via over water dispersal or colonization along land bridges (Inger 1954; Brown and Diesmos 2009). Both, apparently, have contributed to the processes of isolation, divergence, and speciation (Welton et al. 2010; Linkem et al. 2011; Siler et al. 2012b; Barley et al. 2013).

Although we did not explicitly analyze archipelago-wide distributional data, our new survey data (consisting of intensive elevational transects sampling, covering differing habitat types) corroborate the expectation of regional herpetofaunal diversity of Mindanao (Taylor 1928; Inger 1954; Leviton 1963a; Brown and Alcala 1970; Ross and Lazell 1990; Brown et al. 2013b; Gonzales et al. 2014). Recent surveys have uncovered numerous new species on the island (Brown, Diesmos, Sanguila, Siler *unpublished data*), which will supplement our understanding of species distributions in relation to other biogeographically distinct island groups of the archipelago (Brown et al. 2010, 2013b; Siler et al. 2011a; Barley et al. 2013). Our results not only confirmed additional species distribution records (Table 2), but also provided an opportunity for us to identify many herpetofaunal species complexes (27% of the total species included), which will require taxonomic work in the near future (e.g., Sanguila et al. 2011; Barley et al. 2013; Brown and Siler 2014; Brown 2015). Recent studies aimed at resolving species complexes have identified cryptic genetic diversity and important evolutionary units thereby clarifying these species’ taxonomy and conservation status (e.g., Welton et al. 2009; 2010; Sanguila et al. 2011; Linkem and Brown 2013). Such studies have confirmed the importance of using of multiple sources of data for a sound understanding of species complexes (Brown and Guttman 2002; Grismer et al. 2013; Welton et al. 2013a, 2013b; Brown et al. 2014; Siler et al. 2014a–d).

In this study, we have taken a simple, but important first step, aimed at identifying similar issues on Mindanao. Future work necessarily will involve the resolution of species complexes with taxonomic clarifications, via species descriptions and revisionary work. This will provide an important source of data for use in future studies focused on ecology, conservation, and diversity of many Mindanao amphibian and reptile species complexes.

Our current review of Mindanao amphibians and reptiles also serves as a template to address broader conservation issues. Hosting a large percentage of the remaining forested areas in the archipelago, the forests of Mindanao are threatened by commercial (legal) and illegal logging (deforestation), land conversion for agricultural and commercial purposes (Conservation International 2014; Brown 2015), and recently, the outbreak of an infectious disease that threatens the long term survival of amphibian populations (Swei et al. 2011; Brown et al. 2012b; Diesmos et al. 2012). The first two factors occur in the west and south, and also heavily in the east, while the threat of chytridiomycosis is now established in north-central portions of the island. An additional major threat comes in the form of introduced and invasive frog species (Diesmos et al. 2006, 2015), which appear to be having negative effects (extirpation of native species) along the island’s coasts and lowlands (MBS, KC, RMB *personal observations*)

The entire Mindanao landscape, particularly lower elevation forests, has been heavily impacted by widespread deforestation, and climate change has become a particular concern for some Mindanao forest and shrub frogs (Alcala et al. 2012). Aside from anthropogenic factors that threaten Mindanao’s terrestrial biodiversity, one issue requiring attention from conservationists is a wholesale dearth of herpetological species’ occurrence data (Brown et al. 2012b; Diesmos et al. 2014, 2015) throughout Mindanao. Few comprehensive, repeat-visit, site-based surveys are available for the island. Most recent published accounts have been derived from single-visit site-based studies (Amoroso 2000; Tabaranza et al. 2001; Delima et al. 2006, 2007; Ates et al. 2009; Beukema 2010; Nuñeza et al. 2010), which we necessarily view as incomplete (see Brown et al. 2012a, 2013a, for discussion).

Although conservation status assessment efforts attempt to address these factors in current assessments (IUCN 2016; still badly out of date despite recent comprehensive revisions [Diesmos et al. 2014] and based on the Global Amphibian Assessment of 2004), we have revised and supplemented IUCN conservation status of numerous species with new information from field surveys, plus all available occurrence data in the public domain (Diesmos et al. 2014, 2015; Table 2). Based on our new distributional records and natural history information for relevant species, we downgrade (or support the Diesmos et al.’s [2014] downgrading of eight species to lower threat categories (*Platymantis guentheri*, *Limnonectes diuatus*, *Limnonectes parvus*, *Megophrys stejnegeri*, *Theloderma spinosum*, *Philautus acutirostris*, *Philautus worcesteri*, *Philautus surrufus*, and *Rhacophorus bimaculatus*). In addition to providing novel assessments for previously unassesed taxa (*Leptobrachium lumadorum*, *Bronchocela* sp., *Cyrtodactylus mamanwa*, *Brachymeles tiboliorum*, *Sphenomorphus variegatus*, *Lipinia quadrivittata*, *Dendrelaphis philippinensis*, *Calliophis philippina*, and *Psammodynastes pulverulentus*), another predominant trend to our assessments have been the conversion of species of primarily “Least Concern” status to “Data Deficient” (IUCN 2010) due to recent demonstration of taxonomic uncertainty, a lack of information regarding species boundaries, and/or a lack of sufficient species occurrence or natural history data with which to make informed assessments.. Thirty-two of the total 126 species (~20%) recorded in this study require immediate systematic revision before informed conservation status assessments can be undertaken (Table 2). Most of these are cases where we have evidence that a widespread species (*sensu* Taylor [1928], Inger [1954], Leviton [1963a], Brown and Alcala [1970]) actually is composed of several more range-restricted taxa. We anticipate that in many cases, these more geographically limited putative species will be associated with serious conservation concerns.

Our newly revised conservation reassessment of Mindanao species is based on novel species distribution data, combined with all available historical data (Table 2; see also Diesmos et al. 2014, 2015). The new survey data summarized here involved actual field surveys and, as such, should greatly inform existing (IUCN 2016) conservation assessments. Because the available IUCN (2016) assessments were compiled more than a decade ago, were based on secondary sources such as inferred forest cover, and indiscriminately applied and often incorrect assumptions about the natural history of many taxa (e.g., the degree to which species are, or are not, dependent on primary forest), they are now out of date and becoming incrementally ineffectual with time (Brown et al. 2012a, 2013a; Diesmos et al. 2014). Fortunately, the process of conservation status reassessment is underway (Diesmos et al. 2014) and continuing, in an effort to stay current with each major faunal summary. Even in the face of Philippine central government efforts to establish the country’s own, independent red list of endangered species (ACD *unpublished data*), staying current with IUCN (2016) conservation status assesments will be an ongoing challenge for future generations of conservation biologists if the country is to interact with neighboring nations, exchange internationally-relevant information on conservation priorities, or cooperate with global wildlife trade monitoring, law enforcement, and/or conservation agencies (e.g., CITES, USF&W, IUCN, Conservation International).

We have attempted to provide a summary of all available information on the distribution of amphibians and reptiles of northeast Mindanao. This work both presents a state-of-the-art picture of the region’s herpetofauna, but also identifies areas now sorely in need of additional survey work. Prioritization of remaining areas for field-based surveys (pockets of Data Deficient species like those of western Mindanao and the Sulu Archipelago) has not been been critically evaluated in past conservation assessments (IUCN 2016). Although some groups have been the subject of comprehensive taxonomic review, which has then allowed for subsequent conservation status assessment (Brown and Siler 2013; Brown 2015; Siler et al. 2011b, 2012b, 2014c,d), an analysis of suspected cryptic species diversity, and unrecognized (unprotected) new taxa is sorely lacking for approximately 20% of Mindanao’s fauna. Great progress could be made with increased field-based survey work across all of central, southern, and western Mindanao. This mandate identifies a clear and urgent focus for ameliorating the lack of herpetological biodiversity information from the southern Philippines, and promotes the development of effective conservation management plans, tailored to the needs and challenges of individual protected areas and/or biodiversity hotspots, charged with conserving the remarkable herpetological biodiversity of Mindanao Island.
